# Public health impact of disposable electronic cigarettes since 2021: a scoping review

**DOI:** 10.3389/fpubh.2026.1942633

**Published:** 2026-09-21

**Authors:** Kefan Feng, Liu Liu, Tianyi Wang, Aaron W. J. He, Mengke Liu, Yubin Cao, Guopeng Liang

**Affiliations:** 1 Department of Respiratory Care, West China Hospital, Sichuan University, Chengdu, China; 2 State Key Laboratory of Oral Diseases & National Center for Stomatology & National Clinical Research Center for Oral Diseases, West China Hospital of Stomatology, Sichuan University, Chengdu, China; 3 School of Public Health, Li Ka Shing Faculty of Medicine, University of Hong Kong, Hong Kong, Hong Kong SAR, China; 4 Department of Orthodontic Science, Institute of Science Tokyo, Tokyo, Japan; 5 Department of Cognitive Neurobiology, Institute of Science Tokyo, Tokyo, Japan

**Keywords:** adolescent, disposable e-cigarette, nicotine, policy, public health

## Abstract

**Introduction:**

Disposable e-cigarettes have rapidly proliferated since 2021, particularly among adolescents, raising dual public health concerns regarding youth nicotine initiation and potential harm reduction for adult smokers. This scoping review aims to map the available evidence on disposable e-cigarettes across the domains of chemistry, toxicology, epidemiology, and policy, and to elucidate their multi-dimensional public health implications.

**Methods:**

We systematically searched Medline, Embase, CENTRAL, and Web of Science for peer-reviewed studies published in English between January 2021 and February 2026. This scoping review followed PRISMA-ScR guidelines. Studies were included if they involved disposable e-cigarettes and reported results of public health relevance.

**Results:**

132 studies were included and classified into four themes: (1) nicotine, chemical composition, and harm assessment (*n* = 41), (2) usage trends, population characteristics, and associated factors (*n* = 46), (3) effectiveness for smoking cessation (*n* = 14), and (4) relevant policies, regulation, and prevention (*n* = 31). Research findings raise health concerns regarding disposable e-cigarettes, but long-term clinical outcomes are not yet established. Disposable e-cigarettes are prevalent among adolescents and exhibit distinct demographic characteristics. Although they show potential as smoking cessation aids, they may also cause new nicotine dependence. Existing policies may prove limited in preventing adolescent e-cigarette use, necessitating enhanced regulatory oversight.

**Conclusion:**

Post-2021 disposable e-cigarettes present public health challenges alongside considerable evidence gaps, necessitating both comprehensive regulatory actions and urgent prospective cohort studies to address the tension between adolescent use prevention and adult harm reduction.

## 1 Introduction

In recent years, there has been a growing global interest in vaping, which has posed a series of new governance challenges for public health (1). As e-cigarette products have continued to evolve, disposable e-cigarettes in particular have seen a sharp rise in use since 2021, especially among adolescents. Between January 2021 and April 2022, the proportion of 18-year-old e-cigarette users who used disposable devices increased from 0.4 to 54.8% (2). Unlike earlier rechargeable or refillable devices, these products are characterized by high-concentration nicotine salts, a wide array of fruit and candy flavors, sleek and portable designs, and relatively low prices. These features have rapidly propelled them to become the most commonly used type of e-cigarette among young consumers (2–6). This trend is reflected not only in a sharp increase in user numbers, but also in patterns of frequent and highly dependent use, placing adolescents at considerable risk of nicotine addiction (7). Notably, this prevalence trend exhibits distinct sociodemographic variations (8–10). Commercial marketing and social pressures jointly shape these differential risks of exposure and addiction (11, 12), necessitating an assessment approach that accounts for the broader social and structural determinants of health.

The public health impact of disposable e-cigarettes is dual in nature. Laboratory studies have confirmed that their aerosol contains high concentrations of nicotine, metal particulates such as nickel and lead, and synthetic cooling agents. These substances have been shown to induce cytotoxicity and oxidative stress, as well as exhibit potential carcinogenicity in cellular and animal models (13–17). At the same time, for adult smokers who have been unable to quit combustible cigarettes, disposable e-cigarettes have shown potential as harm reduction tools or smoking cessation aids owing to their efficient nicotine delivery and the absence of combustion, with some studies reporting increased short-term cessation rates (18, 19). This double-edged nature creates a central public health dilemma that requires balancing the prevention of youth nicotine dependence with the preservation of potential harm-reduction pathways for adult smokers.

Faced with the rapid iteration and market expansion of disposable e-cigarette products, regulatory authorities globally have consistently remained reactive, struggling to keep pace with product evolution (20, 21). Some interventions intended to restrict youth access have, driven by market and industry adaptations, yielded unintended consequences that counter their original objectives (20, 22, 23). Although some countries have begun legislating to ban disposable e-cigarettes, this move has raised concerns about illegal trade, new alternatives, and a return to traditional smoking (24, 25). Achieving coherence across diverse regulatory levers, including product authorization, flavor bans, marketing oversight, and fiscal measures, remains a primary obstacle to curbing the proliferation of next-generation disposable e-cigarettes.

In this context, attention must extend beyond chemical composition and disease risk to encompass product marketing, public sentiment, and policy responses, as these factors shape public health by influencing exposure patterns, driving youth uptake, moderating cessation behaviors, and determining the effectiveness of regulatory interventions. In contrast to a systematic review, a scoping review is better suited to assess the breadth and depth of a body of literature, examining its characteristics and pinpointing evidence gaps to inform key stakeholders. Although existing reviews have systematically summarized the health and regulatory risks of early rechargeable and pod-based devices such as JUUL (26), the new generation of disposable e-cigarettes that has rapidly proliferated since 2021 differs significantly from its predecessors. At present, a critical gap remains in the integration of evidence spanning chemical, epidemiological, and policy dimensions. Therefore, this scoping review addresses four key questions concerning (1) the nicotine content, chemical composition, and identified health risks of disposable e-cigarettes; (2) the prevalence trends, user characteristics, and correlates of use, particularly among adolescents; (3) the clinical effectiveness of these products as smoking cessation aids; and (4) the current policy landscape, public sentiment, and persisting regulatory gaps. By mapping the current evidence landscape, this review aims to inform policy development, assist healthcare and public health professionals in accurately identifying and addressing e-cigarette-related issues, enhance awareness among adolescents and the broader public of this emerging health threat, and advocate for the conduct of further high-quality, prospective research to meet the challenges posed by this rapidly evolving product landscape.

## 2 Methods

This scoping review followed the five-stage methodological framework proposed by Arksey and O’Malley, encompassing defining the research questions, identifying relevant studies, selecting eligible studies, charting the data, and collating, summarizing, and reporting the findings (27).

### 2.1 Identifying relevant studies

A systematic search was conducted in Medline, Embase, Cochrane Central Register of Controlled Trials (CENTRAL), and Web of Science, supplemented by a manual screening of the reference lists of included studies. The search was limited to peer-reviewed primary research and secondary analyses of original data published in English between January 1, 2021, and February 14, 2026, reflecting the period during which next-generation disposable e-cigarettes proliferated across the United States and Europe. The complete search strategy is provided in the Supplementary Table S1.

### 2.2 Study selection

Study eligibility was guided by the Population, Concept, and Context (PCC) framework. The target population comprised individuals who use disposable e-cigarettes, while the context focused on these devices, defined as single-use, non-refillable products containing high-concentration nicotine salt-based e-liquids. Studies involving the modification of disposable e-cigarettes, such as for cannabis vaping, were also included. The concept encompassed public health impacts, including physical health outcomes, behavioral implications, and policy-related dimensions. Studies were excluded if they did not distinguish disposable e-cigarettes from other tobacco or nicotine products, or if they focused exclusively on other products (e.g., combustible cigarettes, snus) without reporting disaggregated findings specifically for disposable devices.

The study selection process comprised two stages, beginning with title and abstract screening followed by a full-text review. At each stage, two independent reviewers (K.F. and T.W.) assessed articles against the eligibility criteria, resolving discrepancies through discussion or, when necessary, consultation with a third reviewer (Y.C.). Full texts of potentially eligible studies were retrieved, and citation details were recorded. Studies excluded at the full-text stage were documented in the Supplementary Table S2, along with the reasons for their exclusion. The process adhered to the Preferred Reporting Items for Systematic Reviews and Meta-Analyses extension for Scoping Reviews (PRISMA-ScR) guidelines. The final list of included studies was verified by three members of the review team.

### 2.3 Charting the data

Data extraction was performed independently by two reviewers (K.F. and T.W.) using a standardized form to ensure consistency and accuracy. Extracted information included author details, year of publication, study title, study design, geographic location, objectives, sample size, participant characteristics, type of disposable e-cigarette used, patterns of use (including duration and frequency), and outcome measures. Studies were coded according to study design and outcome domain to facilitate a descriptive synthesis. Specifically, based on the abstracts and methods sections of the included studies, reviewers assigned each study a single design label reflecting the research method used to address its main research question. A single outcome label was then assigned according to the primary research aim, including chemical and toxicological, direct health effects, epidemiological, behavioral, and policy research. For chemical and toxicological studies, we extracted compound types, concentrations, cytotoxicity thresholds, emission forms, and deposition characteristics. For direct health effects, we extracted acute effects, blood biomarker levels, and short- and long-term outcomes. For epidemiological and behavioral investigations, we extracted prevalence estimates, risk associations, user characteristics, and behavioral features, and synthesized these across populations and product subgroups. For policy and regulatory analyses, we extracted regulatory actions, market responses, compliance outcomes, and stakeholder positions, and conducted a thematic synthesis. Finally, all studies were classified into four thematic domains based on outcomes, with each study assigned to a single domain. Two studies offered supplementary insights for another domain; they were cited only within that corresponding domain, without affecting domain assignment or study counts. Disagreements between reviewers were resolved by a third reviewer (Y.C.). In line with scoping review methodology, formal quality appraisal of included studies was not undertaken.

### 2.4 Collating and summarizing

To address the aims of this scoping review, the extracted data were analyzed to generate a descriptive summary of the characteristics of the included studies, which are presented in tabular, graphical, and narrative formats. Key evidence was then synthesized across the domains of direct health effects, youth uptake, utility in smoking cessation, and policy responses to inform the discussion. This review is reported in accordance with the PRISMA-ScR guidelines, which are provided in the Supplementary Table S3.

## 3 Results

### 3.1 Study selection

A summary of the scoping review search process is shown in Figure 1. We retrieved a total of 2,027 records from electronic databases, from which 1,157 duplicates were removed. Following title and abstract screening of the 870 records against the eligibility criteria, 665 articles were excluded. Full-text assessment was performed on the remaining 205 records, resulting in the exclusion of 73 articles and leaving a final pool of 132 studies for inclusion.

**Figure 1:**
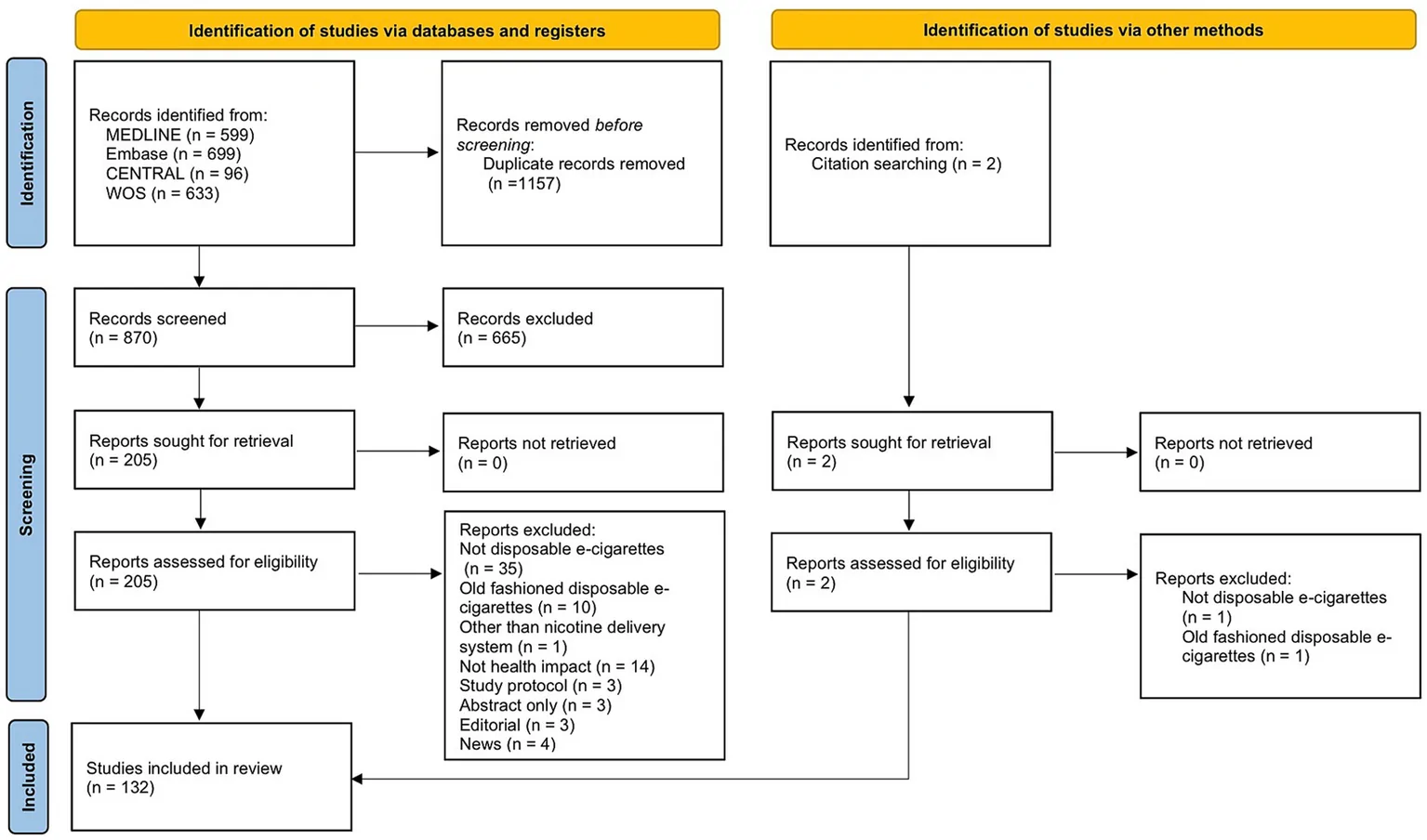
PRISMA diagram showing the flow of potentially eligible studies through screening stages.

### 3.2 Study characteristics

Ultimately, 132 studies met the eligibility criteria and were included in this scoping review. The vast majority of these studies (*n* = 111) were conducted in the United States or the United Kingdom, whereas the remaining articles (*n* = 21) originated from Australia, China, Italy, Canada, Germany, Mexico, Malaysia, or involved multi-country collaborations. Regarding study design, the included studies consisted of 69 cross-sectional studies, 16 longitudinal studies, 34 experimental studies, 2 randomized clinical trials (RCTs), 1 secondary analysis, and 10 content analyses. All included studies were published between 2021 and February 2026, with their baseline characteristics summarized in Figure 2. The included literature was categorized into four domains based on research themes, encompassing (1) nicotine, chemical composition, and harm assessment; (2) usage trends, population characteristics, and associated factors; (3) effectiveness for smoking cessation; and (4) relevant policies, regulation, and prevention (Table 1). Detailed data extraction and thematic classifications for each article are provided in the Supplementary Table S4. Collectively, these domains offer a comprehensive overview of the health profiles and policy implications of disposable e-cigarettes across diverse research perspectives.

**Figure 2:**
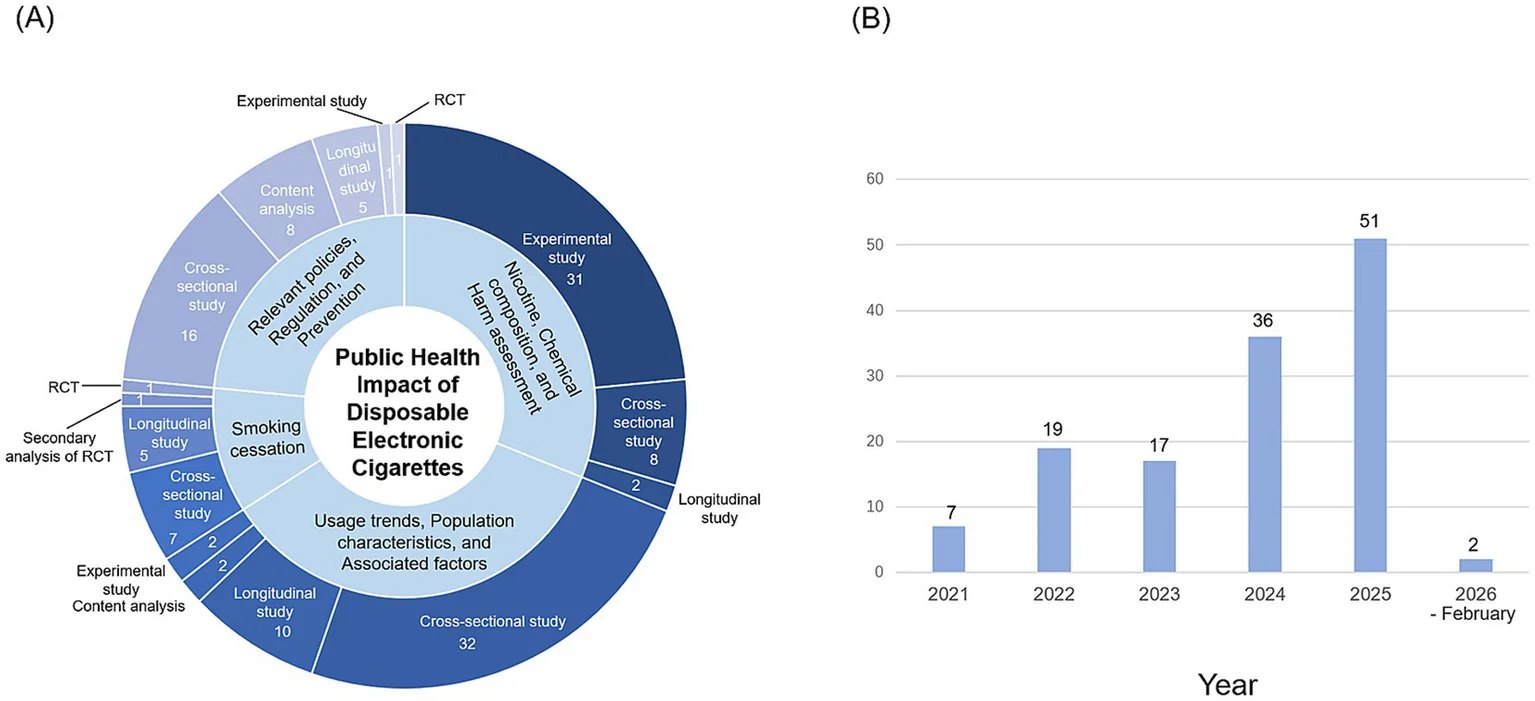
Characteristics of included literature. **(A)** Literature subject classification and research methods. **(B)** Bar chart of literature publication year.

**Table 1:** Overview of key findings, limitations, and evidence gaps on disposable e-cigarettes.

Domain	Number of studies	Study designs	Key findings	Major limitations	Evidence gaps
1. Nicotine, Chemical Composition, and Harm Assessment	41	• 31 experimental • 8 cross-sectional • 2 longitudinal	• High nicotine concentrations with significant variation by brand/region • Wide range of chemicals detected (e.g., WS-23, metals, carbonyls, caffeine) • Many compounds exceed cytotoxic thresholds; metals linked to respiratory and CNS risks • Nicotine delivery efficiency comparable to traditional cigarettes • Limited short-term respiratory/cardiovascular effects reported	• No long-term clinical outcomes (e.g., cancer, COPD, cardiovascular events) • Predominance of *in vitro* and short-term studies • High variability in measurement units across studies	• Long-term health effects with sufficient follow-up • Dose–response relationships between usage patterns and clinical outcomes • Data on passive exposure and bystander health • Health impacts in vulnerable populations (e.g., adolescents, pregnant women)
2. Usage Trends, Population Characteristics, and Associated Factors	46	• 32 cross-sectional • 10 longitudinal • 2 experimental • 2 qualitative	• Rapid increase in disposable e-cigarette use among adolescents and young adults • Strong preference for sweet and fruit flavors • Key drivers: appealing flavors, stylish design, low cost, peer influence • Higher prevalence among females and LGBTQ youth • Mixed findings on race/ethnicity and concurrent tobacco use • Products widely available through physical retail outlets	• Reliance on self-reported data (recall and social desirability bias) • Cross-sectional designs limit causal inference • Geographic concentration in the US and UK • Limited qualitative exploration of user motivations	• Longitudinal trends in diverse geographic and demographic populations • Impact of flavor preferences on long-term use and dependence • Dual-use trajectories with combustible cigarettes
3. Effectiveness for Smoking Cessation	14	• 7 cross-sectional • 5 longitudinal • 1 RCT • 1 secondary analysis	• Disposable e-cigarettes show potential for smoking reduction and cessation • Daily use associated with higher odds of quitting compared to other methods • Users demonstrate strong motivation to quit • Behavioral substitution observed within first 2 weeks of use • Device type may not significantly influence cessation success rates after 2 years • However, may attract non-smokers and promote frequent, compulsive use	• Few RCTs; limited high-quality controlled evidence • Lack of stratification by frequency, duration, and intensity of use • Short follow-up periods; limited long-term cessation data • Variability in cessation outcome definitions • Limited data on dual-use cessation	• Head-to-head RCTs comparing disposable e-cigarettes with other cessation aids • Optimal usage patterns for maximizing cessation success • Long-term cessation outcomes and relapse rates • Impact on non-smokers and young never-smokers
4. Relevant Policies, Regulation, and Prevention	31	• 16 cross-sectional • 8 content analyses • 5 longitudinal • 1 experimental • 1 RCT	• Partial flavor bans led to product substitution (e.g., disposable sales increased nearly 10-fold) • Enforcement gaps persist post-ban; non-compliant sales continue • Manufacturers exploit regulatory loopholes (e.g., “clear” flavors, ambiguous labeling) • Marketing targets youth via social media, in-store placement, and packaging • Standardized packaging reduces appeal among young people • Industry actors resist systemic interventions	• Limited rigorous evaluation of policy impacts • Lack of post-implementation longitudinal data across jurisdictions • Geographic focus on US and UK; limited cross-country comparisons • Minimal assessment of unintended consequences (e.g., illicit markets, smoking relapse)	• Real-world effectiveness of comprehensive vs. partial bans • Impact of marketing restrictions and taxation strategies • Cross-country comparative studies with quasi-experimental designs • Industry adaptation strategies and enforcement mechanisms

### 3.3 Nicotine, chemical composition, and harm assessment

Most studies on the adverse health effects of disposable e-cigarettes were laboratory-based (*n* = 31), focusing on nicotine and other chemical constituents. In contrast, fewer studies employed cross-sectional or longitudinal designs to assess human exposure and epidemiology (*n* = 10). Multiple studies (*n* = 9) analyzing nicotine concentrations across brands used inconsistent units, including mg/g, mg/mL, mg, %, and μg/kg/day (17, 28–35), which presents a major obstacle to regulators in accurately assessing and comparing real-world exposure doses. Overall, e-liquid volume and average nicotine strength in disposable e-cigarettes have risen since 2021, yet substantial variation exists across brands and countries (28, 30, 36–39). For other chemical constituents, laboratory findings demonstrated high consistency. Detected compounds included synthetic coolants (e.g., WS-23, WS-3) (17, 28, 30), metals (e.g., nickel, lead, chromium) (13–15), flavoring chemicals (16, 40–42), solvents, industrial chemicals (32, 35, 42, 43), and thermal degradation products generated during aerosolization (29, 44). Figures 3, 4 display the nicotine, coolant, carbonyl, and metal element levels across different disposable e-cigarette brands, with more extensive nicotine and chemical data available in Supplementary Tables S5, S6.

**Figure 3:**
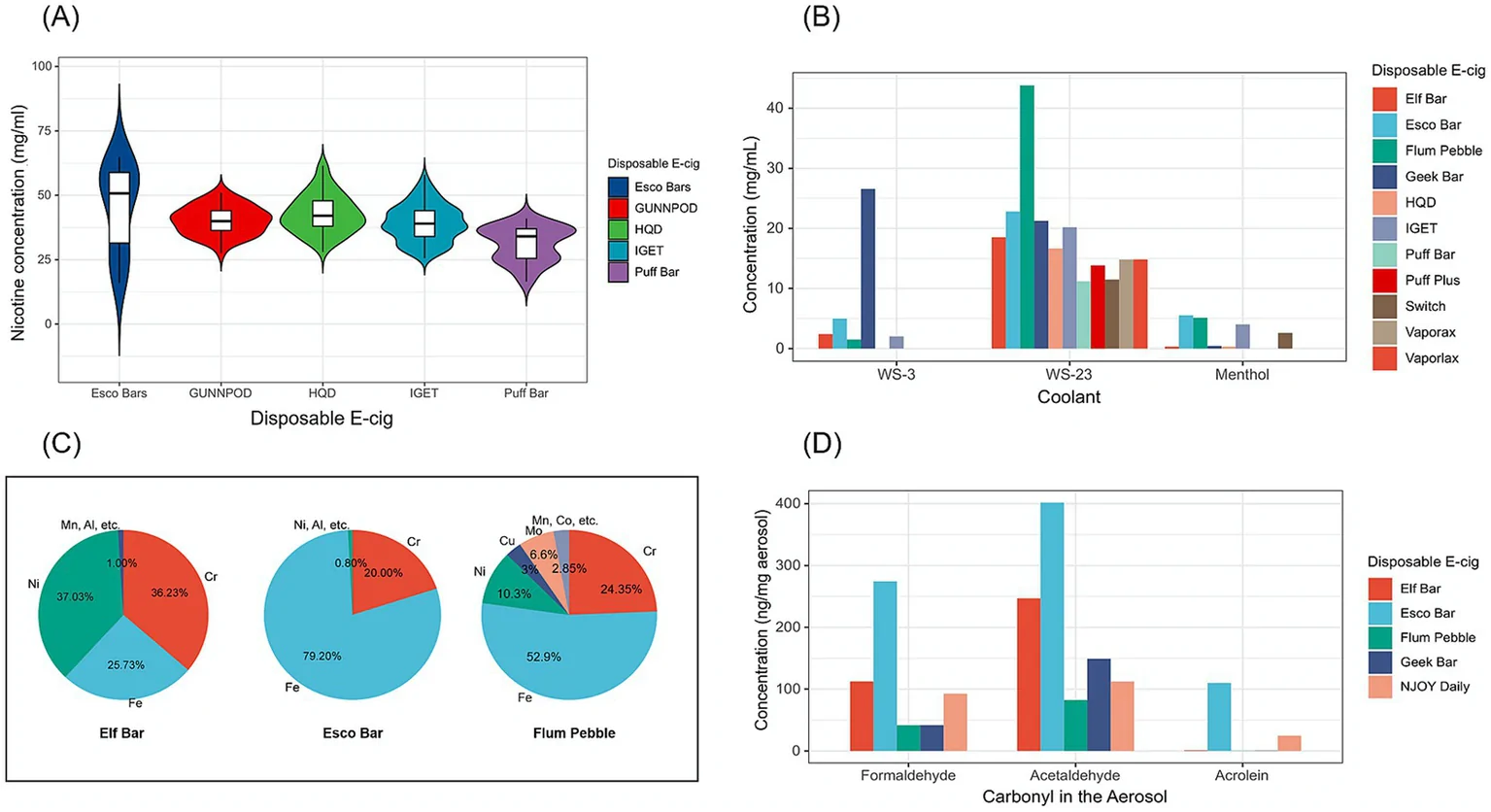
Characteristics of compound levels in different disposable e-cigarettes. **(A)** The measured concentration of nicotine in different brands of disposable e-cigarettes. **(B)** Concentration of cooling additives for different brands of disposable e-cigarettes. **(C)** Metal content of wire coil or mesh coil for different brands of disposable e-cigarettes. **(D)** Carbonyl concentrations in aerosols produced by different brands of disposable e-cigarettes.

**Figure 4:**
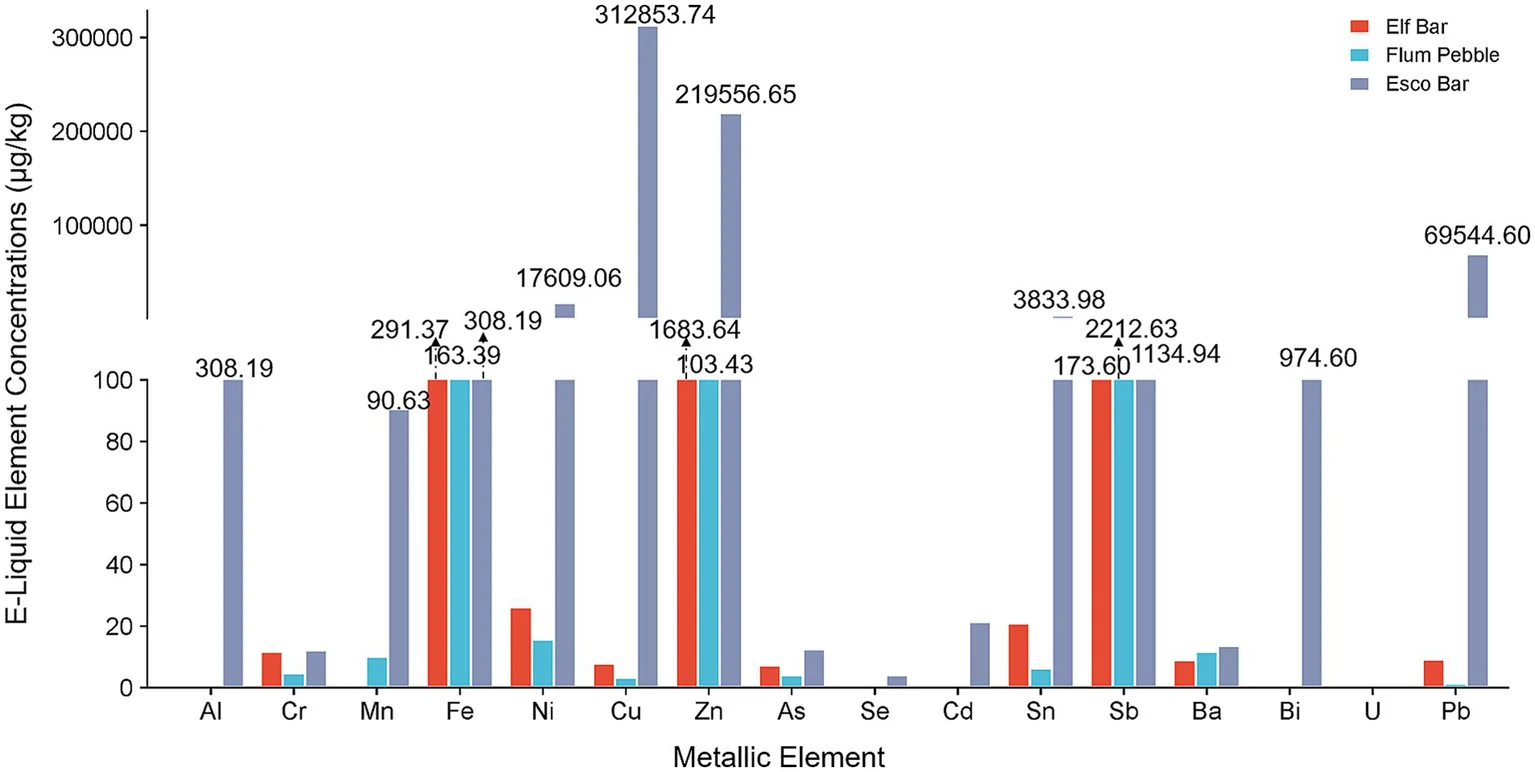
Concentrations of metal elements in different brands of disposable e-cigarette liquid.

Further toxicological studies (*n* = 7) investigated toxicity thresholds, metal speciation in aerosols, and pulmonary deposition modeling (13–16, 30, 35, 45). In the majority of samples, substances such as WS-23, ethyl maltol, and benzoic acid exceeded known cytotoxic thresholds (16, 30, 45). Certain metals in disposable e-cigarette aerosols, such as antimony, were present in carcinogenic forms, whereas chromium and nickel showed higher cancer risk estimates in the risk model. Metal release also increased with frequency of use (13, 35). Simulation experiments have revealed that additives like ethyl maltol, along with metal particles and microplastics, may deposit in the lungs and potentially enter the bloodstream or central nervous system, posing health risks (14–16). Several *in vivo* and *in vitro* studies (*n* = 5) confirmed cytotoxicity, cellular irritation, and the bioaccumulation of certain chemicals (17, 30, 42, 43, 46). *In vitro*, the synthetic cooling agent WS-23 may cause cellular elongation and significantly inhibit cell growth in a concentration-dependent manner (17). *In vivo*, caffeine, tributylphosphine oxide, tributyl O-acetylcitrate, triethyl citrate, vanillin, and isophorone may lead to symptoms of irritation (30, 42). Studies have demonstrated that varying concentrations of H₂O₂ can cause damage to cell membranes, intracellular lipids, enzymes, and DNA within lung epithelial cells. These types of damage may contribute to pulmonary inflammation, cellular injury, and long-term lung impairment, though human evidence remains limited (30, 43).

Human exposure research (*n* = 7) was limited to acute effects and short-term outcomes, including nicotine delivery efficiency, transient cardiovascular changes, and biomarkers of carcinogen exposure relative to combustible cigarette smokers (40, 47–49). Following the use of disposable e-cigarettes, the average increase in plasma nicotine concentration and the maximum concentration (Cmax) were comparable to those from traditional cigarettes, while the time to reach peak concentration (tmax) was shorter. This suggests that disposable e-cigarettes have a comparable potential for dependence (47–49). Users of “clear” e-cigarettes experienced more pronounced increases in systolic blood pressure, diastolic blood pressure, mean arterial pressure, and heart rate following acute use (40). Findings on short-term respiratory symptoms exhibited some inconsistency, though they were generally mild and correlated with puffing frequency (7, 50, 51). These conflicting findings highlight the importance of developing standardized assessment measures and extending follow-up. To date, no study has examined long-term clinical outcomes such as lung cancer, chronic obstructive pulmonary disease (COPD), or major cardiovascular events (*n* = 0), and health risk assessments for bystanders exposed to secondhand aerosols are completely absent (*n* = 0).

### 3.4 Usage trends, population characteristics, and associated factors

This domain encompasses 46 studies, predominantly comprising cross-sectional and longitudinal surveys (*n* = 42). Epidemiological surveys (*n* = 7) consistently demonstrated a sharp increase in disposable e-cigarette use among adolescents globally, establishing these devices as the most prevalent e-cigarette type among youth (3, 52–57). The age group most affected was 17–24 years, with elevated prevalence rates documented among females and LGBTQ+ populations, whereas findings regarding racial and ethnic variations remain mixed (8–10, 58–62).

Multiple studies (*n* = 10) explored factors attracting adolescents to try disposable e-cigarettes, including high nicotine content, low cost, stylish designs, diverse brand imagery, and flavor options, as well as psychological stress, peer influences, family environment, and FDA policies (3, 9, 11, 22, 62–67). Dedicated surveys on flavor preferences among adolescents (*n* = 16) showed that sweet and fruity flavors are the most popular, followed by “ice” or menthol, with tobacco flavors reporting zero utilization (4–6, 53, 68–79). Additional studies (*n* = 11) examined purchase behaviors, perceptions, and usage patterns. Physical retail outlets, such as supermarkets, convenience stores, and gas stations, constituted the primary procurement sources. Concurrently, unauthorized modifications of disposable devices were observed on school campuses (10, 57, 65, 76, 80–83). Regarding perceptions, adults who used e-cigarettes demonstrated high recognition of disposable devices (84) and possessed a good understanding of their harmfulness and addictive potential, although this awareness varied across age groups, usage status, and racial categories (85). Adults in the UK who smoke and/or use e-cigarettes were overall more inclined to perceive disposable devices as more harmful than other types (86).

### 3.5 Effectiveness for smoking cessation

Fourteen studies assessed the effectiveness of disposable e-cigarettes for smoking cessation or harm reduction among adult smokers. Several observational cohort studies (*n* = 5) from Canada, the UK, and the US showed that young adults using disposables exhibited a strong motivation to quit combustible cigarettes (87–91). Epidemiological evidence (*n* = 1) indicated that the rise in disposable use among young people coincided with continued declines in smoking rates (92). Follow-up survey studies (*n* = 4) suggested that disposables could provide short-term substitution but do not affect long-term cessation success rates (19, 89, 92, 93). For smokers averaging 18 cigarettes per day, each additional day of e-cigarette use was associated with a reduction of 0.39 cigarettes smoked. No further significant substitution was observed over the subsequent 6 weeks (19). Cessation success was also associated with the sustained use of disposable devices (94).

The remaining studies (*n* = 4) focused on nicotine dependence and e-cigarette withdrawal. Findings consistently showed that the popularity of disposables has initiated nicotine use among individuals who would otherwise be nicotine-naive, and has driven more addictive, persistent, and frequent patterns of use (7, 95, 96). No statistically significant association existed between device type and successful e-cigarette cessation (97). Based on available evidence, the net public health benefit from reduced smoking versus new nicotine dependence remains uncertain.

### 3.6 Relevant policies, regulation, and prevention

This domain encompassed 31 studies, predominantly cross-sectional and longitudinal surveys (67.7%), evaluating regulatory policies, industry responses, regulatory loopholes, and public health prevention measures related to disposable e-cigarettes.

Eighteen studies assessed FDA policies, California’s flavored tobacco sales ban, and the UK government’s proposed ban on disposables, along with associated policy responses. Over half focused on product substitution following such policies (20–23, 98–103), while others examined market circumvention tactics and consumer and tobacco industry attitudes (24, 25, 104–109). Product substitution under bans was reflected in significant increases in disposable e-cigarette use and market share, rising sales of popular brands, and shifts in online and social media discussions (20–23, 98–103). Partial restrictions on device types and flavors often led to sharp demand shifts. Widespread non-compliance with bans and the use of vague descriptors to circumvent regulations were also prominent (104–107). Policies also raised concerns among consumers and tobacco companies about increased illicit trade and potential smoking relapse (24, 25, 108, 109). Long-term real-world policy effects, such as their efficacy in curbing adolescent nicotine initiation and their potential to inadvertently prompt adult smokers to return to combustible cigarettes, still lack cross-jurisdictional longitudinal data based on quasi-experimental designs.

Public health prevention studies (*n* = 10) primarily examined product packaging and marketing tactics. Cross-sectional surveys in Australia, the UK, Germany, and other countries commonly identified packaging non-compliance, including undeclared nicotine content or concentration, inconsistent units, and discrepancies between labeled and actual content (101, 110–112), potentially undermining consumer risk awareness and informed choice. Regarding marketing tactics, suppliers often blurred the line between recreational products and addictive substances, using discounts and social media promotions to attract youth (12, 113–116). Only one randomized controlled trial demonstrated that “standardized packaging” reduced adolescents’ interest in using e-cigarettes (117).

## 4 Discussion

This scoping review mapped the evidence on the public health impacts of disposable e-cigarettes published since 2021, synthesizing 132 studies across four domains encompassing chemical composition and harm assessment, usage trends and population characteristics, effectiveness for smoking cessation, and policy implications. The results suggest that disposable e-cigarettes pose a multifaceted public health challenge. These devices are characterized by high nicotine concentrations and the presence of numerous potentially hazardous chemical substances, alongside a rapid uptake among younger demographics marked by both social inequalities and geographic variations in accessibility. Evidence on the effectiveness of disposable e-cigarettes as a smoking cessation aid remains inconclusive and conflicting, and current regulatory measures face practical constraints such as industry circumvention and unintended consequences. Taken together, these findings highlight the pressing need for a balanced evaluation of the beneficial and adverse consequences associated with these novel products.

Several public health issues that warrant special concern emerge from the gathered evidence. Chemical and toxicological analyses reveal that disposable e-cigarettes contain high concentrations of nicotine (37), synthetic coolants above cytotoxic levels (16, 17), and metal particulates with potential respiratory deposition (13–15), in addition to the potential generation of carcinogens during aerosolization (29, 44). Epidemiological evidence most consistently points to a sharp rise in adolescent e-cigarette prevalence (3, 52–55). The extremely low cost, enticing fruity flavors, and pervasive distribution through dense physical retail networks have collectively lowered socioenvironmental barriers to access (9, 63–66, 76, 80). Concurrently, the convergence of youth-oriented marketing tactics, psychosocial stressors, and peer influences prevalent among specific groups produces higher objective exposure risks among certain adolescent populations (8–11, 61, 62, 67). At the policy level, manufacturers have responded quickly to bans through product substitution and marketing adaptations (21, 105), while weak regulatory enforcement has led to prominent issues such as non-compliant sales and packaging (12, 101, 104). The bans have also raised public concerns about unintended consequences, including the emergence of illicit markets and relapse to smoking, placing additional strain on public health systems (24, 25).

Beyond these areas of consensus, existing evidence also reveals certain inconsistencies that warrant careful consideration. First, the degree to which disposable e-cigarettes affect respiratory health remains unclear, underscoring the pressing need for additional well-designed, longitudinal observational research (7, 51). Second, research on smoking cessation has revealed a paradox between motivation and outcomes. Disposable e-cigarette users demonstrate stronger motivation in their cessation behaviors, including attempts to completely quit smoking and efforts to reduce consumption (91). However, 2 years later, device type is found to have no significant effect on cessation success rates (92). Third, regarding behavioral patterns, when disposable e-cigarettes are provided to smokers, a marked behavioral substitution phenomenon is observed within the first 2 weeks (19). Nevertheless, studies have also shown that disposable e-cigarettes can foster addictive and compulsive patterns of frequent use (7), and may prompt nicotine initiation among otherwise nicotine-naive individuals (95). The net public health effect of these opposing forces, harm reduction through substitution versus harm creation through new nicotine dependence, remains unclear. Key evidence gaps and variability in study quality further complicate the interpretation of these inconsistencies. Among the included studies, none formally explores the relationship between disposable e-cigarette use and long-term clinical outcomes such as cancer or cardiovascular events, a limitation attributable to their relatively recent market entry. Furthermore, studies investigating the secondhand exposure outcomes of disposable e-cigarettes on bystanders remain notably absent. Additionally, rigorous randomized controlled trials that stratify by frequency of use or compare disposable e-cigarettes with other cessation aids are urgently required to objectively assess their utility in smoking cessation.

The synthesis of the 132 studies included in this scoping review provides key insights for public health strategies, clinical decision-making, and regulatory reform. Over the longer term, the experience of flavor bans across multiple jurisdictions has demonstrated a waterbed effect, with manufacturers swiftly shifting product designs to bypass fragmented regulations. Accordingly, regulators should transition away from reactive, device-specific bans towards proactive, comprehensive measures that cover entire product categories. Proposed approaches include the adoption of standardized plain packaging, strict ceilings on nicotine concentrations, enhanced restrictions on online marketing and social media promotions, and robust compliance monitoring within physical retail environments. Furthermore, since vulnerable youth populations are particularly susceptible to compulsive, high-frequency vaping when exposed to psychological stressors and peer influences, future intervention programs should be closely integrated with mental health services. These programs should offer alternative coping strategies to disrupt the self-reinforcing cycle of social disadvantage and heightened nicotine dependence. Clinically, although disposable e-cigarettes may facilitate short-term abstinence from combustible cigarettes, they also pose risks of persistent dual use and the development of new-onset nicotine dependence. This clinical paradox reinforces the need for timely revisions to treatment guidelines. Practitioners should thus remain judicious in recommending disposable e-cigarettes as harm reduction aids, and should continue to rely on evidence-based, regulated cessation therapies until the long-term safety of these novel products is better characterized.

Several limitations of this scoping review should be acknowledged when interpreting the findings. First, restricting the search to English-language publications may have excluded relevant evidence from non-English speaking jurisdictions, particularly since the majority of included studies originated from the United States and the United Kingdom, thereby potentially limiting the geographic generalizability of the findings. Second, the methodological heterogeneity and quality variability among the included original studies are pronounced. Due to a predominant reliance on cross-sectional designs and self-reported data lacking extended longitudinal tracking, these studies preclude causal inferences, remain susceptible to recall bias and social desirability bias, and render long-term clinical endpoints currently difficult to evaluate. Additionally, the rapid evolution of both disposable e-cigarette products and their corresponding regulatory landscapes implies that findings may quickly become outdated, necessitating continuous surveillance and regular evidence updates. Finally, the absence of a formal quality appraisal, although aligned with scoping review methodologies, prevents the systematic consideration of study quality variations during the interpretation of the synthesized data.

Given the limitations of the current evidence network, future public health research should prioritize four key methodological areas. First, large-scale, multi-country, multicenter prospective cohort studies with long-term follow-up are urgently needed to establish the true attributable risks of long-term clinical outcomes from disposable e-cigarette use, especially among adolescents who initiate early in life. Second, forthcoming trials must move beyond descriptive device characterization by prioritizing well-powered randomized controlled trials that evaluate disposable e-cigarettes against established nicotine replacement therapies, incorporating rigorous dose–response subgroup analyses stratified by puffing topography and daily e-liquid consumption to clarify their efficacy as cessation adjuncts. Third, further investigation is warranted regarding secondhand aerosol exposure, specifically through biomarker-based studies in non-users routinely exposed to aerosol in residential, vehicular, and public settings. Fourth, in view of the accelerated product innovation cycle, policy assessment should extensively employ quasi-experimental designs to enable robust cross-jurisdictional comparisons of real-world regulatory outcomes. Concurrently, regular chemical and toxicological monitoring frameworks must be institutionalized to facilitate early detection and preemptive action before novel synthetic compounds and unidentified additives precipitate widespread population exposure.

## 5 Conclusion

In this scoping review, we synthesized evidence published since 2021 on the public health impacts of disposable e-cigarettes. Disposable e-cigarettes are characterized by high nicotine concentrations and a range of potentially harmful chemicals. Available chemical, toxicological, exposure, and short-term evidence raises significant concerns regarding potential systemic and respiratory adverse effects. For regular smokers, these devices may aid in smoking cessation, as they are associated with fewer acute adverse events, while simultaneously posing substantial risks of nicotine initiation and dependence among youth and non-smokers. Existing policies demonstrate limited effectiveness, remaining marred by persistent challenges in regulatory enforcement and market practices. These findings highlight several areas warranting consideration in future regulatory development, including industry responsiveness, persistent enforcement gaps, and measures to enhance youth awareness and protection. Overall, this review underscores the pressing need for further research to monitor the prevalence, scope, and usage patterns of disposable e-cigarettes across various populations and contexts.

## Data Availability

The original contributions presented in the study are included in the article/Supplementary material, further inquiries can be directed to the corresponding authors.
