# Supplementary material for: Public health impact of disposable electronic cigarettes since 2021: a scoping review
Source: Front Public Health. 2026 Sep 21;14:1942633. doi: 10.3389/fpubh.2026.1942633 (PMC13634833; doi:10.3389/fpubh.2026.1942633)
Supplement: Supplementary file 1 [file Table_1.docx]

Supplementary Material

**Supplementary Material, Table S1. Search strategy of the current study**

**MEDLINE via Ovid**

1	(e-cig$ or ecig$ or electr$ cigar$ or electronic nicotine).mp.

2	(vape or vapes or vaporizer or vapourizer or vaporiser or vapouriser or vaper or vapers or vaping).ti,ab.

3	exp Electronic Nicotine Delivery Systems/

4	1 or 2 or 3

5	disposable or pod$.mp.

6	4 and 5

**Embase via Ovid**

1	(e-cig$ or ecig$ or electr$ cigar$ or electronic nicotine).mp.

2	(vape or vapes or vaporizer or vapourizer or vaporiser or vapouriser or vaper or vapers or vaping).ti,ab.

3	exp Electronic Nicotine Delivery Systems/

4	1 or 2 or 3

5	disposable or pod$.mp.

6	4 and 5

**Cochrane Central Register of Controlled Trials (CENTRAL)**

1	(e-cig$ or ecig$ or electr$ cigar$ or electronic nicotine).mp.

2	(vape or vapes or vaporizer or vapourizer or vaporiser or vapouriser or vaper or vapers or vaping).ti,ab.

3	exp Electronic Nicotine Delivery Systems/

4	1 or 2 or 3

5	disposable or pod$.mp.

6	4 and 5

**Web of Science**

(TS=(e-cig*) OR TS=(ecig*) OR TS=(electr* cigar*) OR TS=(electronic nicotine) OR TI=(vape or vapes or vaporizer or vapourizer or vaporiser or vapouriser or vaper or vapers or vaping) OR AB=(vape or vapes or vaporizer or vapourizer or vaporiser or vapouriser or vaper or vapers or vaping))

AND

(TI=(disposable or pod*) OR AB=(disposable or pod*)) (Document Types: Article or Review Article or Other)

**Supplementary Material, Table S2. Excluded articles and reasons.**

| Reference | Reference | Reason |
| --- | --- | --- |
| (Zhang et al., 2023) | Zhang Q, Jeon J, Goldsmith T, Black M, Greenwald R, Wright C: Characterization of an Electronic Nicotine Delivery System (ENDS) Aerosol Generation Platform to Determine Exposure Risks. TOXICS 2023, 11(2). | No disposable e-cigarettes |
| (Smiley et al., 2021) | Smiley SL, Cho J, Blackman KCA, Cruz TB, Pentz MA, Samet JM, Baezconde-Garbanati L: Retail Marketing of Menthol Cigarettes in Los Angeles, California: a Challenge to Health Equity. Preventing chronic disease 2021, 18:E11-E11. | Old-fashioned disposable electronic cigarettes |
| (McCauley et al., 2023) | McCauley DM, Gaiha SM, Halpern-Felsher B: 169. Developmental and Racial/Ethnic Differences in Use of Nicotine, Non-Nicotine, and THC E-cigarettes. Journal of Adolescent Health 2023, 72(3 Supplement):S95. | abstract only |
| (Holt et al., 2023) | Holt AK, Rudy AK, Sawyer AN, Poklis JL, Breland AB, Peace MR: Survey of U.S. Residents and Their Usage of Electronic Cigarettes with Drugs Other Than Nicotine. Journal of psychoactive drugs 2023:1-10. | Other than nicotine delivery system |
| (Gentzke et al., 2022) | Gentzke AS, Wang TW, Cornelius M, Park-Lee E, Ren C, Sawdey MD, Cullen KA, Loretan C, Jamal A, Homa DM: Tobacco Product Use and Associated Factors Among Middle and High School Students - National Youth Tobacco Survey, United States, 2021. MMWR SURVEILLANCE SUMMARIES 2022, 71(5):1-32. | No disposable e-cigarettes |
| (Yingst et al., 2021) | Yingst JM, Bordner CR, Hobkirk AL, Hoglen B, Allen SI, Krebs NM, Houser KR, Livelsberger C, Foulds J: Response to Flavored Cartridge/Pod-Based Product Ban among Adult JUUL Users: "You Get Nicotine However You Can Get It". INTERNATIONAL JOURNAL OF ENVIRONMENTAL RESEARCH AND PUBLIC HEALTH 2021, 18(1). | No disposable e-cigarettes |
| (Tattan-Birch et al., 2021) | Tattan-Birch H, Brown J, Shahab L, Jackson SE: Trends in use of e-cigarette device types and heated tobacco products from 2016 to 2020 in England. SCIENTIFIC REPORTS 2021, 11(1). | Old-fashioned disposable electronic cigarettes |
| (Gaiha et al., 2021) | Gaiha SM, Lempert LK, McKelvey K, Halpern-Felsher B: 109. Adolescent E-Cigarette Use and Access Before and During the COVID-19 Pandemic. Journal of Adolescent Health 2021, 68(2 Supplement):S57. | abstract only |
| (Cantrell et al., 2021) | Cantrell J, Huang J, Greenberg M, Willett J, Hair E, Vallone D: History and Current Trends in the Electronic Cigarette Retail Marketplace in the United States: 2010-2016. Nicotine and Tobacco Research 2021, 22(5):843-847. | Old-fashioned disposable electronic cigarettes |
| (Bold et al., 2021) | Bold KW, Kong G, Morean M, Gueorguieva R, Camenga DR, Simon P, Davis DR, Jackson A, Krishnan-Sarin S: Trends in various e-cigarette devices used by high school adolescents from 2017-2019. DRUG AND ALCOHOL DEPENDENCE 2021, 219. | Old-fashioned disposable electronic cigarettes |
| (Lin et al., 2020) | Lin C, Baiocchi M, Halpern-Felsher B: Longitudinal trends in e-cigarette devices used by Californian youth, 2014-2018. ADDICTIVE BEHAVIORS 2020, 108. | Old-fashioned disposable electronic cigarettes |
| (Lanza et al., 2020) | Lanza HI, Leventhal AM, Cho J, Braymiller JL, Krueger EA, McConnell R, Barrington-Trimis JL: Young adult e-cigarette use: A latent class analysis of device and flavor use, 2018-2019. Drug and alcohol dependence 2020, 216:108258. | Old-fashioned disposable electronic cigarettes |
| (Taleb et al., 2023) | Taleb ZB, Dabroy D, Akins J, Nelson MD, Kalan ME, Rezk-Hanna M, Matthew Brothers R: Pod-based e-cigarettes versus combustible cigarettes: The impact on peripheral and cerebral vascular function and subjective experiences. Tobacco Induced Diseases 2023, 21(May):71. | No disposable e-cigarettes |
| (Green et al., 2023) | Green DS, Boots B, Olah-Kovacs B, Palma-Diogo D: Disposable e-cigarettes and cigarette butts alter the physiology of an aquatic plant Lemna minor (Lemnaceae). SCIENCE OF THE TOTAL ENVIRONMENT 2023, 892. | Environment impact |
| (Alcantara et al., 2023) | Alcantara C, Chaparro L, Zagury GJ: Occurrence of metals in e-cigarette liquids: Influence of coils on metal leaching and exposure assessment. HELIYON 2023, 9(3). | No disposable e-cigarettes |
| (Glasser et al., 2021) | Glasser AM, Vojjala M, Cantrell J, Levy DT, Giovenco DP, Abrams D, Niaura R: Patterns of E-cigarette Use and Subsequent Cigarette Smoking Cessation Over 2 Years (2013/2014-2015/2016) in the Population Assessment of Tobacco and Health Study. NICOTINE & TOBACCO RESEARCH 2021, 23(4):669-677. | Old-fashioned disposable electronic cigarettes |
| (Rossheim et al., 2020) | Rossheim ME, McDonald KK, Soule EK, Gimm GW, Livingston MD, Barnett TE, Jernigan DH, Thombs DL: Electronic cigarette explosion/burn and poisoning related emergency department visits, 2018-2019. AMERICAN JOURNAL OF EMERGENCY MEDICINE 2020, 38(12):2637-2640. | Old-fashioned disposable electronic cigarettes |
| (Alowedi et al., 2023) | Alowedi S, Wang P, Wang Y, Sharma EF, Sharma S: Immediate effects of e-cigarette use on systemic cardiovascular outcomes and retinal perfusion as measured by OCT angiography: a pilot study. Investigative Ophthalmology & Visual Science 2023, 64(8):3035-3035. | No disposable e-cigarettes |
| (2022) | Nicotine Uptake and Abuse Liability Assessments of 5 Blu Disposable Electronic Cigarettes in Comparison to a Combustible Cigarette. 2022. | Protocal |
| (Tran et al., 2021) | Tran P, Jayawardene W, DeSalle M: E-cigarettes users' quit methods and interventions that may facilitate quitting. Journal of Addiction Medicine 2021, 15(5):E17. | No disposable e-cigarettes |
| (Abadi et al., 2021) | Abadi MH, Lipperman-Kreda S, Shamblen SR, Thompson K, Grube JW, Leventhal AM, Luseno W, Aramburu C: The impact of flavored ENDS use among adolescents on daily use occasions and number of puffs, and next day intentions and willingness to vape. Addictive Behaviors 2021, 114:106773. | No disposable e-cigarettes |
| (Cruz-Jimenez et al., 2025) | Cruz-Jimenez L, Vidana-Perez D, Thrasher JF: Evaluating strategies to reduce effects of e-cigarette social media posts on risk perceptions susceptibility among youth. Tobacco induced diseases 2025, 23:247. | Abstract only |
| (Brown et al., 2025) | Brown J, Colby S, Walker N: Introducing a special issue of rapid research on disposable e-cigarettes. Addiction (Abingdon, England) 2025, 120(3):398-401. | Editorial |
| (Taylor et al., 2025c) | Taylor E, Tattan-Birch H, East K: England's disposable vape ban: An inadequate solution to youth vaping with potential unintended consequences. Addiction (Abingdon, England) 2025, 120(3):402-404. | Editorial |
| (Hammond, 2024) | Hammond D: The rise of disposable e-cigarettes in England and implications for public health. BMJ 2024:q1508. | Editorial |
| (Iacobucci, 2024) | Iacobucci G: Disposable vapes: Paediatricians hail proposed ban. BMJ (Clinical research ed) 2024, 384:q245. | News |
| (Kirby, 2024) | Kirby T: Australia to ban disposable vapes in 2024. The Lancet Respiratory medicine 2024, 12(3):e12. | News |
| (Kmietowicz, 2024) | Kmietowicz Z: Disposable vapes to be banned in England next summer. BMJ (Clinical research ed) 2024, 387:q2356. | News |
| (Manton-Roseblade, 2025) | Manton-Roseblade E: Will banning disposable vapes result in 200000 more smokers? BMJ (Clinical research ed) 2025, 389:r1194. | News |
| (Schiek et al., 2024) | Schiek H, Esch T, Michaelsen MM, Hoetger C: Combining app-based behavioral therapy with electronic cigarettes for smoking cessation: a study protocol for a single-arm mixed-methods pilot trial. Addiction science & clinical practice 2024, 19(1):52. | Protocol |
| (2024) | Compensatory Behavior of E-cig Users in Response to Reduced Nicotine E-liquids. 2024. | Protocol |
| (Do et al., 2025) | Do VV, Shang C, Huang J, Islam T, Pechacek TF, Weaver SR: Volumetric choice experiment to estimate the impact of e-cigarette and heated tobacco product characteristics on substitution and complementary use among adults who smoke cigarettes and recently initiated e-cigarette use. BMJ Open 2025, 15(7):e100073. | Old-fashioned disposable electronic cigarettes |
| (Kasza et al., 2024) | Kasza KA, Rivard C, Goniewicz ML, Fong GT, Hammond D, Cummings KM, Hyland A: E-Cigarette Characteristics and Cigarette Cessation among Adults Who Use E-Cigarettes. JAMA Network Open 2024, 7(8):e2423960. | Old-fashioned disposable electronic cigarettes |
| (Donovan et al., 2025) | Donovan EM, Azadi M, McKay T, Aarvig K, Kreslake J: Not-so-disposable e-cigarettes: Methods young people use to discard single-use e-cigarettes. Addiction (Abingdon, England) 2025, 120(3):452-457. | Environmental or food impact |
| (Dziegiel et al., 2025) | Dziegiel M, Maciejowska A, Misiak M, Lisowska KA: Nicotinism vs. Glomerulopathies-Smoking as a Risk Factor for Primary Glomerulopathies. Antioxidants (Basel, Switzerland) 2025, 14(10). | Environmental or food impact |
| (Lee and Su, 2026) | Lee J, Su WC: A new aerosol respiratory deposition approach: Health risks of metals in aerosols from electronic nicotine delivery systems and heated tobacco products. Journal of Aerosol Science 2026, 191:106709. | Environmental or food impact |
| (Martinez et al., 2025) | Martinez B, Son Y, Khlystov A: Evaluation of carbonyl collection methods in electronic cigarette aerosols. Frontiers in chemistry 2025, 13:1694858. | Environmental or food impact |
| (Ouellette et al., 2024) | Ouellette RR, Morean ME, Lee J, Kong G: Use of ENDS devices and modifications to vape cannabis among adolescents and young adults who also vape nicotine. Addictive Behaviors 2024, 157:108099. | Environmental or food impact |
| (Page et al., 2025) | Page MK, Zhang LY, Leigh NJ, Goniewicz ML, O'Connor RJ: Disposable nicotine vaping products now offer adjustable levels of flavour and coolness. Tobacco control 2025. | Environmental or food impact |
| (Pulvers et al., 2024) | Pulvers K, Jamalian N, Suh E, Faltaoos P, Stewart SL, Aston ER: Nicotine and cannabis routes of administration and dual use among U.S. young adults who identify as Hispanic, non-Hispanic Black, and non-Hispanic White. Preventive Medicine Reports 2024, 48:102912. | Environmental or food impact |
| (Schillo et al., 2024) | Schillo BA, Bertrand A, Briggs J, Kierstead EC, Silver NA, Yoon SN, Diaz MC: Analysis of e-cigarette warning letters issued by the Food and Drug Administration in 2020 and 2021. Tobacco control 2024, 33(2):247-251. | Environmental or food impact |
| (Sharma et al., 2025) | Sharma E, Dubsky J, Garcia-Rosales KV, Halenar M, Benson AF, Maki A, Ruybal AL, Morse AL, Sawdey MD, Alrefai-Kirkpatrick R et al: Disposal practices of cigarettes and electronic nicotine products among adults, findings from Wave 6 (2021) of the PATH Study. PLOS ONE 2025, 20(12 December):e0338007. | Environmental or food impact |
| (Syrjanen et al., 2024) | Syrjanen R, Schumann JL, Castle JW, Sharp L, Griffiths A, Blakey K, Dutch M, Maplesden J, Greene SL: Protonitazene detection in two cases of opioid toxicity following the use of tetrahydrocannabinol vape products in Australia. Clinical Toxicology 2024, 62(8):539-541. | Environmental or food impact |
| (Timmerman et al., 2025) | Timmerman A, Deventer M, Andrews R, Reid R, Marland V, Edwards D, Pudney C, NicDaeid N, Stove C, Norman C: Waxy- or Putty-Like Materials as a Novel Drug Preparation for Synthetic Cannabinoid Receptor Agonists: Detection in Prisons and In Vitro Cannabinoid Receptor Activity. Drug Testing and Analysis 2025, 17(7):1118-1130. | Environmental or food impact |
| (Boots et al., 2024) | Boots B, Green DS, Wright ACM, Olah-Kovacs B, Tovey L: Ecotoxicological effects of leachate from e-cigarettes and e-liquid on the performance of perennial ryegrass (Lolium perenne). Environmental Pollution 2024, 348:123888. | Environmental or food impact |
| (Green et al., 2024) | Green DS, Boots B, Tingey T: Rapid, detrimental response of estuarine benthic macrofauna communities to pollution by littered cigarette filters and e-liquid. Marine Pollution Bulletin 2024, 209:117208. | Environmental or food impact |
| (Avila et al., 2024) | Avila JC, Maglalang DD, Nollen NL, Lee SC, Suh R, Malone M, Binjrajka U, Ahluwalia JS: Using pod based e-cigarettes and nicotine pouches to reduce harm for adults with low socioeconomic status who smoke: a pilot randomized controlled trial. Nicotine & tobacco research 2024, 26(9):1150. | No disposable e-cigarettes |
| (Belderson et al., 2024) | Belderson P, Ward E, Pope I, Notley C: Selecting an e-cigarette for use in smoking cessation interventions and healthcare services: findings from patient and public consultation for the COSTED trial. BMJ open 2024, 14(3):e078677. | No disposable e-cigarettes |
| (AlMuraikhi et al., 2025) | AlMuraikhi HM, Chokor FAZ, Al-Hamdani M: Correlates of perceived social unacceptability of vaping among regular e-cigarette users: a cross-sectional study of a sample of Middle Eastern countries. Frontiers in public health 2025, 13:1620863. | No disposable e-cigarettes |
| (Beard et al., 2024) | Beard JM, Collom C, Liu JY, Obiako P, Strongin RM, Zavala J, Sayes CM: In vitro toxicity and chemical analysis of e-cigarette aerosol produced amid dry hitting. Toxicology 2024, 506:153865. | No disposable e-cigarettes |
| (Brouwer et al., 2025) | Brouwer AF, Jeon J, Jimenez-Mendoza E, Land SR, Holford TR, Friedman AS, Tam J, Mistry R, Levy DT, Meza R: Changing patterns of cigarette and ENDS transitions in the USA: a multistate transition analysis of adults in the PATH Study in 2017-2019 vs 2019-2021. Tobacco control 2025, 34(6):783-790. | No disposable e-cigarettes |
| (Cao et al., 2025) | Cao Y, Hu Z, Li J, Chen X, Xiong Y, Zheng F, Zhang J, Zhang L, Liu X: Assessing Nicotine Pharmacokinetics of New-Generation Tobacco Products and Conventional Cigarettes: A Systematic Review and Meta-analysis. Nicotine and Tobacco Research 2025, 27(5):783-793. | No disposable e-cigarettes |
| (Easwaran et al., 2024) | Easwaran M, Maria CS, Martinez JD, Hung B, Yu X, Soo J, Kimura A, Gross ER, Erickson-DiRenzo E: Effects of Short-term Electronic(e)-Cigarette Aerosol Exposure in the Mouse Larynx. Laryngoscope 2024, 134(3):1316-1326. | No disposable e-cigarettes |
| (Farra et al., 2024) | Farra YM, Matz J, Wilker H, Kim H, Rivera C, Vlahos J, Ramkhelawon B, Oakes JM, Bellini C: Chronic Pod-Mod E-Cigarette Aerosol Exposure Induces Aortic Dysfunction in Hypercholesterolemic Mice: Role of Oxidative Stress and Inflammation. bioRxiv 2024. | No disposable e-cigarettes |
| (Frie et al., 2024) | Frie JA, McCunn P, Eed A, Hassan A, Luciani KR, Chen C, Tyndale RF, Khokhar JY: Factors influencing JUUL e-cigarette nicotine vapour-induced reward, withdrawal, pharmacokinetics and brain connectivity in rats: sex matters. Neuropsychopharmacology 2024, 49(5):782-795. | No disposable e-cigarettes |
| (Gale et al., 2024) | Gale A, Kelly M, Belfield JB, Williams N, Fisher M, Guarraci FA, Stokes JA: Prepubescent Electronic Cigarette Exposure Affects Sexual Motivation and Puberty in Female but Not Male Long-Evans Rats. Nicotine and Tobacco Research 2024, 26(11):1463-1471. | No disposable e-cigarettes |
| (Galimov et al., 2024) | Galimov A, Albers L, Rahman T, Vassey J, Kirkpatrick MG, Unger JB: Assessing the public discourse on Twitter: Reactions to the JUUL e-cigarettes ban in the United States. Tobacco Induced Diseases 2024, 22. | No disposable e-cigarettes |
| (Higgins et al., 2024) | Higgins ST, Sigmon SC, Tidey JW, Heil SH, Gaalema DE, Lee DC, DeSarno MJ, Klemperer EM, Menson KE, Cioe PA et al: Reduced Nicotine Cigarettes and E-Cigarettes in High-Risk Populations: 3 Randomized Clinical Trials. JAMA network open 2024, 7(9):e2431731. | No disposable e-cigarettes |
| (Jackson et al., 2024a) | Jackson SE, Cox S, Shahab L, Brown J: Trends and patterns of dual use of combustible tobacco and e-cigarettes among adults in England: a population study, 2016-2024. medRxiv 2024. | No disposable e-cigarettes |
| (Kapiamba et al., 2025) | Kapiamba KF, Chuang HY, Hao W, Lin TC, Chen LC, Huang YW, Wang Y: Evaluating the Toxicity of Electronic Cigarette Aerosols for Firsthand and Secondhand Exposure Under Different Device Operating Conditions. Chemical Research in Toxicology 2025, 38(8):1344-1356. | No disposable e-cigarettes |
| (Lin et al., 2026) | Lin MY, Abdelfattah LI, Hanchate AD, Sutfin EL, Denlinger-Apte RL: State-Level Flavored E-Cigarette Bans and Initiation Rates Among Youths and Adults. JAMA network open 2026, 9(1):e2551744. | No disposable e-cigarettes |
| (Schiek et al., 2025) | Schiek H, Esch T, Hoetger C: Initial assessment of a novel smoking cessation program integrating app-based behavioral therapy and an electronic cigarette: results of a pilot study. Addiction science & clinical practice 2025, 20(1):31. | No disposable e-cigarettes |
| (Tackett et al., 2025) | Tackett AP, Han DH, Peraza N, Whaley RC, Mason T, Cahn R, Hong K, Pang R, Monterosso J, Page MK et al: Effects of 'Ice' flavoured e-cigarettes with synthetic cooling agent WS-23 or menthol on user-reported appeal and sensory attributes. Tobacco control 2025, 34(2):175. | No disposable e-cigarettes |
| (Vilcassim et al., 2025) | Vilcassim MJR, Stowe S, Zierold KM: Perception of Health Risks of Electronic Cigarette Use Among College Students: Examining the Roles of Sex, Field of Study, Vaping Device Type, and Their Associations. Journal of community health 2025, 50(1):23-30. | No disposable e-cigarettes |
| (Christen et al., 2024) | Christen SE, Hermann L, Bekka E, Vonwyl C, Hammann F, van der Velpen V, Eap CB, Benowitz NL, Haschke M, Liakoni E: Pharmacokinetics and pharmacodynamics of inhaled nicotine salt and free-base using an e-cigarette: a randomized crossover study. Nicotine & tobacco research 2024, 26(10):1313. | No disposable e-cigarettes |
| (Cooper et al., 2024) | Cooper SY, Olszewski NA, Tetteh-Quarshie S, Hill SP, Ghodsi S, Gonzalez-Castro A, Willis CV, Henderson BJ: The Impact of High or Low Doses of Nicotine in a Mouse Model of Vapor Self-Administration. Nicotine and Tobacco Research 2024, 26(3):316-323. | No disposable e-cigarettes |
| (Watkins et al., 2025) | Watkins SL, Snodgrass K, Fahrion L, Shaw E: Contextualizing Changes in e-Cigarette Use During the Early COVID-19 Pandemic and Accompanying Infodemic ("So Much Contradictory Evidence"): Qualitative Document Analysis of Reddit Forums. Journal of Medical Internet Research 2025, 27:e66010. | No disposable e-cigarettes |
| (Taylor et al., 2025a) | Taylor E, Ebdon M, Nottage M, Simonavicius E, Brose L, McNeill A, Arnott D, Cheeseman H, Bunce L, East K: The effect of standardised packaging and limited flavour descriptors of vape pods among adults and youth in Great Britain: a cross-sectional between-subjects experimental study. The lancet regional health - europe 2025, 58. | No disposable e-cigarettes |
| (Lee et al., 2024) | Lee J, Kong G, Krishnan-Sarin S, Camenga DR: Unintended exposure to e-liquids and subsequent health outcomes among US youth and adults. PLoS ONE 2024, 19(7 July):e0308006. | No disposable e-cigarettes |
| (Holt et al., 2024b) | Holt AK, Rudy AK, Sawyer AN, Poklis JL, Breland AB, Peace MR: Survey of U.S. Residents and Their Usage of Electronic Cigarettes with Drugs Other Than Nicotine. Journal of Psychoactive Drugs 2024, 56(4):568-577. | No disposable e-cigarettes |
| (Goldenson et al., 2024) | Goldenson NI, Shiffman S, Sembower MA, Black RA: Assessment of abuse liability and switching potential of menthol-flavored pod-based electronic nicotine delivery systems among US adults who smoke cigarettes. Drug and alcohol dependence 2024, 258:111279. | No disposable e-cigarettes |
| (Edmiston et al., 2022) | Edmiston JS, Webb KM, Wang J, Oliveri D, Liang Q, Sarkar M: Biomarkers of Exposure and Biomarkers of Potential Harm in Adult Smokers Who Switch to e-Vapor Products Relative to Cigarette Smoking in a 24-week, Randomized, Clinical Trial. Nicotine Tob Res 2022, 24(7):1047-1054. | No disposable e-cigarettes |
| (Su et al., 2021) | Su WC, Lin YH, Wong SW, Chen JY, Lee J, Buu A: Estimation of the dose of electronic cigarette chemicals deposited in human airways through passive vaping. J Expo Sci Environ Epidemiol 2021, 31(6):1008-1016. | No disposable e-cigarettes |

**Supplementary Material, Table S3. Preferred Reporting Items for Systematic reviews and Meta-Analyses extension for Scoping Reviews (PRISMA-ScR) Checklist**

| **SECTION** | **ITEM** | **PRISMA-ScR CHECKLIST ITEM** | **REPORTED ON PAGE #** |
| --- | --- | --- | --- |
| **TITLE** | | | |
| Title | 1 | Identify the report as a scoping review. | Title |
| **ABSTRACT** | | | |
| Structured summary | 2 | Provide a structured summary that includes (as applicable): background, objectives, eligibility criteria, sources of evidence, charting methods, results, and conclusions that relate to the review questions and objectives. | Abstract |
| **INTRODUCTION** | | | |
| Rationale | 3 | Describe the rationale for the review in the context of what is already known. Explain why the review questions/objectives lend themselves to a scoping review approach. | Introduction |
| Objectives | 4 | Provide an explicit statement of the questions and objectives being addressed with reference to their key elements (e.g., population or participants, concepts, and context) or other relevant key elements used to conceptualize the review questions and/or objectives. | Introduction |
| **METHODS** | | | |
| Protocol and registration | 5 | Indicate whether a review protocol exists; state if and where it can be accessed (e.g., a Web address); and if available, provide registration information, including the registration number. | Not registered |
| Eligibility criteria | 6 | Specify characteristics of the sources of evidence used as eligibility criteria (e.g., years considered, language, and publication status), and provide a rationale. | 2.1 Identifying relevant studies |
| Information sources* | 7 | Describe all information sources in the search (e.g., databases with dates of coverage and contact with authors to identify additional sources), as well as the date the most recent search was executed. | 2.1 Identifying relevant studies |
| Search | 8 | Present the full electronic search strategy for at least 1 database, including any limits used, such that it could be repeated. | 2.1 Identifying relevant studies |
| Selection of sources of evidence† | 9 | State the process for selecting sources of evidence (i.e., screening and eligibility) included in the scoping review. | 2.2 Study selection |
| Data charting process‡ | 10 | Describe the methods of charting data from the included sources of evidence (e.g., calibrated forms or forms that have been tested by the team before their use, and whether data charting was done independently or in duplicate) and any processes for obtaining and confirming data from investigators. | 2.3 Charting the data |
| Data items | 11 | List and define all variables for which data were sought and any assumptions and simplifications made. | 2.3 Charting the data |
| Critical appraisal of individual sources of evidence§ | 12 | If done, provide a rationale for conducting a critical appraisal of included sources of evidence; describe the methods used and how this information was used in any data synthesis (if appropriate). | 2.4 Collating and summarizing |
| Synthesis of results | 13 | Describe the methods of handling and summarizing the data that were charted. | 2.4 Collating and summarizing |
| **RESULTS** | | | |
| Selection of sources of evidence | 14 | Give numbers of sources of evidence screened, assessed for eligibility, and included in the review, with reasons for exclusions at each stage, ideally using a flow diagram. | 3.1 Study selection |
| Characteristics of sources of evidence | 15 | For each source of evidence, present characteristics for which data were charted and provide the citations. | 3.2 Study characteristics |
| Critical appraisal within sources of evidence | 16 | If done, present data on critical appraisal of included sources of evidence (see item 12). | Non |
| Results of individual sources of evidence | 17 | For each included source of evidence, present the relevant data that were charted that relate to the review questions and objectives. | Results |
| Synthesis of results | 18 | Summarize and/or present the charting results as they relate to the review questions and objectives. | Results |
| **DISCUSSION** | | | |
| Summary of evidence | 19 | Summarize the main results (including an overview of concepts, themes, and types of evidence available), link to the review questions and objectives, and consider the relevance to key groups. | Discussion |
| Limitations | 20 | Discuss the limitations of the scoping review process. | Discussion |
| Conclusions | 21 | Provide a general interpretation of the results with respect to the review questions and objectives, as well as potential implications and/or next steps. | Discussion |
| **FUNDING** | | | |
| Funding | 22 | Describe sources of funding for the included sources of evidence, as well as sources of funding for the scoping review. Describe the role of the funders of the scoping review. | Funding |

JBI = Joanna Briggs Institute; PRISMA-ScR = Preferred Reporting Items for Systematic reviews and Meta-Analyses extension for Scoping Reviews.

* Where *sources of evidence* (see second footnote) are compiled from, such as bibliographic databases, social media platforms, and Web sites.

† A more inclusive/heterogeneous term used to account for the different types of evidence or data sources (e.g., quantitative and/or qualitative research, expert opinion, and policy documents) that may be eligible in a scoping review as opposed to only studies. This is not to be confused with *information sources* (see first footnote).

‡ The frameworks by Arksey and O’Malley (6) and Levac and colleagues (7) and the JBI guidance (4, 5) refer to the process of data extraction in a scoping review as data charting*.*

§ The process of systematically examining research evidence to assess its validity, results, and relevance before using it to inform a decision. This term is used for items 12 and 19 instead of "risk of bias" (which is more applicable to systematic reviews of interventions) to include and acknowledge the various sources of evidence that may be used in a scoping review (e.g., quantitative and/or qualitative research, expert opinion, and policy document).

*From:* Tricco AC, Lillie E, Zarin W, O'Brien KK, Colquhoun H, Levac D, et al. PRISMA Extension for Scoping Reviews (PRISMAScR): Checklist and Explanation. Ann Intern Med. 2018;169:467–473. [doi: 10.7326/M18-0850](http://annals.org/aim/fullarticle/2700389/prisma-extension-scoping-reviews-prisma-scr-checklist-explanation).

| **Supplementary Material, Table S4. Characteristics of all included literature.** | | | | |
| --- | --- | --- | --- | --- |
| **Reference** | **Reference** | **Country** | **Study Type** | **Topic** |
| (Galimov et al., 2021) | Galimov A, Leventhal A, Meza L, et al. Prevalence of disposable pod use and consumer preference for e-cigarette product characteristics among vape shop customers in Southern California: a cross-sectional study. *BMJ Open.* 2021;11(10):e049604. | USA | Cross-sectional study | Flavor appeals |
| (Wang et al., 2021) | Wang TW, Gentzke AS, Neff LJ, et al. Characteristics of e-Cigarette Use Behaviors Among US Youth, 2020. *JAMA Netw Open.* 2021;4(6):e2111336. | USA | Cross-sectional study | Flavor appeals |
| (Zare and Zheng, 2021) | Zare S, Zheng Y. Consumer Preferences for E-cigarette Flavor, Nicotine Strength, and Type: Evidence from Nielsen Scanner Data. *Nicotine Tob Res.* 2021;23(5):823-828. | USA | Cross-sectional study | Flavor appeals |
| (Ali et al., 2024) | Ali FRM, Seaman EL, Diaz MC, Ajose J, King BA. Trends in unit sales of cooling flavoured e-cigarettes, USA, 2017-2021. *Tobacco control.* 2024;33(2):147-153. | USA | Cross-sectional study | Flavor appeals |
| (Chaffee et al., 2022) | Chaffee BW, Halpern-Felsher B, Croker JA, Werts M, Couch ET, Cheng J. Preferences, use, and perceived access to flavored e-cigarettes among United States adolescents and young adults. *Drug Alcohol Depend Rep.* 2022;3. | USA | Cross-sectional study  (online survey) | Flavor appeals |
| (Gaiha et al., 2022) | Gaiha SM, Lempert LK, McKelvey K, Halpern-Felsher B. E-cigarette devices, brands, and flavors attract youth: Informing FDA's policies and priorities to close critical gaps. *Addict Behav.* 2022;126:107179. | USA | Cross-sectional study  (online survey) | Flavor appeals |
| (Han et al., 2022) | Han DH, Lee SH, Seo DC. Association of preferred flavorings and device type with box or pack purchase behavior of electronic nicotine delivery systems in the United States. *J Public Health Policy.* 2022;43(1):65-76. | USA | Cross-sectional study | Flavor appeals |
| (Sargent et al., 2022) | Sargent JD, Stoolmiller M, Dai H, et al. First E-Cigarette Flavor and Device Type Used: Associations With Vaping Persistence, Frequency, and Dependence in Young Adults. *Nicotine Tob Res.* 2022;24(3):380-387. | USA | Cross-sectional study | Flavor appeals |
| (Ali et al., 2023) | Ali FRM, Seaman EL, Crane E, Schillo B, King BA. Trends in US E-cigarette Sales and Prices by Nicotine Strength, Overall and by Product and Flavor Type, 2017-2022. *Nicotine Tob Res.* 2023;25(5):1052-1056. | USA | Cross-sectional study | Flavor appeals |
| (Crespi et al., 2023) | Crespi E, Hardesty JJ, Nian Q, et al. Device and liquid characteristics used with sweet, menthol/mint, and tobacco ENDS liquid flavors: The population-based VAPER study. *Addict Behav.* 2023;144:107727. | USA | Cross-sectional study | Flavor appeals |
| (Jackson et al., 2023) | Jackson SE, Tattan-Birch H, Shahab L, Brown J. How has Expenditure on Nicotine Products Changed in a Fast-Evolving Marketplace? A Representative Population Survey in England, 2018-2022. *Nicotine Tob Res.* 2023;25(9):1585-1593. | UK | Cross-sectional study | Flavor appeals |
| (Jongenelis, 2023) | Jongenelis MI. E-cigarette product preferences of Australian adolescent and adult users: a 2022 study. *BMC Public Health.* 2023;23(1):220. | Australia | Cross-sectional study  (online survey) | Flavor appeals |
| (Leventhal et al., 2023) | Leventhal A, Dai H, Barrington-Trimis J, Sussman S. 'Ice' flavoured e-cigarette use among young adults. *Tob Control.* 2023;32(1):114-117. | USA | Cross-sectional study | Flavor appeals |
| (Vilcassim et al., 2023) | Vilcassim MJR, Jacob D, Stowe S, Fifolt M, Zierold KM. Sex Differences in Electronic Cigarette Device Use Among College Students. *J Community Health.* 2023;48(4):585-592. | UK | Cross-sectional study  (online survey) | Flavor appeals |
| (McDermott et al., 2021) | McDermott MS, East KA, Brose LS, McNeill A, Hitchman SC, Partos TR. The effectiveness of using e-cigarettes for quitting smoking compared to other cessation methods among adults in the United Kingdom. *Addiction.* 2021;116(10):2825-2836. | UK | Longitudinal survey | Cessation effectiveness |
| (R. Miranda et al., 2022) | R. Miranda A, G. Patterson J, LaPolt D, et al. A Call for Regulatory Clarity: “Switch” and “Quit Smoking” Marketing among Online Disposable E-Cigarette Sellers. *HPHR Journal.* 2022(61). | USA | Cross-sectional study | Cessation effectiveness |
| (Gravely et al., 2023) | Gravely S, Yong HH, Reid JL, et al. An examination of quitting smoking as a reason for vaping by the type of nicotine vaping device used most often among adults who smoke and vape: Findings from the Canada, England and the United States 2020 ITC Smoking and Vaping Survey. *Prev Med Rep.* 2023;33:102201. | Canada, UK, USA | Cross-sectional study | Cessation effectiveness |
| (Ranpara et al., 2021) | Ranpara A, Stefaniak AB, Fernandez E, LeBouf RF. Effect of Puffing Behavior on Particle Size Distributions and Respiratory Depositions From Pod-Style Electronic Cigarette, or Vaping, Products. *Front Public Health.* 2021;9:750402. | USA | Experimental study | Chemical Composition and Hazards Assessment |
| (Tehrani et al., 2021) | Tehrani MW, Newmeyer MN, Rule AM, Prasse C. Characterizing the Chemical Landscape in Commercial E-Cigarette Liquids and Aerosols by Liquid Chromatography-High-Resolution Mass Spectrometry. *Chem Res Toxicol.* 2021;34(10):2216-2226. | USA | Experimental study | Chemical Composition and Hazards Assessment |
| (Yogeswaran et al., 2021) | Yogeswaran S, Muthumalage T, Rahman I. Comparative Reactive Oxygen Species (ROS) Content among Various Flavored Disposable Vape Bars, including Cool (Iced) Flavored Bars. *Toxics.* 2021;9(10). | USA | Experimental study | Chemical Composition and Hazards Assessment |
| (Appleton et al., 2022) | Appleton S, Cyrus-Miller H, Seltzer R, Gilligan K, McKinney W. Market survey of disposable e-cigarette nicotine content and e-liquid volume. *BMC Public Health.* 2022;22(1):1760. | USA | Experimental study | Chemical Composition and Hazards Assessment |
| (Fearon et al., 2022) | Fearon IM, Gilligan K, Seltzer RGN, McKinney W. A randomised, crossover, clinical study to assess nicotine pharmacokinetics and subjective effects of the BIDI((R)) stick ENDS compared with combustible cigarettes and a comparator ENDS in adult smokers. *Harm Reduct J.* 2022;19(1):57. | UK | Randomized clinical trial | Cessation effectiveness |
| (Jabba et al., 2022) | Jabba SV, Erythropel HC, Torres DG, et al. Synthetic Cooling Agents in US-marketed E-cigarette Refill Liquids and Popular Disposable E-cigarettes: Chemical Analysis and Risk Assessment. *Nicotine Tob Res.* 2022;24(7):1037-1046. | USA | Experimental study | Chemical Composition and Hazards Assessment |
| (Lin et al., 2022) | Lin HC, Buu A, Su WC. Disposable E-Cigarettes and Associated Health Risks: An Experimental Study. *Int J Environ Res Public Health.* 2022;19(17). | USA | Experimental study | Chemical Composition and Hazards Assessment |
| (Omaiye et al., 2022b) | Omaiye EE, Luo W, McWhirter KJ, Pankow JF, Talbot P. Flavour chemicals, synthetic coolants and pulegone in popular mint-flavoured and menthol-flavoured e-cigarettes. *Tob Control.* 2022;31(e1):e3-e9. | USA | Experimental study | Chemical Composition and Hazards Assessment |
| (Omaiye et al., 2022a) | Omaiye EE, Luo W, McWhirter KJ, Pankow JF, Talbot P. Disposable Puff Bar Electronic Cigarettes: Chemical Composition and Toxicity of E-liquids and a Synthetic Coolant. *Chem Res Toxicol.* 2022;35(8):1344-1358. | USA | Experimental study | Chemical Composition and Hazards Assessment |
| (Sousan et al., 2022) | Sousan S, Pender J, Streuber D, Haley M, Shingleton W, Soule E. Laboratory Determination of Gravimetric Correction Factors for Real-time Area Measurements of Electronic Cigarette Aerosols. *Aerosol Sci Technol.* 2022;56(6):517-529. | USA | Experimental study | Chemical Composition and Hazards Assessment |
| (Talih et al., 2022) | Talih S, Salman R, Soule E, et al. Electrical features, liquid composition and toxicant emissions from 'pod-mod'-like disposable electronic cigarettes. *Tob Control.* 2022;31(5):667-670. | USA | Experimental study | Chemical Composition and Hazards Assessment |
| (Aherrera et al., 2023) | Aherrera A, Lin JJ, Chen R, et al. Metal Concentrations in E-Cigarette Aerosol Samples: A Comparison by Device Type and Flavor. *Environ Health Perspect.* 2023;131(12):127004. | USA | Experimental study | Chemical Composition and Hazards Assessment |
| (Cui et al., 2023) | Cui T, Lu R, Liu Q, Jiang X, Li Y, Pan S. PM1 exposure and spatial transmission of nicotine from the simulated second-hand vapor of pod-based electronic cigarettes. *Sci Total Environ.* 2023;897:165355. | China | Experimental study | Chemical Composition and Hazards Assessment |
| (Jenkins et al., 2023) | Jenkins C, Morgan J, Kelso C. Synthetic Cooling Agents in Australian-marketed E-cigarette Refill Liquids and Disposable E-cigarettes: Trends Follow the US Market. *Nicotine Tob Res.* 2023. | Australia | Experimental study | Chemical Composition and Hazards Assessment |
| (Murphy, 2023) | Murphy S. What e-cigarette factors determine oral epithelial DNA damage in consumers? *Evid Based Dent.* 2023;24(4):163-164. | UK | Experimental study | Chemical Composition and Hazards Assessment |
| (Soule et al., 2023a) | Soule EK, Mayne S, Snipes W, et al. Electronic Cigarette Nicotine Flux, Nicotine Yield, and Particulate Matter Emissions: Impact of Device and Liquid Heterogeneity. *Nicotine Tob Res.* 2023;25(3):412-420. | USA | Cross-sectional study | Chemical Composition and Hazards Assessment |
| (Talih et al., 2023) | Talih S, Karaoghlanian N, Salman R, et al. Effects of Aftermarket Electronic Cigarette Pods on Device Power Output and Nicotine, Carbonyl, and ROS Emissions. *Chem Res Toxicol.* 2023;36(12):1930-1937. | USA | Experimental study | Chemical Composition and Hazards Assessment |
| (Wang et al., 2023) | Wang X, Ghimire R, Shrestha SS, Borowiecki M, Emery S, Trivers KF. Trends in Nicotine Strength in Electronic Cigarettes Sold in the United States by Flavor, Product Type, and Manufacturer, 2017-2022. *Nicotine Tob Res.* 2023;25(7):1355-1360. | USA | Cross-sectional study | Chemical Composition and Hazards Assessment |
| (Hensel et al., 2024) | Hensel EC, Sarles SE, Nuss CJ, et al. Effect of third-party components on emissions from a pod style electronic cigarette. *Toxicological Sciences.* 2024;197(1):104–109. | USA | Experimental study | Chemical Composition and Hazards Assessment |
| (Ruggiero et al., 2022) | Ruggiero JL, Voller LM, Shaik JA, Hylwa S. Formaldehyde in Electronic Cigarette Liquid (Aerosolized Liquid). *Dermatitis.* 2022;33(5):332-336. | USA | Experimental study | Chemical Composition and Hazards Assessment |
| (Boakye et al., 2022) | Boakye E, Dzaye O, Erhabor J, et al. Impact of the Food and Drug Administration enforcement policy on flavored e-cigarettes on the online popularity of disposable e-cigarettes: analyses of Google search query data. *BMC Public Health.* 2022;22(1):1937. | USA | Cross-sectional study | Public Responses to Harms and related policies |
| (Dai and Hao, 2022) | Dai H, Hao J. Online popularity of JUUL and Puff Bars in the USA: 2019-2020. *Tob Control.* 2022;31(1):7-10. | USA | Cross-sectional study | Public Responses to Harms and related policies |
| (Gravely et al., 2022) | Gravely S, Meng G, Hammond D, et al. Electronic nicotine delivery systems (ENDS) flavours and devices used by adults before and after the 2020 US FDA ENDS enforcement priority: findings from the 2018 and 2020 US ITC Smoking and Vaping Surveys. *Tob Control.* 2022;31(Suppl 3):s167-s175. | USA | Longitudinal survey | Public Responses to Harms and related policies |
| (Li et al., 2022) | Li D, Ossip DJ, Travers MB-, Xie Z. Impact of the FDA flavour enforcement policy on flavoured electronic cigarette use behaviour changes. *Tob Control.* 2022;31:s176–s183. | USA | Cross-sectional study  (online survey) | Public Responses to Harms and related policies |
| (Liu et al., 2022) | Liu J, Reynolds MJ, Hartman L, Gottlieb M, Coogan JM, Winickoff JP. The First State Tobacco Flavor Ban: High School Tobacco Control Needs in a Changing Landscape. *J Sch Health.* 2022;92(7):720-727. | USA | Cross-sectional study | Public Responses to Harms and related policies |
| (Chen-Sankey et al., 2023) | Chen-Sankey J, Ganz O, Seidenberg A, Choi K. Effect of a 'tobacco-free nicotine' claim on intentions and perceptions of Puff Bar e-cigarette use among non-tobacco-using young adults. *Tob Control.* 2023;32(4):501-504. | USA | Experimental study | Public Responses to Harms and related policies |
| (Kasza et al., 2023) | Kasza KA, Hammond D, Reid JL, Rivard C, Hyland A. Youth Use of e-Cigarette Flavor and Device Combinations and Brands Before vs After FDA Enforcement. *JAMA Netw Open.* 2023;6(8):e2328805. | USA | Cross-sectional study | Public Responses to Harms and related policies |
| (Schillo et al., 2023) | Schillo B, Seaman EL, Cuccia A, et al. Early evidence of flavored tobacco product restrictions in Massachusetts and New York State. *Tob Induc Dis.* 2023;21:140. | USA | Cross-sectional study | Public Responses to Harms and related policies |
| (Jackson et al., 2024c) | Jackson SE, Tattan-Birch H, Shahab L, et al. Who would be affected by a ban on disposable vapes? A population study in Great Britain. *Public Health.* 2024;227:291-298. | UK | Cross-sectional study | Public Responses to Harms and related policies |
| (Jordt et al., 2024) | Jordt SE, Silinski P, Berman ML. An Electronic Cigarette Pod System Delivering 6-methyl Nicotine, A Synthetic Nicotine Analog, Marketed in the United States as "PMTA Exempt". *Am J Respir Crit Care Med.* 2024;209. | USA | Experimental study | Chemical Composition and Hazards Assessment |
| (Keller-Hamilton et al., 2025) | Keller-Hamilton B, Sloan R, Mehta T, et al. An observational human laboratory assessment of nicotine delivery, vaping topography and subjective effects of usual brand electronic cigarette use among young adults. *Addiction (Abingdon, England).* 2025;120(3):503-513. | USA | Experimental study | Chemical Composition and Hazards Assessment |
| (Minetti et al., 2025) | Minetti ET, Erythropel HC, Keith R, et al. Cardiovascular Health Effects and Synthetic Cooling Agents in E-Cigarettes Labeled as "Clear" Marketed in Massachusetts After the Tobacco Product Flavoring Ban. *J Am Heart Assoc.* 2025;14(16):e036106. | USA | Experimental study | Chemical Composition and Hazards Assessment |
| (Leigh et al., 2025) | Leigh NJ, Page MK, Jamil H, Goniewicz ML. Characteristics and ingredients of disposable 'Elfbar' e-cigarettes sold in the United States and the United Kingdom. *Addiction (Abingdon, England).* 2025;120(3):496-502. | USA and UK | Experimental study | Chemical Composition and Hazards Assessment |
| (Jenkins et al., 2025) | Jenkins C, Powrie F, Kelso C, Morgan J. Chemical Analysis and Flavor Distribution of Electronic Cigarettes in Australian Schools. *Nicotine and Tobacco Research.* 2025;27(6):997-1005. | Australia | Cross-sectional study | Chemical Composition and Hazards Assessment |
| (Wang et al., 2024) | Wang P, Williams RJ, Chen W, et al. Chemical Composition of Electronic Vaping Products From School Grounds in California. *Nicotine and Tobacco Research.* 2024;26(8):991-998. | USA | Experimental study | Chemical Composition and Hazards Assessment |
| (Turner et al., 2024) | Turner A, Scott JW, Backshall-Kennedy T, Dabrowski MC. Deconstructing contemporary disposable vapes: A material and elemental analysis. *Science of the Total Environment.* 2024;954:176292. | UK and USA | Experimental study | Chemical Composition and Hazards Assessment |
| (Craft et al., 2025) | Craft S, Sunderland P, Millea MF, Pudney CR, Sutcliffe OB, Freeman TP. Detection and quantification of synthetic cannabinoids in seven illicitly sourced disposable vapes submitted by an individual presenting to a UK drug and alcohol service. *Addiction (Abingdon, England).* 2025;120(3):549-554. | UK | Cross-sectional study | Chemical Composition and Hazards Assessment |
| (Alam El Din et al., 2025) | Alam El Din SM, Iyengar S, Aherrera AD, et al. Detection of Metal-Containing Particles in FDA-Authorized and New Disposable Electronic Cigarette Aerosols. *Environmental Science and Technology.* 2025;59(46):24707-24716. | USA | Experimental study | Chemical Composition and Hazards Assessment |
| (Falarowski et al., 2025) | Falarowski C, Pieper E, Rabenstein A, et al. Disposable e-cigarettes and their nicotine delivery, usage pattern, and subjective effects in occasionally smoking adults. *Scientific reports.* 2025;15(1):16270. | Germany | Experimental study | Chemical Composition and Hazards Assessment |
| (Pennington and Aldave, 2025) | Pennington C, Aldave SH. Disposable electronic cigarettes: Chemical composition in new and used devices. *Journal of Chromatography A.* 2025;1758:466178. | UK | Experimental study | Chemical Composition and Hazards Assessment |
| (Salazar et al., 2025) | Salazar MR, Saini L, Nguyen TB, et al. Elevated Toxic Element Emissions from Popular Disposable E-Cigarettes: Sources, Life Cycle, and Health Risks. *ACS central science.* 2025;11(8):1345-1354. | USA | Experimental study | Chemical Composition and Hazards Assessment |
| (Robertson et al., 2025) | Robertson NE, Hunsaker HC, Yamamoto M, Cheung K, Poulin BA, Nguyen TB. E-Liquid and Aerosol Characterization of Popular Disposable E-Cigarettes. *ACS omega.* 2025;10(27):29615-29627. | USA | Experimental study | Chemical Composition and Hazards Assessment |
| (Dong et al., 2025) | Dong Z, Pang Q, Ling Q, et al. Heating Coil Corrosion by E-Liquid Containing Nicotine Lactate Salt and In Vitro and In Vivo Evaluation of Nickel Leachate in E-Liquid and E-Cigarette Aerosol. *Chemical Research in Toxicology.* 2025;38(9):1557-1563. | China | Experimental study | Chemical Composition and Hazards Assessment |
| (Svarch-Perez et al., 2024) | Svarch-Perez A, Paz-Gonzalez MV, Ruiz-Juarez C, et al. Methods for a Non-Targeted Qualitative Analysis and Quantification of Benzene, Toluene, and Xylenes by Gas Chromatography-Mass Spectrometry of E-Liquids and Aerosols in Commercially Available Electronic Cigarettes in Mexico. *International Journal of Environmental Research and Public Health.* 2024;21(10):1308. | Mexico | Experimental study | Chemical Composition and Hazards Assessment |
| (Taylor et al., 2025b) | Taylor E, Robson D, Brose LS, et al. Nicotine and tobacco-specific nitrosamine exposure among youth in England who smoke cigarettes and/or vape. *Tobacco control.* 2025. | UK | Cross-sectional study | Chemical Composition and Hazards Assessment |
| (Harris, 2025) | Harris T. Physical and Chemical Characterization of Aerosols Produced from Commercial Nicotine Salt-Based E-Liquids. *Chemical Research in Toxicology.* 2025;38(1):115-128. | USA | Experimental study | Chemical Composition and Hazards Assessment |
| (Yong et al., 2025) | Yong HH, Hughes L, Borland R, et al. Prevalence and correlates of negative side effects from vaping nicotine: Findings from the 2020 ITC four country smoking and vaping survey. *Drug and Alcohol Dependence.* 2025;266:112503. | Canada, USA, UK, Australia | Cross-sectional study | Chemical Composition and Hazards Assessment |
| (Goicoechea et al., 2024) | Goicoechea JZ, Boughner A, Lee JJC, et al. Respiratory symptoms among e-cigarette users without an established smoking history in the VERITAS cohort. *Scientific reports.* 2024;14(1):28549. | Africa , Middle East, USA, Asia-Pacific, Europe | Cross-sectional study | Chemical Composition and Hazards Assessment |
| (Ali et al., 2025) | Ali FRM, Diaz MC, Armour BS, Crane E, Tynan MA, Marynak KL. Trends in U.S. E-cigarette Sales Measured in Milligrams of Nicotine, 2019-2024. *American Journal of Preventive Medicine.* 2025;68(6):1173-1178. | USA | Longitudinal study | Chemical Composition and Hazards Assessment |
| (Rossheim et al., 2024) | Rossheim ME, Tillett KK, Vasilev V, et al. Types and Brands of Derived Psychoactive Cannabis Products: An Online Retail Assessment, 2023. *Cannabis and Cannabinoid Research.* 2024;9(6):1478-1481. | USA | Cross-sectional study | Chemical Composition and Hazards Assessment |
| (Diaz et al., 2025) | Diaz MC, Silver NA, Bertrand A, Schillo BA. Bigger, stronger and cheaper: growth in e-cigarette market driven by disposable devices with more e-liquid, higher nicotine concentration and declining prices. *Tobacco control.* 2025;34(1):65-70. | USA | Longitudinal study | Chemical Composition and Hazards Assessment |
| (Gravely et al., 2025) | Gravely S, Sweanor D, Driezen P, et al. Use of nicotine vaping products during an attempt to quit smoking by Canadian adults who smoke or recently quit: findings from the 2022 Canada International Tobacco Control Four Country Smoking and Vaping Survey. *Utilisation de produits de vapotage contenant de la nicotine lors d'une tentative d'abandon du tabagisme par des adultes canadiens fumeurs ou ayant recemment cesse de fumer : resultats concernant le Canada tires de l'Enquete sur le tabagisme et le vapotage dans quatre pays menee en 2022 par le Projet international d'evaluation de la lutte antitabac.* 2025;45(1):54-60. | Canada | Cross-sectional study | Cessation effectiveness |
| (Holt et al., 2025) | Holt LJ, Petrey AM, Bravo AJ, et al. E-cigarette Quit Attempts in Emerging Adults: Motivations for Attempts and Predictors of Cessation Barriers. *Nicotine and Tobacco Research.* 2025;27(7):1209-1217. | USA | Cross-sectional study | Cessation effectiveness |
| (Bejarano et al., 2024) | Bejarano G, Bluestein MA, Tackett AP, et al. Factors Associated With Successful E-Cigarette Cessation Among a Convenience Sample of Adult Users. *Substance use & misuse.* 2024;59(7):1126-1132. | USA | Cross-sectional study | Cessation effectiveness |
| (Ran et al., 2024) | Ran S, Yang JJ, Piper ME, Lin HC, Buu A. Health Risks Associated with Adopting New-Generation Disposable Products Among Young Adults Who Use E-Cigarettes. *International Journal of Environmental Research and Public Health.* 2024;21(10):1375. | USA | Longitudinal study | Cessation effectiveness |
| (Palmer et al., 2024) | Palmer AM, Rojewski AM, Carpenter MJ, et al. Interest in quitting e-cigarette use by device type and smoking history in US adults. *Tobacco control.* 2024;33(4):537-540. | USA | Cross-sectional study | Cessation effectiveness |
| (Sargent et al., 2024) | Sargent JD, Pratt SI, Brunette MF, Ferron JC, Santos MM, Stoolmiller M. Level and timing of product substitution in a trial of e-cigarettes for smokers not interested in quitting. *Tobacco induced diseases.* 2024;22. | USA | Secondary analysis of RCT | Cessation effectiveness |
| (Erinoso et al., 2024) | Erinoso O, Osibogun O, Li W, Kalan ME. Longitudinal examination of ENDS characteristics, flavors, and nicotine content for cigarette cessation: Findings from PATH waves 5-6. *Addictive Behaviors.* 2024;157:108097. | USA | Longitudinal study | Cessation effectiveness |
| (Kasza et al., 2025) | Kasza KA, Tang Z, Benson AF, et al. Trends in cigarette discontinuation rates in the U.S. across 2014/15-2021 among adults who use electronic nicotine delivery systems (ENDS): Trends by ENDS flavor, ENDS device type, and age group from the PATH Study. *Nicotine & tobacco research : official journal of the Society for Research on Nicotine and Tobacco.* 2025. | USA | Longitudinal study | Cessation effectiveness |
| (Tattan-Birch et al., 2024) | Tattan-Birch H, Brown J, Shahab L, Beard E, Jackson SE. Trends in vaping and smoking following the rise of disposable e-cigarettes: a repeat cross-sectional study in England between 2016 and 2023. *The Lancet Regional Health - Europe.* 2024;42:100924. | UK | Cross-sectional study | Cessation effectiveness |
| (Han et al., 2024) | Han DH, Harlow AF, Feldstein Ewing SW, et al. Disposable E-Cigarette Use and Subsequent Use Patterns in Adolescents and Young Adults. *Pediatrics.* 2024;153(4):e2023063430. | USA | Longitudinal study | Cessation effectiveness |
| (East et al., 2025) | East K, Vu G, Sun T, et al. Harm perceptions across vaping product features: An on-line cross-sectional survey of adults who smoke and/or vape in the United Kingdom. *Addiction (Abingdon, England).* 2025;120(3):524-538. | UK | Cross-sectional study | Flavor and Related Factors |
| (Mattingly et al., 2025) | Mattingly DT, Agbonlahor O, Richardson M, Rayens MK, Rose SW, Hart JL. Factors associated with disposable electronic cigarette use among US youth: A national repeated cross-sectional study, 2021-22. *Addiction (Abingdon, England).* 2025;120(3):432-438. | USA | Cross-sectional study | Flavor and Related Factors |
| (Crespi et al., 2025) | Crespi E, Hardesty JJ, Nian Q, Cohen JE. Differences in E-Cigarette Use Behaviors and Device and Liquid Characteristics of U.S. Adults by Sexual and Gender Identity. *Nicotine and Tobacco Research.* 2025;27(8):1473-1478. | USA | Longitudinal study | Flavor and Related Factors |
| (Chen et al., 2024) | Chen DTH, Girvalaki C, Filippidis FT. Disposable e-cigarette use and associated factors in US middle and high school students, 2021-2022. *Tobacco Induced Diseases.* 2024;22(June). | USA | Cross-sectional study | Flavor and Related Factors |
| (Azagba et al., 2025) | Azagba S, Ebling T, Korkmaz A. Disposable e-cigarette use: Factors, frequency and cigarette smoking among United States high school students. *Addiction (Abingdon, England).* 2025;120(3):423-431. | USA | Cross-sectional study | Flavor and Related Factors |
| (Klosterhalfen et al., 2025) | Klosterhalfen S, Viechtbauer W, Kotz D. Disposable e-cigarettes: Prevalence of use in Germany from 2016 to 2023 and associated user characteristics. *Addiction (Abingdon, England).* 2025;120(3):557-567. | Germany | Cross-sectional study | Flavor and Related Factors |
| (Mulu et al., 2025) | Mulu BA, Berg CJ, Levine H, Abroms LC, Wang Y, Bar-Zeev Y. Factors associated with using disposable versus non-disposable electronic cigarettes among adults in the U.S. and Israel: a cross-sectional study with policy implications. *Israel Journal of Health Policy Research.* 2025;14(1):73. | USA and Israel | Cross-sectional study | Flavor and Related Factors |
| (Ma et al., 2025) | Ma S, Ahn S, Kaareen A, et al. Features of disposable e-cigarettes and their association with pricing and consumer preference: Evidence from web data of US online stores. *Addiction (Abingdon, England).* 2025;120(3):514-523. | USA | Cross-sectional study | Flavor and Related Factors |
| (Nian et al., 2024) | Nian Q, Cohen JE, Sinamo J, Crespi E, Zaman R, Hardesty JJ. Heterogeneity in sociodemographic characteristics of people who use different ENDS devices. *Preventive Medicine Reports.* 2024;37:102561. | USA | Cross-sectional study | Flavor and Related Factors |
| (Li et al., 2025) | Li W, Davis DR, Buta E, et al. "Ice" flavor elements in E-cigarette images: Influence on liking and intention to use among youth who are at risk of E-cigarette use. *Drug and Alcohol Dependence.* 2025;269:112600. | USA | Experimental study | Flavor and Related Factors |
| (Kong et al., 2024) | Kong G, Lee J, Ouellette RR, Morean ME. Modification of Electronic Nicotine Delivery Systems Among Adolescents and Young Adults. *Pediatrics.* 2024;154(1):e2023063984. | USA | Cross-sectional study | Flavor and Related Factors |
| (Donaldson et al., 2025) | Donaldson SI, Beard T, Dormanesh A, et al. Monitoring website marketing among leading e-cigarette brands and vendors in California: content analysis. *Tobacco control.* 2025;34(2):192-197. | USA | Cross-sectional study | Flavor and Related Factors |
| (Jackson et al., 2025a) | Jackson SE, Brown J, Shahab L, Arnott D, Bauld L, Cox S. Nicotine strength of e-liquids used by adult vapers in Great Britain: A population survey 2016 to 2024. *Addiction.* 2025;120(3):468-482. | UK | Cross-sectional study | Flavor and Related Factors |
| (Wang et al., 2026) | Wang S, Nighbor T, Westmaas JL, Asare S, Patel M, Nargis N. Prevalence and Sources of E-Cigarette Use: Findings From a Rapid-Response Survey in High-Tobacco-Burden U.S. States, 2023-2024. *American Journal of Preventive Medicine.* 2026;70(2 Supplement):108062. | USA | Cross-sectional study | Flavor and Related Factors |
| (McCauley et al., 2024) | McCauley DM, Baiocchi M, Gaiha SM, Halpern-Felsher B. Sociodemographic differences in use of nicotine, cannabis, and non-nicotine E-cigarette devices. *Drug and Alcohol Dependence.* 2024;255:111061. | USA | Cross-sectional study | Flavor and Related Factors |
| (Jongenelis et al., 2025) | Jongenelis MI, McCausland K, Bode S. The impact of e-cigarette product characteristics on attitudes and use susceptibility: an experimental study. *Health promotion international.* 2025;40(3). | Australia | Experimental study | Flavor and Related Factors |
| (Jackson et al., 2024b) | Jackson SE, Tattan-Birch H, Shahab L, Brown J. Trends in long term vaping among adults in England, 2013-23: population based study. *BMJ.* 2024:e079016. | UK | Longitudinal study | Flavor and Related Factors |
| (Hammond and Reid, 2025) | Hammond D, Reid JL. Trends in vaping and nicotine product use among youth in Canada, England and the USA between 2017 and 2022: evidence to inform policy. *Tobacco control.* 2025;34(1):115-118. | Canada, UK, and the USA | Cross-sectional study | Flavor and Related Factors |
| (Jackson et al., 2025c) | Jackson SE, Tattan-Birch H, Brown J. Trends in where people buy their vaping products and differences by user and device characteristics: A population study in England, 2016-23. *Addiction (Abingdon, England).* 2025;120(3):539-548. | UK | Cross-sectional study | Flavor and Related Factors |
| (Massey et al., 2025) | Massey ZB, Fairman RT, Cham B, et al. Types of electronic nicotine delivery systems (ENDS) used by people of different age and smoking status groups: results from a US nationally representative survey. *BMJ Open.* 2025;15(2):e087775. | USA | Cross-sectional study | Flavor and Related Factors |
| (Hammond et al., 2025) | Hammond D, Reid JL, Burkhalter R, East K. Use of disposable e-cigarettes among youth who vape in Canada, England and the United States: Repeat cross-sectional surveys, 2017-2023. *Addiction (Abingdon, England).* 2025;120(3):405-413. | Canada, UK, USA | Cross-sectional study | Flavor and Related Factors |
| (Nian et al., 2025) | Nian Q, Hardesty JJ, Crespi E, Cohen JE. Use of multiple devices among U.S. adults frequently using ENDS in 2023. *Drug and Alcohol Dependence.* 2025;268:112580. | USA | Longitudinal study | Flavor and Related Factors |
| (Chen-Sankey et al., 2025) | Chen-Sankey J, Weiger C, La Capria K, et al. Young adults' visual attention to features of social media marketing for disposable e-cigarettes and associated perceptions. *Addiction (Abingdon, England).* 2025;120(3):439-451. | USA | Cross-sectional study | Flavor and Related Factors |
| (Notley et al., 2025) | Notley C, Varley A, Pope I, Dawkins L, Ward E. Young people's use of disposable vapes: A qualitative study. *Addiction (Abingdon, England).* 2025;120(3):458-467. | UK | Content analysis | Flavor and Related Factors |
| (Guerra Castillo et al., 2024) | Guerra Castillo C, Hoeft KS, Couch ET, Urata J, Halpern-Felsher B, Chaffee BW. Adolescents' Experiences and Perceptions of E-Cigarettes and Nicotine Addiction. *Substance use & misuse.* 2024;59(13):1981-1989. | USA | Content analysis | Flavor and Related Factors |
| (Riggs and Church, 2025) | Riggs G, Church TD. Beyond Juul: The New Face of Underage Nicotine Addiction - A Survey of College Students. *Therapeutic Innovation and Regulatory Science.* 2025;59(2):319-327. | USA | Cross-sectional study | Flavor and Related Factors |
| (Douglas et al., 2024) | Douglas AE, Childers MG, Felicione NJ, Milstred AR, Blank MD. Electronic Cigarette Device and Liquid Characteristics: Comparison of Self-Reports to User-Provided Pictures. *Substance use & misuse.* 2024;59(13):1990-1998. | USA | Cross-sectional study | Flavor and Related Factors |
| (Gravely et al., 2024) | Gravely S, Smith TT, Toll BA, et al. Electronic nicotine delivery system flavors, devices, and brands used by adults in the United States who smoke and formerly smoked in 2022: Findings from the United States International Tobacco Control Four Country Smoking and Vaping Survey. *Preventive Medicine Reports.* 2024;47:102905. | USA | Cross-sectional study | Flavor and Related Factors |
| (Huh et al., 2024) | Huh J, Galimov A, Meza L, et al. Evaluating trends in recruitment challenges in vape shop research, e-cigarette product characteristics and use among shop customers from 2019 to 2023: A mixed-methods study. *Tobacco Induced Diseases.* 2024;22. | USA | Cross-sectional study | Flavor and Related Factors |
| (Liu et al., 2024) | Liu J, McCauley D, Gaiha SM, Halpern-Felsher B. Perceptions of Harm and Addictiveness for Nicotine Products, THC e-Cigarettes, and e-Cigarettes with Other Ingredients Among Adolescents, Young Adults, and Adults. *Substance use & misuse.* 2024;59(14):2126-2136. | USA | Cross-sectional study | Flavor and Related Factors |
| (Virgili et al., 2025) | Virgili F, Del Parco F, La Regina DP, et al. Prevalence, risk perception, and motivations behind E-cigarettes and heated tobacco use: a cross-sectional study in Italian adolescents. *Italian Journal of Pediatrics.* 2025;51(1):303. | Italy | Cross-sectional study | Flavor and Related Factors |
| (Ahn et al., 2025) | Ahn S, Ma S, Shang C. Where Do E-cigarette Users Buy Their E-cigarette Products? Results from a Nationally Representative Sample of Adult E-cigarette Users in the United States. *Nicotine & tobacco research : official journal of the Society for Research on Nicotine and Tobacco.* 2025. | USA | Cross-sectional study | Flavor and Related Factors |
| (Ford et al., 2025) | Ford A, MacKintosh AM, Morgan A, et al. A Content Analysis of Nicotine Descriptors on the Front of Vape Packaging in the United Kingdom. *Nicotine and Tobacco Research.* 2025;27(1):152-156. | UK | Content analysis | Related Policies and Regulatory Control Measures |
| (Murthy et al., 2024) | Murthy D, Keshari S, Arora S, et al. Categorizing E-cigarette-related tweets using BERT topic modeling. *Emerging Trends in Drugs, Addictions, and Health.* 2024;4:100160. | USA | Cross-sectional study | Related Policies and Regulatory Control Measures |
| (Donaldson et al., 2024) | Donaldson SI, Beard TA, Trinh J, et al. Content Analysis of E-mail Marketing Communications Among Online E-cigarette Retailers. *Nicotine and Tobacco Research.* 2024;26(9):1248-1252. | USA | Content analysis | Related Policies and Regulatory Control Measures |
| (Pitts et al., 2024) | Pitts SR, Trigger S, Kelley DE. Detecting changes in tobacco product marketplace prominence using social media, advertising, sales, and web traffic data: The example of Puff Bar in the United States tobacco marketplace from 2019 to 2021. *PLoS ONE.* 2024;19(12):e0311723. | USA | Content analysis | Related Policies and Regulatory Control Measures |
| (Fan et al., 2025) | Fan J, Brown J, Cox S, Shahab L, Jackson SE. Level of support for proposed vaping policies: an online survey of UK vapers, 2024. *International Journal of Drug Policy.* 2025;142:104839. | UK | Cross-sectional study | Related Policies and Regulatory Control Measures |
| (Vassey et al., 2024) | Vassey J, Kennedy CJ, Chang HCH, Smith AS, Unger JB. Scalable Surveillance of E-Cigarette Products on Instagram and TikTok Using Computer Vision. *Nicotine and Tobacco Research.* 2024;26(5):552-560. | USA | Longitudinal study | Related Policies and Regulatory Control Measures |
| (Alemu et al., 2025) | Alemu SM, Mons U, Schaller K, Baltaci E, Hoe C. Spatial distribution of e-cigarette vending machines: a case study from Germany. *Tobacco control.* 2025. | Germany | Cross-sectional study | Related Policies and Regulatory Control Measures |
| (Holt et al., 2024a) | Holt AK, Buckmire MG, Moss KS, et al. A multi-year characterization of confiscated vaping products from Virginia school youth. *Journal of Analytical Toxicology.* 2024;48(8):606-615. | USA | Longitudinal study | Related Policies and Regulatory Control Measures |
| (Borowiecki et al., 2024) | Borowiecki M, Kim Y, Emery S. A Patchy Prohibition: Product and Flavor Substitution After the Food and Drug Administration's Prioritized Enforcement Policy on Flavored E-cigarettes. *Nicotine and Tobacco Research.* 2024;26(5):527-535. | USA | Longitudinal study | Related Policies and Regulatory Control Measures |
| (Jackson et al., 2025b) | Jackson SE, Shahab L, Tattan-Birch H, Buss V, Brown J. Changes in vaping trends since the announcement of an impending ban on disposable vapes: A population study in Great Britain. *Addiction.* 2025;120(9):1876-1883. | UK | Cross-sectional study | Related Policies and Regulatory Control Measures |
| (Smith et al., 2025) | Smith MJ, Vaczy C, Hilton S. Co-production of a youth advocacy video on the harms of e-cigarette advertising in Scotland. *Health promotion international.* 2025;40(2). | UK | Content analysis | Related Policies and Regulatory Control Measures |
| (Crespi et al., 2024) | Crespi E, Hardesty JJ, Nian Q, Cohen JE. Decisions of the FDA on premarket tobacco product applications: Changes in the number of unique devices and liquids used by US adults who frequently use electronic nicotine delivery systems, 2020-2023. *Tobacco Induced Diseases.* 2024;22(March). | USA | Longitudinal study | Related Policies and Regulatory Control Measures |
| (Alam et al., 2025) | Alam A, Henriksen L, Johnson TO, Prochaska JJ, Schleicher N. Early assessment of compliance with California's flavoured tobacco sales prohibition: evidence from vape shops. *Tobacco control.* 2025. | USA | Cross-sectional study | Related Policies and Regulatory Control Measures |
| (Bansal-Travers et al., 2025) | Bansal-Travers M, Rivard C, Delnevo CD, et al. Flavor and Device Choices Among People Who Use ENDS: Results From the PATH Study. *American Journal of Preventive Medicine.* 2025;68(2):236-244. | USA | Cross-sectional study | Related Policies and Regulatory Control Measures |
| (Tattan-Birch et al., 2025) | Tattan-Birch H, East K, Cox S, et al. Impact of standardising the colour and branding of vape devices on product appeal among young people: a randomised experiment in England, Canada and the United States. *Tobacco control.* 2025. | UK, Canada, USA | RCT | Related Policies and Regulatory Control Measures |
| (Jenkins et al., 2024c) | Jenkins C, Powrie F, Morgan J, Kelso C. Labelling and composition of contraband electronic cigarettes: Analysis of products from Australia. *International Journal of Drug Policy.* 2024;128:104466. | Australia | Cross-sectional study | Related Policies and Regulatory Control Measures |
| (Shroff and Sreeramareddy, 2024) | Shroff SM, Sreeramareddy CT. Marketing claims, promotional strategies, and product information on Malaysian e-cigarette retailer websites-a content analysis. *Substance abuse treatment, prevention, and policy.* 2024;19(1):11. | Malaysia | Content analysis | Related Policies and Regulatory Control Measures |
| (Kirsch et al., 2024) | Kirsch F, Kröner EM, Lange R, et al. Official test results of disposable e-cigarettes from 2022 in Germany. *Journal of Consumer Protection and Food Safety.* 2024;19(3):339-348. | Germany | Cross-sectional study | Related Policies and Regulatory Control Measures |
| (Nottage et al., 2025) | Nottage MK, Taylor EV, East KA, et al. Packaging of disposable vaping products and e-liquids in England, Canada and the United States: A content analysis. *Addiction (Abingdon, England).* 2025;120(3):483-495. | UK, Canada, USA | Content analysis | Related Policies and Regulatory Control Measures |
| (Lomngam et al., 2025) | Lomngam P, Hiscock R, Evans-Reeves K, Matthes BK. The tobacco industry at the health-environment nexus: a framing analysis of the UK 'disposable' e-cigarette ban. *Health promotion international.* 2025;40(6). | UK | Content analysis | Related Policies and Regulatory Control Measures |
| (Bertrand et al., 2026) | Bertrand A, Diaz MC, Minter T, Goyette R, Schillo BA, Kreslake JM. Clearly flavoured: rising popularity of 'clear' flavoured e-cigarettes and what online descriptions reveal. *Tobacco control.* 2026. | USA | Content analysis | Related Policies and Regulatory Control Measures |

**Supplementary Material, Table S5. Nicotine concentration of different disposable e-cigarette .**

| **Unit** | **Product** | **Nicotine** | **ref** |
| --- | --- | --- | --- |
| **mg/g** | Puff bar Cool Mint | 32.79 ± 0.38 mg/g | (Jabba et al., 2022) |
|  | Puff bar Café Latte | 36.56 ± 0.30 mg/g |  |
|  | Puff bar Orange Ice | 37.61 ± 0.76 mg/g |  |
|  | Puff bar Pomegranate | 29.37 ± 0.47 mg/g |  |
|  | Puff bar Lush Ice | 32.26 ± 0.53 mg/g |  |
|  | Puff bar Melon Ice | 32.81 ± 0.29 mg/g |  |
|  | Puff bar Cucumber | 31.67 ± 0.15 mg/g |  |
|  | Puff bar Lychee | 33.22 ± 0.27 mg/g |  |
|  | Puff bar Menthol | 33.25 ± 0.22 mg/g |  |
|  | Puff bar Blueberry Ice | 32.71 ± 0.45 mg/g |  |
|  | Puff bar Strawberry Banana | 32.12 ± 0.52 mg/g |  |
|  | Puff bar OMG | 34.01 ± 1.35 mg/g |  |
|  | Puff bar Peach Ice | 33.11 ± 0.63 mg/g |  |
|  | Crave clear 5% | 52.4 mg/g | (Minetti et al., 2024) |
|  | Crave ultra clear 5% | 52.2 mg/g |  |
|  | Crave+ kik new clear 5% | 52.5 mg/g |  |
|  | Elfbar clear 5% | 39.1 mg/g |  |
|  | Esco bars clear 5% | 53.0 mg/g |  |
|  | Flum pebble clear 5% | 47.1 mg/g |  |
|  | Flum float clear 5% | 50.2 mg/g |  |
|  | Fogger ultra clear 5% | 34.7 mg/g |  |
|  | Hitt infinite clear 5% | 36.5 mg/g |  |
|  | Hotbox clear 5% | 40.5 mg/g |  |
|  | Uoy bar clear 5% | 32.7 mg/g |  |
|  | Luto clear 2.8% | 28.4 mg/g |  |
|  | Luto smooth clear 5% | 50.0 mg/g |  |
|  | Pacha syn clear 5% | 51.2 mg/g |  |
|  | Podjuice Hyde IQ clear 5% | 50.3 mg/g |  |
|  | Puffbar clear 5% | 36.5 mg/g |  |
|  | Space max pro 5% | 37.7 mg/g |  |
|  | Vozol clear 5% | 37.9 mg/g |  |
|  |  |  |  |
| **mg/mL** | Switch Blue Razz | 34 ± 2 mg/mL | (Jenkins et al., 2023) |
|  | Switch Cucumber Lime | 35 ± 1 mg/mL |  |
|  | Switch Grape | 34.1 ± 0.7 mg/mL |  |
|  | Switch POG | 34 ± 1 mg/mL |  |
|  | Trip Jungle Juice | 34.1 ± 0.2 mg/mL |  |
|  | Xtra Mango Tango | 34.8 ± 0.6 mg/mL |  |
|  | Gunnpod Lychee Ice | 34.8 ± 0.4 mg/mL |  |
|  | HQD Curve Plus Lychee Ice | 32.98 ± 0.08 mg/mL |  |
|  | HQD Blueberry | 38 ± 2 mg/mL |  |
|  | HQD Grapey | 38 ± 1 mg/mL |  |
|  | HQD Lemon Cake | 31 ± 1 mg/mL |  |
|  | HQD Strawberry | 40 ± 2 mg/mL |  |
|  | IGET Apple | 38 ± 2 mg/mL |  |
|  | IGET Bubble Gum | 37 ± 1 mg/mL |  |
|  | IGET Cool Mint | 37 ± 2 mg/mL |  |
|  | IGET Grape | 35.3 ± 0.6 mg/mL |  |
|  | IGET Grapefruit | 36.1 ± 0.8 mg/mL |  |
|  | IGET Guava Ice | 39 ± 1 mg/mL |  |
|  | IGET Guava Ice | 39 ± 3 mg/mL |  |
|  | IGET Lychee Ice | 37 ± 1 mg/mL |  |
|  | IGET Mango | 37.5 ± 0.7 mg/mL |  |
|  | IGET Passion Fruit Mango | 41.8 ± 0.5 mg/mL |  |
|  | IGET Peach Ice | 37 ± 1 mg/mL |  |
|  | IGET Pineapple Ice | 37 ± 1 mg/mL |  |
|  | IGET Strawberry | 37.7 ± 0.7 mg/mL |  |
|  | Puff Bar Blue Razz | 37 ± 2 mg/mL |  |
|  | Puff Bar Cool Mint | 26 ± 1 mg/mL |  |
|  | Puff Bar Lychee Ice | 36 ± 1 mg/mL |  |
|  | Puff Bar Lychee Ice | 36.0 ± 0.9 mg/mL |  |
|  | Puff Bar OMG | 38 ± 3 mg/mL |  |
|  | Puff Bar Pomegranate | 37.8 ± 0.7 mg/mL |  |
|  | Vaporlax Iced Cola | 38 ± 2 mg/mL |  |
|  | Vaporlax Lush Ice | 37 ± 1 mg/mL |  |
|  | Vaporlax Lush Ice | 33.8 ± 0.7 mg/mL |  |
|  | Vaporlax Lush Ice | 36 ± 1 mg/mL |  |
|  | Vaporlax Mango Ice | 34 ± 1 mg/mL |  |
|  | Vaporlax Pink Lemonade | 38.1 ± 0.4 mg/mL |  |
|  | Puff devices | 44.8 mg/mL ± 2.5 | (Omaiye et al., 2022a) |
|  | Ezzy Oval (Berry cool) | 53.8 (0.7) mg/mL | (Talih et al., 2022) |
|  | Ezzy Oval (Mango lychee) | 75.4 (1.39) mg/mL |  |
|  | Hyde (Cherry lemonade) | 86.9 (5.52) mg/mL |  |
|  | Puff Bar (Banana ice) | 83.4 (2.12) mg/mL |  |
|  | SEA (Mint) | 54.3 (0.82) mg/mL |  |
|  | Disposable vapes | 73.4 (14.5) mg/mL | (Soule et al., 2023a) |
|  | Pod mod devices | 48.4 (14.6) mg/mL |  |
|  | Elfbar BC5000 | 41.8 ± 2.3 mg/mL | (Leigh et al., 2025) |
|  | Elfbar 600 | 19.7 ± 1.2 mg/mL |  |
|  | Flum Pebble Clear | 56.0 ± 3.7 mg/mL | (Robertson et al., 2025) |
|  | Flum Pebble Strawmango | 59.4 ± 4.4 mg/mL |  |
|  | Flum Pebble Watermelon Lemon Mint | 16.2 ± 1.2 mg/mL |  |
|  | Flum Pebble Vanilla Ice Cream | 27.9 ± 2.0 mg/mL |  |
|  | Flum Pebble White Gummy | 34.7 ± 2.5 mg/mL |  |
|  | Elf Bar Clear | 53.6 ± 3.9 mg/mL |  |
|  | Elf Bar Watermelon Ice | 51.1 ± 3.7 mg/mL |  |
|  | Elf Bar Blueberry Tobacco | 31.6 ± 4.0 mg/mL |  |
|  | Elf Bar Blue Cotton Candy | 31.2 ± 2.5 mg/mL |  |
|  | Esco Bars Clear | 53.5 ± 3.9 mg/mL |  |
|  | Esco Bars Peach Ice | 50.5 ± 4.0 mg/mL |  |
|  | Esco Bars White Gummy | 25.8 ± 1.9 mg/mL |  |
|  | Esco Bars Tropical Rainbow Blast | 25.0 ± 1.8 mg/mL |  |
|  | Esco Bars Salted Caramel | 50.7 ± 11 mg/mL |  |
|  | Esco Bars Bubblegum Ice | 57.8 ± 4.2 mg/mL |  |
|  | Esco Bars Cotton Candy | 37.0 ± 2.7 mg/mL |  |
|  | Esco Bars Blue Razz Cotton Candy | 61.2 ± 4.2 mg/mL |  |
|  | Esco Bars Jungle Juice (6000) | 59.9 ± 4.2 mg/mL |  |
|  | Esco Bars Peach Watermelon (6000) | 64.9 ± 4.7 mg/mL |  |
|  | Esco Bars Spearmint (6000) | 16.0 ± 1.2 mg/mL |  |
|  | Geek Bar Strawberry Banana | 36.0 ± 2.7 mg/mL |  |
|  | Geek Bar Watermelon Ice | 21.5 ± 1.7 mg/mL |  |
|  | Geek Bar Orange Creamsicle | 27.2 ± 2.0 mg/mL |  |
|  | Geek Bar Miami Mint | 24.8 ± 3.0 mg/mL |  |
|  | Geek Bar B Pop | 29.8 ± 2.2 mg/mL |  |
|  | Switch Blue Razz | 34 ± 2 mg/mL | (Jenkins et al., 2024a) |
|  | Switch Cucumber Lime | 35 ± 1 mg/mL |  |
|  | Switch Grape | 34.1 ± 0.7 mg/mL |  |
|  | Switch POG | 34 ± 1 mg/mL |  |
|  | Trip Jungle Juice | 34.1 ± 0.2 mg/mL |  |
|  | Xtra Mango Tango | 34.8 ± 0.6 mg/mL |  |
|  | Gunpod Lychee Ice | 34.8 ± 0.4 mg/mL |  |
|  | HQD Curve Plus Lychee Ice | 32.98 ± 0.08 mg/mL |  |
|  | HQD Blueberry | 38 ± 2 mg/mL |  |
|  | HQD Grapey | 38 ± 1 mg/mL |  |
|  | HQD Lemon Cake | 31 ± 1 mg/mL |  |
|  | HQD Strawberry | 40 ± 2 mg/mL |  |
|  | IGET Apple | 38 ± 2 mg/mL |  |
|  | IGET Bubble Gum | 37 ± 1 mg/mL |  |
|  | IGET Cool Mint | 37 ± 2 mg/mL |  |
|  | IGET Grape | 35.3 ± 0.6 mg/mL |  |
|  | IGET Grapefruit | 36.1 ± 0.8 mg/mL |  |
|  | IGET Guava Ice | 39 ± 1 mg/mL |  |
|  | IGET Guava Ice | 39 ± 3 mg/mL |  |
|  | IGET Lychee Ice | 37 ± 1 mg/mL |  |
|  | IGET Mango | 37.5 ± 0.7 mg/mL |  |
|  | IGET Passion Fruit Mango | 41.8 ± 0.5 mg/mL |  |
|  | IGET Peach Ice | 37 ± 1 mg/mL |  |
|  | IGET Pineapple Ice | 37 ± 1 mg/mL |  |
|  | IGET Strawberry | 37.7 ± 0.7 mg/mL |  |
|  | Puff Bar Blue Razz | 37 ± 2 mg/mL |  |
|  | Puff Bar Cool Mint | 26 ± 1 mg/mL |  |
|  | Puff Bar Lychee Ice | 36 ± 1 mg/mL |  |
|  | Puff Bar Lychee Ice | 36.0 ± 0.9 mg/mL |  |
|  | Puff Bar O.M.G | 38 ± 3 mg/mL |  |
|  | Puff Bar Pomegranate | 37.8 ± 0.7 mg/mL |  |
|  | Vaporax Iced Cola | 38 ± 2 mg/mL |  |
|  | Vaporax Lush Ice | 37 ± 1 mg/mL |  |
|  | Vaporax Lush Ice | 33.8 ± 0.7 mg/mL |  |
|  | Vaporax Lush Ice | 36 ± 1 mg/mL |  |
|  | Vaporax Mango Ice | 34 ± 1 mg/mL |  |
|  | Vaporax Pink Lemonade | 38.1 ± 0.4 mg/mL |  |
|  |  |  |  |
| **μg/kg/Day**  **(average daily dose)** | Air Bar—Watermelon Ice | 4.21 μg/kg/Day | (Lin et al., 2022) |
|  | Puff Bar—Grape | 1.57 μg/kg/Day |  |
|  |  |  |  |
| **%** | beverage-flavored e-cigarettes | (Strength) 1.2% to 5.3% | (Wang et al., 2023) |
|  | Menthol-flavored e-cigarettes | (Strength) 2.0% to 4.5% |  |
|  | tobacco-flavored e-cigarettes | (Strength) 2.3% to 4.1% |  |
|  | candy or sweet-flavored products from | (Strength) 3.0% to 3.9% |  |
|  | BLOW Disposable Vape Stix (Blueberry Raspberry) | 5.0% | (Ruggiero, 2022) |
|  | BLOW Disposable Vape Stix (Grape) | 5.0% |  |
|  | BLOW Disposable Vape Stix (Mango) | 5.0% |  |
|  | Minnesota Legit Drag (Below Zero) | 10.0% |  |
|  | Minnesota Legit Drag (Purple Rain) | 10.0% |  |
|  |  |  |  |
| **mg** | Puff XXL Pineapple Grape | 108 mg | (Appleton et al., 2022) |
|  | MNGO Stick Strawberry Mango | 31.5 mg |  |
|  | MNGO Stick Blueberry Mango | 32.7 mg |  |
|  | RARE FLOW Mango Ice | 129 mg |  |
|  | MNGO Stick Iced Banana | 34.3 mg |  |
|  | Omega Plus Fruit Blast | 199 mg |  |
|  | Kangvape Banana Ice | 132 mg |  |
|  | Z Stick Menthol | 39.4 mg |  |
|  | RARE FLOW Lemon Ice | 154 mg |  |
|  | Z Stick Strawberry Lemonade | 40.4 mg |  |
|  | MNGO Stick Grape Ice | 42.0 mg |  |
|  | Z Stick Mango | 42.2 mg |  |
|  | MNGO Stick Strawberry Mango | 43.2 mg |  |
|  | MNGO Stick Iced Banana | 43.6 mg |  |
|  | HQD Banana Ice | 35.9 mg |  |
|  | Kangvape Blueberry Lemon | 198 mg |  |
|  | Just Puff Rich Cherry | 44.7 mg |  |
|  | Cube Summer Menthol 550 355 | 355 mg |  |
|  | HQD Blueberry | 42.2 mg |  |
|  | Fume Extra Blue Razz | 219 mg |  |
|  | Air Bar Blue Razz Lemonade | 65.9 mg |  |
|  | POP Lush Ice | 44.3 mg |  |
|  | POP Banana Ice | 48.3 mg |  |
|  | Airbar Lux Blue Raspberry | 109 mg |  |
|  | Vapo 1500 Lemon Lime | 266 mg |  |
|  | King Grape | 272 mg |  |
|  | Vapo 1500 Sorbetto | 252 mg |  |
|  | Vapo Tobacco | 61.1 mg |  |
|  | BIDI Stick Tropic | 72.7 mg |  |
|  | Pop Hit Pro Chilled Watermelon | 52.9 mg |  |
|  | Vapo Kiwi | 31.8 mg |  |
|  | Pop Hit Pro Menthol | 54.1 mg |  |
|  | RUSH Watermelon Ice | 182 mg |  |
|  | BIDI Stick Arctic | 76.7 mg |  |
|  | Cube Energy | 506 mg |  |
|  | RUSH Mango Ice | 195 mg |  |
|  | BIDI Stick Zest 84.0 82.1 | 82.1 mg |  |
|  | Blu Menthol | 23.5 mg |  |
|  | Blu Classic Tobacco | 23.7 mg |  |

**Supplementary Material, Table S6. Main chemical compounds concentration of different disposable e-cigarette.**

| **Chemical** | **Products** | **Concentration** | **Ref** |
| --- | --- | --- | --- |
| (3Z)-3-Hexen-1-ol | JUUL (Cool Mint) | 5 μg/mL | (Omaiye et al., 2022b) |
|  | JUUL (Mint) | 5 μg/mL |  |
| 3-Hexen-1-ol | Puff Bar—Grape | 2.00 × 10⁻⁵ μg/kg | (Lin et al., 2022) |
|  | Air Bar (Watermelon Ice) | 2.00 × 10⁻⁵ μg/kg |  |
| α-Terpineol | Puff Bar—Grape | 2.00 × 10⁻⁵ μg/kg |  |
| γ-Decalactone | Air Bar (Watermelon Ice) | 5.60 × 10⁻⁴ μg/kg |  |
| Benzoic acid | Air Bar (Watermelon Ice) | 3.82 μg/kg |  |
|  | Puff Bar—Grape | 3.87 × 10⁻¹ μg/kg |  |
| Benzyl alcohol | Air Bar (Watermelon Ice) | 2.15 × 10⁻³ μg/kg |  |
|  | Elfbar (US) | 0.8 ± 0.7 mg/mL | (Leigh et al., 2025) |
|  | Elfbar (UK) | 0.9 ± 0.5 mg/mL |  |
| Caffeine | JUUL (Classic Menthol) | 12.7 μg/mL | (Omaiye et al., 2022b) |
| Ethyl maltol | Air Bar (Watermelon Ice) | 1.85 × 10⁻² μg/kg | (Lin et al., 2022) |
|  | Puff Bar—Grape | 2.15 × 10⁻² μg/kg |  |
|  | JUUL (Classic Menthol) | 8.3 μg/mL | (Omaiye et al., 2022b) |
|  | JUUL (Menthol) | 7.7 μg/mL |  |
|  | Elfbar (US) | 2.6 ± 1.5 mg/mL | (Leigh et al., 2025) |
|  | Elfbar (UK) | 2.7 ± 0.8 mg/mL |  |
| Flavor chemicals | Puff devices ranged from (Cucumber) to (Tobacco) | ranged from 0.7 (Cucumber) to 34.3 (Tobacco) mg/mL | (Omaiye et al., 2022a) |
| Formaldehyde | BLOW Disposable Vape Stix (Blueberry Raspberry) | Presence | (Ruggiero et al., 2022) |
|  | BLOW Disposable Vape Stix (Grape) | Non-presence |  |
|  | BLOW Disposable Vape Stix (Mango) | Presence |  |
|  | Minnesota Legit Drag (Below Zero) | Non-presence |  |
|  | Minnesota Legit Drag (Purple Rain) | Non-presence |  |
| H₂O₂ | 5% nicotine Hyde American Tobacco flavored bar | 10.43–10.72 μM | (Yogeswaran et al., 2021) |
|  | 5% nicotine Hype Bar Cool Melon bar | 9.44–9.76 μM |  |
|  | 5% Puff Bar Blue Razz | 8.15–9.11 μM |  |
|  | SOL: Spearmint (5% nicotine) | 8.78–9.25 μM |  |
|  | SMOQ: Pink Lemonade (5% nicotine) | 15.32–15.63 μM |  |
|  | Strawberries and Cream (5% nicotine) | 8.11–8.39 μM |  |
|  | 0% nicotine-containing Blue Razz bars | 5.68–5.82 μM |  |
|  | 0% nicotine Bolt: Orange Pop bar | 4.39–5.00 μM |  |
|  | 0% Bolt: Melon Ice Cream bar | 3.61–3.73 μM |  |
| Isophorone | Mi-Salt | ≤1 μg/g | (Tehrani et al., 2021) |
|  | Vuse | ≤1 μg/g |  |
| Melonal | Air Bar (Watermelon Ice) | 1.10 × 10−4 μg/kg | (Lin et al., 2022) |
| Menthol | Switch Blue Razz | 1.96 ± 0.08 mg/mL | (Jenkins et al., 2024b) |
|  | Switch Cucumber Lime | 2.5 ± 0.2 mg/mL |  |
|  | Switch Grape | 3.2 ± 0.1 mg/mL |  |
|  | Switch POG | 2.71 ± 0.03 mg/mL |  |
|  | HQD Blueberry | 0.316 ± 0.009 mg/mL |  |
|  | HQD Grapey | 0.287 ± 0.002 mg/mL |  |
|  | IGET Cool Mint | 4.0 ± 0.3 mg/mL |  |
|  | Elfbar Peach Ice | 0.3 mg/mL | (Leigh et al., 2025) |
|  | IGET Cool Mint | 4.0 ± 0.3 mg/mL | (Jenkins et al., 2024a) |
|  | Puff bar Cool Mint | 620 ± 50 μg/g | (Jabba et al., 2022) |
|  | Puff bar Café Latte | 1500 ± 50 μg/g |  |
|  | Puff bar Lush Ice | 2250 ± 190 μg/g |  |
|  | Puff bar Menthol | 3680 ± 550 μg/g |  |
|  | Puff bar Blueberry Ice | 2300 ± 40 μg/g |  |
|  | Puff bar OMG | 1330 ± 40 μg/g |  |
|  | Puff bar Banana Ice | 2040 ± 110 μg/g |  |
|  | Loon Maxd (Blue Lightning) | 1.2 μg/mg | (Erythropel et al., 2025) |
|  | SpreeBar (Sweet Spearmint) | 3.5 μg/mg |  |
|  | Crave clear 5% | 2.7 mg/g | (Minetti et al., 2024) |
|  | Crave ultra clear 5% | 6.6 mg/g |  |
|  | Crave+ kik new clear 5% | 6.8 mg/g |  |
|  | Elfbar clear 5% | 3.4 mg/g |  |
|  | Esco bars clear 5% | 6.6 mg/g |  |
|  | Flum pebble clear 5% | 4.0 mg/g |  |
|  | Flum float clear 5% | 4.0 mg/g |  |
|  | Fogger ultra clear 5% | 4.5 mg/g |  |
|  | Hitt infinite clear 5% | 1.6 mg/g |  |
|  | Hotbox clear 5% | 4.2 mg/g |  |
|  | Uoy bar clear 5% | 1.4 mg/g |  |
|  | Luto clear 2.8% | 5.1 mg/g |  |
|  | Luto smooth clear 5% | 0.5 mg/g |  |
|  | Pacha syn clear 5% | 2.0 mg/g |  |
|  | Podjuice Hyde IQ clear 5% | 9.2 mg/g |  |
|  | Puffbar clear 5% | 0.6 mg/g |  |
|  | Space max pro 5% | 0.8 mg/g |  |
|  | Vozol clear 5% | 4.1 mg/g |  |
| Methyl anthranilate | Puff bar—Grape | 3.00 × 10−5 μg/kg | (Lin et al., 2022) |
| Methyl dihydrojasmonate | Air Bar (Watermelon Ice) | 5.00 × 10−5 μg/kg |  |
| Perillartine | Puff Bar—Grape | <1.00 × 10−5 μg/kg |  |
| Total CCs | Ezzy Oval (Berry cool) | 138.53 (45.64) μg | (Talih et al., 2022) |
|  | Ezzy Oval (Mango lychee) | 77.74 (9.74) μg |  |
|  | Hyde (Cherry lemonade) | 35.37 (2.17) μg |  |
|  | Puff Bar (Banana ice) | 36.55 (1.43) μg |  |
|  | SEA (Mint) | 27.88 (7.98) μg |  |
| TPM | Ezzy Oval (Berry cool) | 108.0 (13.4) mg | (Talih et al., 2022) |
|  | Ezzy Oval (Mango lychee) | 99.7 (10.4) mg |  |
|  | Hyde (Cherry lemonade) | 65.1 (6.6) mg |  |
|  | Puff Bar (Banana ice) | 125.4 (4.6) mg |  |
|  | SEA (Mint) | 45.6 (12.8) mg |  |
|  | Disposable vapes | 81.7 (2.0) mg | (Soule et al., 2023b) |
|  | Rebuildable/box mod devices | 640.7 (279.5) mg |  |
|  | Pod mod devices | 88.2 (47.9) mg |  |
|  | Blankz | 0.200 mg/ml | (Hensel et al., 2023) |
| Tributyl O-acetylcitrate | Mi-Salt (Smok) aerosol | 20 μg/g | (Tehrani et al., 2021) |
| Triethyl citrate | Air Bar (Watermelon Ice) | 4.82 × 10−3 μg/kg | (Lin et al., 2022) |
|  | Puff Bar—Grape | 1.15 × 10−2 μg/kg |  |
| Vanillin | Blu | > 400 μg/g | (Tehrani et al., 2021) |
|  | Vuse aerosol | 90 μg/g |  |
|  | Vuse e-liquids | 50 μg/g |  |
|  | Mi-Salt | ≤1 μg/g |  |
|  | Air Bar (Watermelon Ice) | 3.20 × 10−4 μg/kg | (Lin et al., 2022) |
|  | Elfbar (US) | 1.4 ± 0.8 mg/mL | (Leigh et al., 2025) |
| WS-23 | Puff Plus (Cool Mint) | 45.1 mg/mL | (Omaiye et al., 2022a) |
|  | Puff Plus (Cucumber) | 5.1 mg/mL |  |
|  | Puff Plus (Menthol) | 4.3 mg/mL |  |
|  | Puff Plus (Tobacco) | 0.8 mg/mL |  |
|  | Switch Blue Razz | 11.5 ± 0.3 mg/mL | (Jenkins et al., 2024b) |
|  | Switch Cucumber Lime | 11.8 ± 0.4 mg/mL |  |
|  | Switch Grape | 11.4 ± 0.4 mg/mL |  |
|  | Switch POG | 11.2 ± 0.1 mg/mL |  |
|  | Trip Jungle Juice | 13.45 ± 0.07 mg/mL |  |
|  | Xtra Mango Tango | 13.0 ± 0.2 mg/mL |  |
|  | Gunnpod Lychee Ice | 32.7 ± 0.2 mg/mL |  |
|  | HQD Curve Plus Lychee Ice | 18.9 ± 0.6 mg/mL |  |
|  | HQD Blueberry | 19.6 ± 0.5 mg/mL |  |
|  | HQD Grapey | 13.0 ± 0.2 mg/mL |  |
|  | HQD Lemon Cake | 15.6 ± 0.5 mg/mL |  |
|  | HQD Strawberry | 16.2 ± 0.8 mg/mL |  |
|  | IGET Apple | 23.9 ± 0.8 mg/mL |  |
|  | IGET Bubble Gum | 18.5 ± 0.5 mg/mL |  |
|  | IGET Cool Mint | 32 ± 2 mg/mL |  |
|  | IGET Grape | 15.3 ± 0.2 mg/mL |  |
|  | IGET Grapefruit | 13.4 ± 0.6 mg/mL |  |
|  | IGET Guava Ice | 16.0 ± 0.4 mg/mL |  |
|  | IGET Guava Ice | 17.3 ± 0.4 mg/mL |  |
|  | IGET Lychee Ice | 13.3 ± 0.5 mg/mL |  |
|  | IGET Mango | 22.3 ± 0.4 mg/mL |  |
|  | IGET Passion Fruit Mango | 30.4 ± 0.3 mg/mL |  |
|  | IGET Peach Ice | 24.2 ± 0.3 mg/mL |  |
|  | IGET Pineapple Ice | 14.5 ± 0.1 mg/mL |  |
|  | IGET Strawberry | 21.3 ± 0.2 mg/mL |  |
|  | Puff Bar Blue Razz | 12.6 ± 0.1 mg/mL |  |
|  | Puff Bar Cool Mint | 8.8 ± 0.3 mg/mL |  |
|  | Puff Bar Lychee Ice | 12.1 ± 0.1 mg/mL |  |
|  | Puff Bar Lychee Ice | 19.5 ± 0.3 mg/mL |  |
|  | Puff Bar OMG | 3.6 ± 0.2 mg/mL |  |
|  | Puff Bar Pomegranate | 10.5 ± 0.1 mg/mL |  |
|  | Vaporlax Iced Cola | 37 ± 2 mg/mL |  |
|  | Vaporlax Lush Ice | 4.5 ± 0.2 mg/mL |  |
|  | Vaporlax Lush Ice | 4.46 ± 0.05 mg/mL |  |
|  | Vaporlax Lush Ice | 5.4 ± 0.2 mg/mL |  |
|  | Vaporlax Mango Ice | 21.2 ± 0.4 mg/mL |  |
|  | Vaporlax Pink Lemonade | 16.4 ± 0.5 mg/mL |  |
|  | Elfbar (US) | 17.8 ± 1.5 mg/mL | (Leigh et al., 2025) |
|  | Elfbar (UK) | 12.9 ± 5.4 mg/mL |  |
|  | Switch Blue Razz | 11.5 ± 0.3 mg/mL | (Jenkins et al., 2024a) |
|  | Switch Cucumber Lime | 11.8 ± 0.4 mg/mL |  |
|  | Switch Grape | 11.4 ± 0.4 mg/mL |  |
|  | Switch POG | 11.2 ± 0.1 mg/mL |  |
|  | Trip Jungle Juice | 13.45 ± 0.07 mg/mL |  |
|  | Xtra Mango Tango | 13.0 ± 0.2 mg/mL |  |
|  | Gunpod Lychee Ice | 32.7 ± 0.2 mg/mL |  |
|  | HQD Curve Plus Lychee Ice | 18.9 ± 0.6 mg/mL |  |
|  | HQD Blueberry | 19.6 ± 0.5 mg/mL |  |
|  | HQD Grapey | 13.0 ± 0.2 mg/mL |  |
|  | HQD Lemon Cake | 15.6 ± 0.5 mg/mL |  |
|  | HQD Strawberry | 16.2 ± 0.8 mg/mL |  |
|  | IGET Apple | 23.9 ± 0.8 mg/mL |  |
|  | IGET Bubble Gum | 18.5 ± 0.5 mg/mL |  |
|  | IGET Cool Mint | 32 ± 2 mg/mL |  |
|  | IGET Grape | 15.3 ± 0.2 mg/mL |  |
|  | IGET Grapefruit | 13.4 ± 0.6 mg/mL |  |
|  | IGET Guava Ice | 16.0 ± 0.4 mg/mL |  |
|  | IGET Guava Ice | 17.3 ± 0.4 mg/mL |  |
|  | IGET Lychee Ice | 13.3 ± 0.5 mg/mL |  |
|  | IGET Mango | 22.3 ± 0.4 mg/mL |  |
|  | IGET Passion Fruit Mango | 30.4 ± 0.3 mg/mL |  |
|  | IGET Peach Ice | 24.2 ± 0.3 mg/mL |  |
|  | IGET Pineapple Ice | 14.5 ± 0.1 mg/mL |  |
|  | IGET Strawberry | 21.3 ± 0.2 mg/mL |  |
|  | Puff Bar Blue Razz | 12.6 ± 0.1 mg/mL |  |
|  | Puff Bar Cool Mint | 8.8 ± 0.3 mg/mL |  |
|  | Puff Bar Lychee Ice | 12.1 ± 0.1 mg/mL |  |
|  | Puff Bar Lychee Ice | 19.5 ± 0.3 mg/mL |  |
|  | Puff Bar O.M.G | 3.6 ± 0.2 mg/mL |  |
|  | Puff Bar Pomegranate | 10.5 ± 0.1 mg/mL |  |
|  | Vaporax Iced Cola | 37 ± 2 mg/mL |  |
|  | Vaporax Lush Ice | 4.5 ± 0.2 mg/mL |  |
|  | Vaporax Lush Ice | 4.46 ± 0.05 mg/mL |  |
|  | Vaporax Lush Ice | 5.4 ± 0.2 mg/mL |  |
|  | Vaporax Mango Ice | 21.2 ± 0.4 mg/mL |  |
|  | Vaporax Pink Lemonade | 16.4 ± 0.5 mg/mL |  |
|  | Puff bar Cool Mint | 36,660 ± 530 μg/g | (Jabba et al., 2022) |
|  | Puff bar Café Latte | 2300 ± 30 μg/g |  |
|  | Puff bar Orange Ice | 22,950 ± 450 μg/g |  |
|  | Puff bar Lush Ice | 25,830 ± 450 μg/g |  |
|  | Puff bar Melon Ice | 8,810 ± 60 μg/g |  |
|  | Puff bar Cucumber | 3,940 ± 10 μg/g |  |
|  | Puff bar Lychee | 26,240 ± 240 μg/g |  |
|  | Puff bar Menthol | 1,610 ± 1,950 μg/g |  |
|  | Puff bar Blueberry Ice | 14,120 ± 210 μg/g |  |
|  | Puff bar Peach Ice | 11,540 ± 270 μg/g |  |
|  | Puff bar Banana Ice | 13,110 ± 50 μg/g |  |
|  | Loon Maxd (Blue Lightning) | 10.0 μg/mg | (Erythropel et al., 2025) |
|  | SpreeBar (Blood Orange Peach) | 4.44 μg/mg |  |
|  | SpreeBar (Blue Razz Ice) | 16.39 μg/mg |  |
|  | SpreeBar (Creamy Melon) | 5.74 μg/mg |  |
|  | SpreeBar (Pineapple Coconut) | 4.47 μg/mg |  |
|  | SpreeBar (Rainbow Fruit) | 4.34 μg/mg |  |
|  | SpreeBar (Strawberry Apple Melon) | 14.01 μg/mg |  |
|  | SpreeBar (Strawberry Mango) | 9.81 μg/mg |  |
|  | SpreeBar (Sweet Spearmint) | 16.18 μg/mg |  |
|  | SpreeBar (Watermelon Grape) | 4.81 μg/mg |  |
|  | Crave clear 5% | 28.4 mg/g | (Minetti et al., 2024) |
|  | Crave ultra clear 5% | 27.4 mg/g |  |
|  | Crave+ kik new clear 5% | 28.5 mg/g |  |
|  | Elfbar clear 5% | 30.4 mg/g |  |
|  | Esco bars clear 5% | 19.3 mg/g |  |
|  | Fire float clear 0% | 18.1 mg/g |  |
|  | Flum pebble clear 5% | 37.0 mg/g |  |
|  | Flum float clear 5% | 23.8 mg/g |  |
|  | Fogger ultra clear 5% | 20.1 mg/g |  |
|  | Hitt infinite clear 5% | 17.2 mg/g |  |
|  | Hotbox clear 5% | 29.4 mg/g |  |
|  | Uoy bar clear 5% | 27.5 mg/g |  |
|  | Luto clear 2.8% | 15.3 mg/g |  |
|  | Luto smooth clear 5% | 34.4 mg/g |  |
|  | Pacha syn clear 5% | 26.5 mg/g |  |
|  | Podjuice Hyde IQ clear 5% | 13.8 mg/g |  |
|  | Puffbar clear 5% | 15.6 mg/g |  |
|  | Space max pro 5% | 11.2 mg/g |  |
|  | Vozol clear 5% | 18.7 mg/g |  |
| WS-3 | IGET Bubble Gum | 1.21 ± 0.06 mg/mL | (Jenkins et al., 2024b) |
|  | IGET Passion Fruit Mango | 2.81 ± 0.03 mg/mL |  |
|  | Elfbar Strawberry Kiwi | 2.4 mg/mL | (Leigh et al., 2025) |
|  | IGET Bubble Gum | 1.21 ± 0.06 mg/mL | (Jenkins et al., 2024a) |
|  | IGET Passion Fruit Mango | 2.81 ± 0.03 mg/mL |  |
|  | Puff bar Cool Mint | 10,770 ± 210 μg/g | (Jabba et al., 2022) |
|  | Puff bar Orange Ice | 1,750 ± 20 μg/g |  |
|  | Puff bar Cucumber | 3,770 ± 20 μg/g |  |
|  | Puff bar Menthol | 3,540 ± 30 μg/g |  |
|  | Puff bar Blueberry Ice | 1,880 ± 20 μg/g |  |
|  | Loon Maxd (Blue Lightning) | 4.4 μg/mg | (Erythropel et al., 2025) |
|  | Crave ultra clear 5% | 6.4 mg/g | (Minetti et al., 2024) |
|  | Crave+ kik new clear 5% | 6.4 mg/g |  |
|  | Flum pebble clear 5% | 13.3 mg/g |  |
|  | Flum float clear 5% | 25.2 mg/g |  |
|  | Hotbox clear 5% | 13.6 mg/g |  |
|  | Luto smooth clear 5% | 4.6 mg/g |  |
|  | Space max pro 5% | 1.8 mg/g |  |
| Chromium | Air Bar (Watermelon Ice) | 4.58 × 10⁻³ μg/kg | (Lin et al., 2022) |
|  | Puff Bar—Grape | 1.78 × 10⁻³ μg/kg |  |
| Nickel | Air Bar (Watermelon Ice) | 3.91 × 10⁻³ μg/kg |  |
|  | Puff Bar—Grape | 2.04 × 10⁻³ μg/kg |  |
| Manganese | Air Bar (Watermelon Ice) | 1.07 × 10⁻³ μg/kg |  |
|  | Puff Bar—Grape | 5.10 × 10⁻³ μg/kg |  |
| Lead | Air Bar (Watermelon Ice) | 1.32 × 10⁻³ μg/kg |  |
|  | Puff Bar—Grape | 2.10 × 10⁻³ μg/kg |  |
| Aluminum | Air Bar (Watermelon Ice) | 2.38 × 10⁻² μg/kg |  |
|  | Puff Bar—Grape | 9.03 × 10⁻² μg/kg |  |
| Zinc | Air Bar (Watermelon Ice) | 2.50 × 10⁻² μg/kg |  |
|  | Puff Bar—Grape | 1.43 × 10⁻² μg/kg |  |

**References:**

2022. Nicotine Uptake and Abuse Liability Assessments of 5 Blu Disposable Electronic Cigarettes in Comparison to a Combustible Cigarette.

2024. Compensatory Behavior of E-cig Users in Response to Reduced Nicotine E-liquids.

Abadi, M. H., Lipperman-Kreda, S., Shamblen, S. R., Thompson, K., Grube, J. W., Leventhal, A. M., Luseno, W. & Aramburu, C. 2021. The impact of flavored ENDS use among adolescents on daily use occasions and number of puffs, and next day intentions and willingness to vape. *Addictive Behaviors,* 114**,** 106773.

Aherrera, A., Lin, J. J., Chen, R., Tehrani, M., Schultze, A., Borole, A., Tanda, S., Goessler, W. & Rule, A. M. 2023. Metal Concentrations in E-Cigarette Aerosol Samples: A Comparison by Device Type and Flavor. *Environ Health Perspect,* 131**,** 127004.

Ahn, S., Ma, S. & Shang, C. 2025. Where Do E-cigarette Users Buy Their E-cigarette Products? Results from a Nationally Representative Sample of Adult E-cigarette Users in the United States. *Nicotine & tobacco research : official journal of the Society for Research on Nicotine and Tobacco*.

Alam, A., Henriksen, L., Johnson, T. O., Prochaska, J. J. & Schleicher, N. 2025. Early assessment of compliance with California's flavoured tobacco sales prohibition: evidence from vape shops. *Tobacco control*.

Alam El Din, S. M., Iyengar, S., Aherrera, A. D., Quarles, C. D., Mihalic, J., Bressler, J. & Rule, A. M. 2025. Detection of Metal-Containing Particles in FDA-Authorized and New Disposable Electronic Cigarette Aerosols. *Environmental Science and Technology,* 59**,** 24707-24716.

Alcantara, C., Chaparro, L. & Zagury, G. J. 2023. Occurrence of metals in e-cigarette liquids: Influence of coils on metal leaching and exposure assessment. *HELIYON,* 9.

Alemu, S. M., Mons, U., Schaller, K., Baltaci, E. & Hoe, C. 2025. Spatial distribution of e-cigarette vending machines: a case study from Germany. *Tobacco control*.

Ali, F. R. M., Diaz, M. C., Armour, B. S., Crane, E., Tynan, M. A. & Marynak, K. L. 2025. Trends in U.S. E-cigarette Sales Measured in Milligrams of Nicotine, 2019-2024. *American Journal of Preventive Medicine,* 68**,** 1173-1178.

Ali, F. R. M., Seaman, E. L., Crane, E., Schillo, B. & King, B. A. 2023. Trends in US E-cigarette Sales and Prices by Nicotine Strength, Overall and by Product and Flavor Type, 2017-2022. *Nicotine Tob Res,* 25**,** 1052-1056.

Ali, F. R. M., Seaman, E. L., Diaz, M. C., Ajose, J. & King, B. A. 2024. Trends in unit sales of cooling flavoured e-cigarettes, USA, 2017-2021. *Tobacco control,* 33**,** 147-153.

AlMuraikhi, H. M., Chokor, F. A. Z. & Al-Hamdani, M. 2025. Correlates of perceived social unacceptability of vaping among regular e-cigarette users: a cross-sectional study of a sample of Middle Eastern countries. *Frontiers in public health,* 13**,** 1620863.

Alowedi, S., Wang, P., Wang, Y., Sharma, E. F. & Sharma, S. 2023. Immediate effects of e-cigarette use on systemic cardiovascular outcomes and retinal perfusion as measured by OCT angiography: a pilot study. *Investigative Ophthalmology & Visual Science,* 64**,** 3035-3035.

Appleton, S., Cyrus-Miller, H., Seltzer, R., Gilligan, K. & McKinney, W. 2022. Market survey of disposable e-cigarette nicotine content and e-liquid volume. *BMC Public Health,* 22**,** 1760.

Avila, J. C., Maglalang, D. D., Nollen, N. L., Lee, S. C., Suh, R., Malone, M., Binjrajka, U. & Ahluwalia, J. S. 2024. Using pod based e-cigarettes and nicotine pouches to reduce harm for adults with low socioeconomic status who smoke: a pilot randomized controlled trial. *Nicotine & tobacco research,* 26**,** 1150.

Azagba, S., Ebling, T. & Korkmaz, A. 2025. Disposable e-cigarette use: Factors, frequency and cigarette smoking among United States high school students. *Addiction (Abingdon, England),* 120**,** 423-431.

Bansal-Travers, M., Rivard, C., Delnevo, C. D., Gross, A., Anesetti-Rothermel, A., Merson, B., Xiao, H., Cheng, Y. C., Creamer, M. R., Kimmel, H. L., Stanton, C. A., Sharma, E., Taylor, K., Lauten, K., Goniewicz, M. & Hyland, A. 2025. Flavor and Device Choices Among People Who Use ENDS: Results From the PATH Study. *American Journal of Preventive Medicine,* 68**,** 236-244.

Beard, J. M., Collom, C., Liu, J. Y., Obiako, P., Strongin, R. M., Zavala, J. & Sayes, C. M. 2024. In vitro toxicity and chemical analysis of e-cigarette aerosol produced amid dry hitting. *Toxicology,* 506**,** 153865.

Bejarano, G., Bluestein, M. A., Tackett, A. P., Duano, J. C., Rawls, S. G., Ahluwalia, J. S., Vandewater, E. A. & Hebert, E. T. 2024. Factors Associated With Successful E-Cigarette Cessation Among a Convenience Sample of Adult Users. *Substance use & misuse,* 59**,** 1126-1132.

Belderson, P., Ward, E., Pope, I. & Notley, C. 2024. Selecting an e-cigarette for use in smoking cessation interventions and healthcare services: findings from patient and public consultation for the COSTED trial. *BMJ open,* 14**,** e078677.

Bertrand, A., Diaz, M. C., Minter, T., Goyette, R., Schillo, B. A. & Kreslake, J. M. 2026. Clearly flavoured: rising popularity of 'clear' flavoured e-cigarettes and what online descriptions reveal. *Tobacco control*.

Boakye, E., Dzaye, O., Erhabor, J., Osuji, N., Obisesan, O., Osei, A. D., Bhatnagar, A., Robertson, R. M. & Blaha, M. J. 2022. Impact of the Food and Drug Administration enforcement policy on flavored e-cigarettes on the online popularity of disposable e-cigarettes: analyses of Google search query data. *BMC Public Health,* 22**,** 1937.

Bold, K. W., Kong, G., Morean, M., Gueorguieva, R., Camenga, D. R., Simon, P., Davis, D. R., Jackson, A. & Krishnan-Sarin, S. 2021. Trends in various e-cigarette devices used by high school adolescents from 2017-2019. *DRUG AND ALCOHOL DEPENDENCE,* 219.

Boots, B., Green, D. S., Wright, A. C. M., Olah-Kovacs, B. & Tovey, L. 2024. Ecotoxicological effects of leachate from e-cigarettes and e-liquid on the performance of perennial ryegrass (Lolium perenne). *Environmental Pollution,* 348**,** 123888.

Borowiecki, M., Kim, Y. & Emery, S. 2024. A Patchy Prohibition: Product and Flavor Substitution After the Food and Drug Administration's Prioritized Enforcement Policy on Flavored E-cigarettes. *Nicotine and Tobacco Research,* 26**,** 527-535.

Brouwer, A. F., Jeon, J., Jimenez-Mendoza, E., Land, S. R., Holford, T. R., Friedman, A. S., Tam, J., Mistry, R., Levy, D. T. & Meza, R. 2025. Changing patterns of cigarette and ENDS transitions in the USA: a multistate transition analysis of adults in the PATH Study in 2017-2019 vs 2019-2021. *Tobacco control,* 34**,** 783-790.

Brown, J., Colby, S. & Walker, N. 2025. Introducing a special issue of rapid research on disposable e-cigarettes. *Addiction (Abingdon, England),* 120**,** 398-401.

Cantrell, J., Huang, J., Greenberg, M., Willett, J., Hair, E. & Vallone, D. 2021. History and Current Trends in the Electronic Cigarette Retail Marketplace in the United States: 2010-2016. *Nicotine and Tobacco Research,* 22**,** 843-847.

Cao, Y., Hu, Z., Li, J., Chen, X., Xiong, Y., Zheng, F., Zhang, J., Zhang, L. & Liu, X. 2025. Assessing Nicotine Pharmacokinetics of New-Generation Tobacco Products and Conventional Cigarettes: A Systematic Review and Meta-analysis. *Nicotine and Tobacco Research,* 27**,** 783-793.

Chaffee, B. W., Halpern-Felsher, B., Croker, J. A., Werts, M., Couch, E. T. & Cheng, J. 2022. Preferences, use, and perceived access to flavored e-cigarettes among United States adolescents and young adults. *Drug Alcohol Depend Rep,* 3.

Chen-Sankey, J., Ganz, O., Seidenberg, A. & Choi, K. 2023. Effect of a 'tobacco-free nicotine' claim on intentions and perceptions of Puff Bar e-cigarette use among non-tobacco-using young adults. *Tob Control,* 32**,** 501-504.

Chen-Sankey, J., Weiger, C., La Capria, K., Vassey, J., Jeong, M., Phan, L., Unger, J. B., Allem, J. P., Berg, C. J. & Choi, K. 2025. Young adults' visual attention to features of social media marketing for disposable e-cigarettes and associated perceptions. *Addiction (Abingdon, England),* 120**,** 439-451.

Chen, D. T. H., Girvalaki, C. & Filippidis, F. T. 2024. Disposable e-cigarette use and associated factors in US middle and high school students, 2021-2022. *Tobacco Induced Diseases,* 22.

Christen, S. E., Hermann, L., Bekka, E., Vonwyl, C., Hammann, F., van der Velpen, V., Eap, C. B., Benowitz, N. L., Haschke, M. & Liakoni, E. 2024. Pharmacokinetics and pharmacodynamics of inhaled nicotine salt and free-base using an e-cigarette: a randomized crossover study. *Nicotine & tobacco research,* 26**,** 1313.

Cooper, S. Y., Olszewski, N. A., Tetteh-Quarshie, S., Hill, S. P., Ghodsi, S., Gonzalez-Castro, A., Willis, C. V. & Henderson, B. J. 2024. The Impact of High or Low Doses of Nicotine in a Mouse Model of Vapor Self-Administration. *Nicotine and Tobacco Research,* 26**,** 316-323.

Craft, S., Sunderland, P., Millea, M. F., Pudney, C. R., Sutcliffe, O. B. & Freeman, T. P. 2025. Detection and quantification of synthetic cannabinoids in seven illicitly sourced disposable vapes submitted by an individual presenting to a UK drug and alcohol service. *Addiction (Abingdon, England),* 120**,** 549-554.

Crespi, E., Hardesty, J. J., Nian, Q. & Cohen, J. E. 2024. Decisions of the FDA on premarket tobacco product applications: Changes in the number of unique devices and liquids used by US adults who frequently use electronic nicotine delivery systems, 2020-2023. *Tobacco Induced Diseases,* 22.

Crespi, E., Hardesty, J. J., Nian, Q. & Cohen, J. E. 2025. Differences in E-Cigarette Use Behaviors and Device and Liquid Characteristics of U.S. Adults by Sexual and Gender Identity. *Nicotine and Tobacco Research,* 27**,** 1473-1478.

Crespi, E., Hardesty, J. J., Nian, Q., Sinamo, J., Welding, K., Cohen, J. E. & David Kennedy, R. 2023. Device and liquid characteristics used with sweet, menthol/mint, and tobacco ENDS liquid flavors: The population-based VAPER study. *Addict Behav,* 144**,** 107727.

Cruz-Jimenez, L., Vidana-Perez, D. & Thrasher, J. F. 2025. Evaluating strategies to reduce effects of e-cigarette social media posts on risk perceptions susceptibility among youth. *Tobacco induced diseases,* 23**,** 247.

Cui, T., Lu, R., Liu, Q., Jiang, X., Li, Y. & Pan, S. 2023. PM1 exposure and spatial transmission of nicotine from the simulated second-hand vapor of pod-based electronic cigarettes. *Sci Total Environ,* 897**,** 165355.

Dai, H. & Hao, J. 2022. Online popularity of JUUL and Puff Bars in the USA: 2019-2020. *Tob Control,* 31**,** 7-10.

Diaz, M. C., Silver, N. A., Bertrand, A. & Schillo, B. A. 2025. Bigger, stronger and cheaper: growth in e-cigarette market driven by disposable devices with more e-liquid, higher nicotine concentration and declining prices. *Tobacco control,* 34**,** 65-70.

Do, V. V., Shang, C., Huang, J., Islam, T., Pechacek, T. F. & Weaver, S. R. 2025. Volumetric choice experiment to estimate the impact of e-cigarette and heated tobacco product characteristics on substitution and complementary use among adults who smoke cigarettes and recently initiated e-cigarette use. *BMJ Open,* 15**,** e100073.

Donaldson, S. I., Beard, T., Dormanesh, A., Perez, C., Escobedo, P., Unger, J. B., Wipfli, H. L., Galimov, A. & Allem, J. P. 2025. Monitoring website marketing among leading e-cigarette brands and vendors in California: content analysis. *Tobacco control,* 34**,** 192-197.

Donaldson, S. I., Beard, T. A., Trinh, J., Jaladanki, S., Unger, J. B., Galimov, A., Wipfli, H. L. & Allem, J. P. 2024. Content Analysis of E-mail Marketing Communications Among Online E-cigarette Retailers. *Nicotine and Tobacco Research,* 26**,** 1248-1252.

Dong, Z., Pang, Q., Ling, Q., He, C., Wu, G., Wei, S., Deng, W., Liu, C., Qian, J., Fu, Y., Hai, T., Chen, Z., Yun, L. & Rong, X. 2025. Heating Coil Corrosion by E-Liquid Containing Nicotine Lactate Salt and In Vitro and In Vivo Evaluation of Nickel Leachate in E-Liquid and E-Cigarette Aerosol. *Chemical Research in Toxicology,* 38**,** 1557-1563.

Donovan, E. M., Azadi, M., McKay, T., Aarvig, K. & Kreslake, J. 2025. Not-so-disposable e-cigarettes: Methods young people use to discard single-use e-cigarettes. *Addiction (Abingdon, England),* 120**,** 452-457.

Douglas, A. E., Childers, M. G., Felicione, N. J., Milstred, A. R. & Blank, M. D. 2024. Electronic Cigarette Device and Liquid Characteristics: Comparison of Self-Reports to User-Provided Pictures. *Substance use & misuse,* 59**,** 1990-1998.

Dziegiel, M., Maciejowska, A., Misiak, M. & Lisowska, K. A. 2025. Nicotinism vs. Glomerulopathies-Smoking as a Risk Factor for Primary Glomerulopathies. *Antioxidants (Basel, Switzerland),* 14.

East, K., Vu, G., Sun, T., D'Mello, K., Perman-Howe, P. R., Taylor, E., Nottage, M., Brose, L. S., Robson, D. & McNeill, A. 2025. Harm perceptions across vaping product features: An on-line cross-sectional survey of adults who smoke and/or vape in the United Kingdom. *Addiction (Abingdon, England),* 120**,** 524-538.

Easwaran, M., Maria, C. S., Martinez, J. D., Hung, B., Yu, X., Soo, J., Kimura, A., Gross, E. R. & Erickson-DiRenzo, E. 2024. Effects of Short-term Electronic(e)-Cigarette Aerosol Exposure in the Mouse Larynx. *Laryngoscope,* 134**,** 1316-1326.

Edmiston, J. S., Webb, K. M., Wang, J., Oliveri, D., Liang, Q. & Sarkar, M. 2022. Biomarkers of Exposure and Biomarkers of Potential Harm in Adult Smokers Who Switch to e-Vapor Products Relative to Cigarette Smoking in a 24-week, Randomized, Clinical Trial. *Nicotine Tob Res,* 24**,** 1047-1054.

Erinoso, O., Osibogun, O., Li, W. & Kalan, M. E. 2024. Longitudinal examination of ENDS characteristics, flavors, and nicotine content for cigarette cessation: Findings from PATH waves 5-6. *Addictive Behaviors,* 157**,** 108097.

Erythropel, H. C., Jabba, S. V., Mellinghoff, R. A., Garcia-Gallet, V., Silinski, P., Zimmerman, J. B. & Jordt, S. E. 2025. Prevalence of Potent Artificial Sweetener Neotame in Popular US-Marketed Disposable Electronic Cigarettes. *Research square*.

Falarowski, C., Pieper, E., Rabenstein, A., Mallock-Ohnesorg, N., Burgmann, N., Franzen, K., Gertzen, M., Koller, G., Nowak, D., Rahofer, A., Rieder, B., de Oliveira Pinto Kise, G. R., Schulz, T., Strohmeyer, E., Laux, P., Luch, A. & Rüther, T. 2025. Disposable e-cigarettes and their nicotine delivery, usage pattern, and subjective effects in occasionally smoking adults. *Scientific reports,* 15**,** 16270.

Fan, J., Brown, J., Cox, S., Shahab, L. & Jackson, S. E. 2025. Level of support for proposed vaping policies: an online survey of UK vapers, 2024. *International Journal of Drug Policy,* 142**,** 104839.

Farra, Y. M., Matz, J., Wilker, H., Kim, H., Rivera, C., Vlahos, J., Ramkhelawon, B., Oakes, J. M. & Bellini, C. 2024. Chronic Pod-Mod E-Cigarette Aerosol Exposure Induces Aortic Dysfunction in Hypercholesterolemic Mice: Role of Oxidative Stress and Inflammation. *bioRxiv*.

Fearon, I. M., Gilligan, K., Seltzer, R. G. N. & McKinney, W. 2022. A randomised, crossover, clinical study to assess nicotine pharmacokinetics and subjective effects of the BIDI((R)) stick ENDS compared with combustible cigarettes and a comparator ENDS in adult smokers. *Harm Reduct J,* 19**,** 57.

Ford, A., MacKintosh, A. M., Morgan, A., Jones, D., Moodie, C., Hunt, K. & Angus, K. 2025. A Content Analysis of Nicotine Descriptors on the Front of Vape Packaging in the United Kingdom. *Nicotine and Tobacco Research,* 27**,** 152-156.

Frie, J. A., McCunn, P., Eed, A., Hassan, A., Luciani, K. R., Chen, C., Tyndale, R. F. & Khokhar, J. Y. 2024. Factors influencing JUUL e-cigarette nicotine vapour-induced reward, withdrawal, pharmacokinetics and brain connectivity in rats: sex matters. *Neuropsychopharmacology,* 49**,** 782-795.

Gaiha, S. M., Lempert, L. K., McKelvey, K. & Halpern-Felsher, B. 2021. 109. Adolescent E-Cigarette Use and Access Before and During the COVID-19 Pandemic. *Journal of Adolescent Health,* 68**,** S57.

Gaiha, S. M., Lempert, L. K., McKelvey, K. & Halpern-Felsher, B. 2022. E-cigarette devices, brands, and flavors attract youth: Informing FDA's policies and priorities to close critical gaps. *Addict Behav,* 126**,** 107179.

Gale, A., Kelly, M., Belfield, J. B., Williams, N., Fisher, M., Guarraci, F. A. & Stokes, J. A. 2024. Prepubescent Electronic Cigarette Exposure Affects Sexual Motivation and Puberty in Female but Not Male Long-Evans Rats. *Nicotine and Tobacco Research,* 26**,** 1463-1471.

Galimov, A., Albers, L., Rahman, T., Vassey, J., Kirkpatrick, M. G. & Unger, J. B. 2024. Assessing the public discourse on Twitter: Reactions to the JUUL e-cigarettes ban in the United States. *Tobacco Induced Diseases,* 22.

Galimov, A., Leventhal, A., Meza, L., Unger, J. B., Huh, J., Baezconde-Garbanati, L. & Sussman, S. Y. 2021. Prevalence of disposable pod use and consumer preference for e-cigarette product characteristics among vape shop customers in Southern California: a cross-sectional study. *BMJ Open,* 11**,** e049604.

Gentzke, A. S., Wang, T. W., Cornelius, M., Park-Lee, E., Ren, C., Sawdey, M. D., Cullen, K. A., Loretan, C., Jamal, A. & Homa, D. M. 2022. Tobacco Product Use and Associated Factors Among Middle and High School Students - National Youth Tobacco Survey, United States, 2021. *MMWR SURVEILLANCE SUMMARIES,* 71**,** 1-32.

Glasser, A. M., Vojjala, M., Cantrell, J., Levy, D. T., Giovenco, D. P., Abrams, D. & Niaura, R. 2021. Patterns of E-cigarette Use and Subsequent Cigarette Smoking Cessation Over 2 Years (2013/2014-2015/2016) in the Population Assessment of Tobacco and Health Study. *NICOTINE & TOBACCO RESEARCH,* 23**,** 669-677.

Goicoechea, J. Z., Boughner, A., Lee, J. J. C., Mahajan, A., Yeo, K., Sproga, M., Russell, C., Coughlan, M., Selya, A., Caci, G., Caponnetto, P., Tomaselli, V. & Polosa, R. 2024. Respiratory symptoms among e-cigarette users without an established smoking history in the VERITAS cohort. *Scientific reports,* 14**,** 28549.

Goldenson, N. I., Shiffman, S., Sembower, M. A. & Black, R. A. 2024. Assessment of abuse liability and switching potential of menthol-flavored pod-based electronic nicotine delivery systems among US adults who smoke cigarettes. *Drug and alcohol dependence,* 258**,** 111279.

Gravely, S., Meng, G., Hammond, D., Reid, J. L., Seo, Y. S., Hyland, A., Cummings, K. M., Rivard, C., Fong, G. T. & Kasza, K. A. 2022. Electronic nicotine delivery systems (ENDS) flavours and devices used by adults before and after the 2020 US FDA ENDS enforcement priority: findings from the 2018 and 2020 US ITC Smoking and Vaping Surveys. *Tob Control,* 31**,** s167-s175.

Gravely, S., Smith, T. T., Toll, B. A., Ashley, D., Driezen, P., Levy, D. T., Quah, A. C. K., Fong, G. T. & Michael Cummings, K. 2024. Electronic nicotine delivery system flavors, devices, and brands used by adults in the United States who smoke and formerly smoked in 2022: Findings from the United States International Tobacco Control Four Country Smoking and Vaping Survey. *Preventive Medicine Reports,* 47**,** 102905.

Gravely, S., Sweanor, D., Driezen, P., Levy, D. T., Fong, G. T., Quah, A. C. K., Craig, L. V., Chung-Hall, J., Kaai, S. C. & Cummings, K. M. 2025. Use of nicotine vaping products during an attempt to quit smoking by Canadian adults who smoke or recently quit: findings from the 2022 Canada International Tobacco Control Four Country Smoking and Vaping Survey. *Utilisation de produits de vapotage contenant de la nicotine lors d'une tentative d'abandon du tabagisme par des adultes canadiens fumeurs ou ayant recemment cesse de fumer : resultats concernant le Canada tires de l'Enquete sur le tabagisme et le vapotage dans quatre pays menee en 2022 par le Projet international d'evaluation de la lutte antitabac.,* 45**,** 54-60.

Gravely, S., Yong, H. H., Reid, J. L., East, K. A., Liber, A. C., Michael Cummings, K., Quah, A. C. K., Fong, G. T. & Hammond, D. 2023. An examination of quitting smoking as a reason for vaping by the type of nicotine vaping device used most often among adults who smoke and vape: Findings from the Canada, England and the United States 2020 ITC Smoking and Vaping Survey. *Prev Med Rep,* 33**,** 102201.

Green, D. S., Boots, B., Olah-Kovacs, B. & Palma-Diogo, D. 2023. Disposable e-cigarettes and cigarette butts alter the physiology of an aquatic plant Lemna minor (Lemnaceae). *SCIENCE OF THE TOTAL ENVIRONMENT,* 892.

Green, D. S., Boots, B. & Tingey, T. 2024. Rapid, detrimental response of estuarine benthic macrofauna communities to pollution by littered cigarette filters and e-liquid. *Marine Pollution Bulletin,* 209**,** 117208.

Guerra Castillo, C., Hoeft, K. S., Couch, E. T., Urata, J., Halpern-Felsher, B. & Chaffee, B. W. 2024. Adolescents' Experiences and Perceptions of E-Cigarettes and Nicotine Addiction. *Substance use & misuse,* 59**,** 1981-1989.

Hammond, D. 2024. The rise of disposable e-cigarettes in England and implications for public health. *BMJ***,** q1508.

Hammond, D. & Reid, J. L. 2025. Trends in vaping and nicotine product use among youth in Canada, England and the USA between 2017 and 2022: evidence to inform policy. *Tobacco control,* 34**,** 115-118.

Hammond, D., Reid, J. L., Burkhalter, R. & East, K. 2025. Use of disposable e-cigarettes among youth who vape in Canada, England and the United States: Repeat cross-sectional surveys, 2017-2023. *Addiction (Abingdon, England),* 120**,** 405-413.

Han, D. H., Harlow, A. F., Feldstein Ewing, S. W., Audrain-McGovern, J. E., Unger, J. B., Sussman, S. Y., McConnell, R., Barrington-Trimis, J. L. & Leventhal, A. M. 2024. Disposable E-Cigarette Use and Subsequent Use Patterns in Adolescents and Young Adults. *Pediatrics,* 153**,** e2023063430.

Han, D. H., Lee, S. H. & Seo, D. C. 2022. Association of preferred flavorings and device type with box or pack purchase behavior of electronic nicotine delivery systems in the United States. *J Public Health Policy,* 43**,** 65-76.

Harris, T. 2025. Physical and Chemical Characterization of Aerosols Produced from Commercial Nicotine Salt-Based E-Liquids. *Chemical Research in Toxicology,* 38**,** 115-128.

Hensel, E. C., Sarles, S. E., Nuss, C. J., Terry, J. N., Polgampola Ralalage, C. R., DiFrancesco, A. G., Walton, K., Eddingsaas, N. C. & Robinson, R. J. 2023. Effect of third-party components on emissions from a pod style electronic cigarette. *Toxicol Sci,* 197**,** 104-109.

Hensel, E. C., Sarles, S. E., Nuss, C. J., Terry, J. N., Ralalage, C. R. P., DiFrancesco, A. G., Walton, K., Eddingsaas, N. C. & Robinson, R. J. 2024. Effect of third-party components on emissions from a pod style electronic cigarette. *Toxicological Sciences,* 197**,** 104–109.

Higgins, S. T., Sigmon, S. C., Tidey, J. W., Heil, S. H., Gaalema, D. E., Lee, D. C., DeSarno, M. J., Klemperer, E. M., Menson, K. E., Cioe, P. A., Plucinski, S., Wiley, R. C. & Orr, E. 2024. Reduced Nicotine Cigarettes and E-Cigarettes in High-Risk Populations: 3 Randomized Clinical Trials. *JAMA network open,* 7**,** e2431731.

Holt, A. K., Buckmire, M. G., Moss, K. S., Meekins, J., Outhous, A. E., Reveil, L., Goden, A. B., Hoetger, C., Eversole, A., Poklis, J. L., Soule, E. K., Cobb, C. O. & Peace, M. R. 2024a. A multi-year characterization of confiscated vaping products from Virginia school youth. *Journal of Analytical Toxicology,* 48**,** 606-615.

Holt, A. K., Rudy, A. K., Sawyer, A. N., Poklis, J. L., Breland, A. B. & Peace, M. R. 2023. Survey of U.S. Residents and Their Usage of Electronic Cigarettes with Drugs Other Than Nicotine. *Journal of psychoactive drugs***,** 1-10.

Holt, A. K., Rudy, A. K., Sawyer, A. N., Poklis, J. L., Breland, A. B. & Peace, M. R. 2024b. Survey of U.S. Residents and Their Usage of Electronic Cigarettes with Drugs Other Than Nicotine. *Journal of Psychoactive Drugs,* 56**,** 568-577.

Holt, L. J., Petrey, A. M., Bravo, A. J., Folivi, F., Conner, B. T., Looby, A., Prince, M. A., Schepis, T. S. & Yeung, E. W. 2025. E-cigarette Quit Attempts in Emerging Adults: Motivations for Attempts and Predictors of Cessation Barriers. *Nicotine and Tobacco Research,* 27**,** 1209-1217.

Huh, J., Galimov, A., Meza, L., Galstyan, E., Unger, J. B., Baezconde-Garbanati, L. & Sussman, S. 2024. Evaluating trends in recruitment challenges in vape shop research, e-cigarette product characteristics and use among shop customers from 2019 to 2023: A mixed-methods study. *Tobacco Induced Diseases,* 22.

Iacobucci, G. 2024. Disposable vapes: Paediatricians hail proposed ban. *BMJ (Clinical research ed.),* 384**,** q245.

Jabba, S. V., Erythropel, H. C., Torres, D. G., Delgado, L. A., Woodrow, J. G., Anastas, P. T., Zimmerman, J. B. & Jordt, S. E. 2022. Synthetic Cooling Agents in US-marketed E-cigarette Refill Liquids and Popular Disposable E-cigarettes: Chemical Analysis and Risk Assessment. *Nicotine Tob Res,* 24**,** 1037-1046.

Jackson, S. E., Brown, J., Shahab, L., Arnott, D., Bauld, L. & Cox, S. 2025a. Nicotine strength of e-liquids used by adult vapers in Great Britain: A population survey 2016 to 2024. *Addiction,* 120**,** 468-482.

Jackson, S. E., Cox, S., Shahab, L. & Brown, J. 2024a. Trends and patterns of dual use of combustible tobacco and e-cigarettes among adults in England: a population study, 2016-2024. *medRxiv*.

Jackson, S. E., Shahab, L., Tattan-Birch, H., Buss, V. & Brown, J. 2025b. Changes in vaping trends since the announcement of an impending ban on disposable vapes: A population study in Great Britain. *Addiction,* 120**,** 1876-1883.

Jackson, S. E., Tattan-Birch, H. & Brown, J. 2025c. Trends in where people buy their vaping products and differences by user and device characteristics: A population study in England, 2016-23. *Addiction (Abingdon, England),* 120**,** 539-548.

Jackson, S. E., Tattan-Birch, H., Shahab, L. & Brown, J. 2023. How has Expenditure on Nicotine Products Changed in a Fast-Evolving Marketplace? A Representative Population Survey in England, 2018-2022. *Nicotine Tob Res,* 25**,** 1585-1593.

Jackson, S. E., Tattan-Birch, H., Shahab, L. & Brown, J. 2024b. Trends in long term vaping among adults in England, 2013-23: population based study. *BMJ***,** e079016.

Jackson, S. E., Tattan-Birch, H., Shahab, L., Oldham, M., Kale, D., Brose, L. & Brown, J. 2024c. Who would be affected by a ban on disposable vapes? A population study in Great Britain. *Public Health,* 227**,** 291-298.

Jenkins, C., Morgan, J. & Kelso, C. 2023. Synthetic Cooling Agents in Australian-marketed E-cigarette Refill Liquids and Disposable E-cigarettes: Trends Follow the US Market. *Nicotine Tob Res*.

Jenkins, C., Morgan, J. & Kelso, C. 2024a. Synthetic Cooling Agents in Australian-Marketed E-cigarette Refill Liquids and Disposable E-cigarettes: Trends Follow the U.S. Market. *Nicotine and Tobacco Research,* 26**,** 380-384.

Jenkins, C., Morgan, J. & Kelso, C. 2024b. Synthetic Cooling Agents in Australian-Marketed E-cigarette Refill Liquids and Disposable E-cigarettes: Trends Follow the U.S. Market. *Nicotine Tob Res,* 26**,** 380-384.

Jenkins, C., Powrie, F., Kelso, C. & Morgan, J. 2025. Chemical Analysis and Flavor Distribution of Electronic Cigarettes in Australian Schools. *Nicotine and Tobacco Research,* 27**,** 997-1005.

Jenkins, C., Powrie, F., Morgan, J. & Kelso, C. 2024c. Labelling and composition of contraband electronic cigarettes: Analysis of products from Australia. *International Journal of Drug Policy,* 128**,** 104466.

Jongenelis, M. I. 2023. E-cigarette product preferences of Australian adolescent and adult users: a 2022 study. *BMC Public Health,* 23**,** 220.

Jongenelis, M. I., McCausland, K. & Bode, S. 2025. The impact of e-cigarette product characteristics on attitudes and use susceptibility: an experimental study. *Health promotion international,* 40.

Jordt, S. E., Silinski, P. & Berman, M. L. 2024. An Electronic Cigarette Pod System Delivering 6-methyl Nicotine, A Synthetic Nicotine Analog, Marketed in the United States as "PMTA Exempt". *American Journal of Respiratory and Critical Care Medicine,* 209.

Kapiamba, K. F., Chuang, H. Y., Hao, W., Lin, T. C., Chen, L. C., Huang, Y. W. & Wang, Y. 2025. Evaluating the Toxicity of Electronic Cigarette Aerosols for Firsthand and Secondhand Exposure Under Different Device Operating Conditions. *Chemical Research in Toxicology,* 38**,** 1344-1356.

Kasza, K. A., Hammond, D., Reid, J. L., Rivard, C. & Hyland, A. 2023. Youth Use of e-Cigarette Flavor and Device Combinations and Brands Before vs After FDA Enforcement. *JAMA Netw Open,* 6**,** e2328805.

Kasza, K. A., Rivard, C., Goniewicz, M. L., Fong, G. T., Hammond, D., Cummings, K. M. & Hyland, A. 2024. E-Cigarette Characteristics and Cigarette Cessation among Adults Who Use E-Cigarettes. *JAMA Network Open,* 7**,** e2423960.

Kasza, K. A., Tang, Z., Benson, A. F., Creamer, M. R., Edwards, K. C., Sharma, E., Chang, J. T., Cheng, Y. C., Clement, J., Cunningham, C., Ellison, C. D., Ergun, M. A., Greenberg, M., Oniyide, O., Tashakkori, N. A., Xiao, H., Stanton, C., Kimmel, H. L., Compton, W. & Hyland, A. 2025. Trends in cigarette discontinuation rates in the U.S. across 2014/15-2021 among adults who use electronic nicotine delivery systems (ENDS): Trends by ENDS flavor, ENDS device type, and age group from the PATH Study. *Nicotine & tobacco research : official journal of the Society for Research on Nicotine and Tobacco*.

Keller-Hamilton, B., Sloan, R., Mehta, T., Hinton, A., Tackett, A. P., Roberts, M. E., Brinkman, M. C. & Wagener, T. L. 2025. An observational human laboratory assessment of nicotine delivery, vaping topography and subjective effects of usual brand electronic cigarette use among young adults. *Addiction (Abingdon, England),* 120**,** 503-513.

Kirby, T. 2024. Australia to ban disposable vapes in 2024. *The Lancet. Respiratory medicine,* 12**,** e12.

Kirsch, F., Kröner, E. M., Lange, R., Niemeyer, J., Laible, M., Seidel, J., Paulus, K., Stephani, A., Tamosaite, S. & Dünnebier, K. 2024. Official test results of disposable e-cigarettes from 2022 in Germany. *Journal of Consumer Protection and Food Safety,* 19**,** 339-348.

Klosterhalfen, S., Viechtbauer, W. & Kotz, D. 2025. Disposable e-cigarettes: Prevalence of use in Germany from 2016 to 2023 and associated user characteristics. *Addiction (Abingdon, England),* 120**,** 557-567.

Kmietowicz, Z. 2024. Disposable vapes to be banned in England next summer. *BMJ (Clinical research ed.),* 387**,** q2356.

Kong, G., Lee, J., Ouellette, R. R. & Morean, M. E. 2024. Modification of Electronic Nicotine Delivery Systems Among Adolescents and Young Adults. *Pediatrics,* 154**,** e2023063984.

Lanza, H. I., Leventhal, A. M., Cho, J., Braymiller, J. L., Krueger, E. A., McConnell, R. & Barrington-Trimis, J. L. 2020. Young adult e-cigarette use: A latent class analysis of device and flavor use, 2018-2019. *Drug and alcohol dependence,* 216**,** 108258.

Lee, J., Kong, G., Krishnan-Sarin, S. & Camenga, D. R. 2024. Unintended exposure to e-liquids and subsequent health outcomes among US youth and adults. *PLoS ONE,* 19**,** e0308006.

Lee, J. & Su, W. C. 2026. A new aerosol respiratory deposition approach: Health risks of metals in aerosols from electronic nicotine delivery systems and heated tobacco products. *Journal of Aerosol Science,* 191**,** 106709.

Leigh, N. J., Page, M. K., Jamil, H. & Goniewicz, M. L. 2025. Characteristics and ingredients of disposable 'Elfbar' e-cigarettes sold in the United States and the United Kingdom. *Addiction (Abingdon, England),* 120**,** 496-502.

Leventhal, A., Dai, H., Barrington-Trimis, J. & Sussman, S. 2023. 'Ice' flavoured e-cigarette use among young adults. *Tob Control,* 32**,** 114-117.

Li, D., Ossip, D. J., Travers, M. B.-. & Xie, Z. 2022. Impact of the FDA flavour enforcement policy on flavoured electronic cigarette use behaviour changes. *Tob Control,* 31**,** s176–s183.

Li, W., Davis, D. R., Buta, E., Gueorguieva, R., Khan, N., Green, B. G., Garrison, K. A. & Krishnan-Sarin, S. 2025. "Ice" flavor elements in E-cigarette images: Influence on liking and intention to use among youth who are at risk of E-cigarette use. *Drug and Alcohol Dependence,* 269**,** 112600.

Lin, C., Baiocchi, M. & Halpern-Felsher, B. 2020. Longitudinal trends in e-cigarette devices used by Californian youth, 2014-2018. *ADDICTIVE BEHAVIORS,* 108.

Lin, H. C., Buu, A. & Su, W. C. 2022. Disposable E-Cigarettes and Associated Health Risks: An Experimental Study. *Int J Environ Res Public Health,* 19.

Lin, M. Y., Abdelfattah, L. I., Hanchate, A. D., Sutfin, E. L. & Denlinger-Apte, R. L. 2026. State-Level Flavored E-Cigarette Bans and Initiation Rates Among Youths and Adults. *JAMA network open,* 9**,** e2551744.

Liu, J., McCauley, D., Gaiha, S. M. & Halpern-Felsher, B. 2024. Perceptions of Harm and Addictiveness for Nicotine Products, THC e-Cigarettes, and e-Cigarettes with Other Ingredients Among Adolescents, Young Adults, and Adults. *Substance use & misuse,* 59**,** 2126-2136.

Liu, J., Reynolds, M. J., Hartman, L., Gottlieb, M., Coogan, J. M. & Winickoff, J. P. 2022. The First State Tobacco Flavor Ban: High School Tobacco Control Needs in a Changing Landscape. *J Sch Health,* 92**,** 720-727.

Lomngam, P., Hiscock, R., Evans-Reeves, K. & Matthes, B. K. 2025. The tobacco industry at the health-environment nexus: a framing analysis of the UK 'disposable' e-cigarette ban. *Health promotion international,* 40.

Ma, S., Ahn, S., Kaareen, A., Yang, Q., Qiu, Z., Chen, J., Berman, M., Wagener, T. & Shang, C. 2025. Features of disposable e-cigarettes and their association with pricing and consumer preference: Evidence from web data of US online stores. *Addiction (Abingdon, England),* 120**,** 514-523.

Manton-Roseblade, E. 2025. Will banning disposable vapes result in 200000 more smokers? *BMJ (Clinical research ed.),* 389**,** r1194.

Martinez, B., Son, Y. & Khlystov, A. 2025. Evaluation of carbonyl collection methods in electronic cigarette aerosols. *Frontiers in chemistry,* 13**,** 1694858.

Massey, Z. B., Fairman, R. T., Cham, B., Alqahtani, M., Luo, R., Weaver, S., Popova, L. & Ashley, D. L. 2025. Types of electronic nicotine delivery systems (ENDS) used by people of different age and smoking status groups: results from a US nationally representative survey. *BMJ Open,* 15**,** e087775.

Mattingly, D. T., Agbonlahor, O., Richardson, M., Rayens, M. K., Rose, S. W. & Hart, J. L. 2025. Factors associated with disposable electronic cigarette use among US youth: A national repeated cross-sectional study, 2021-22. *Addiction (Abingdon, England),* 120**,** 432-438.

McCauley, D. M., Baiocchi, M., Gaiha, S. M. & Halpern-Felsher, B. 2024. Sociodemographic differences in use of nicotine, cannabis, and non-nicotine E-cigarette devices. *Drug and Alcohol Dependence,* 255**,** 111061.

McCauley, D. M., Gaiha, S. M. & Halpern-Felsher, B. 2023. 169. Developmental and Racial/Ethnic Differences in Use of Nicotine, Non-Nicotine, and THC E-cigarettes. *Journal of Adolescent Health,* 72**,** S95.

McDermott, M. S., East, K. A., Brose, L. S., McNeill, A., Hitchman, S. C. & Partos, T. R. 2021. The effectiveness of using e-cigarettes for quitting smoking compared to other cessation methods among adults in the United Kingdom. *Addiction,* 116**,** 2825-2836.

Minetti, E. T., Erythropel, H. C., Keith, R., Davis, D. R., Zimmerman, J. B., Krishnan-Sarin, S. & Hamburg, N. M. 2024. Cardiovascular Health Effects and Synthetic Cooling Agents in E-cigarettes Labeled as 'clear' Marketed in Massachusetts After the Tobacco Product Flavoring Ban. *medRxiv*.

Minetti, E. T., Erythropel, H. C., Keith, R., Davis, D. R., Zimmerman, J. B., Krishnan-Sarin, S. & Hamburg, N. M. 2025. Cardiovascular Health Effects and Synthetic Cooling Agents in E-Cigarettes Labeled as "Clear" Marketed in Massachusetts After the Tobacco Product Flavoring Ban. *J Am Heart Assoc,* 14**,** e036106.

Mulu, B. A., Berg, C. J., Levine, H., Abroms, L. C., Wang, Y. & Bar-Zeev, Y. 2025. Factors associated with using disposable versus non-disposable electronic cigarettes among adults in the U.S. and Israel: a cross-sectional study with policy implications. *Israel Journal of Health Policy Research,* 14**,** 73.

Murphy, S. 2023. What e-cigarette factors determine oral epithelial DNA damage in consumers? *Evid Based Dent,* 24**,** 163-164.

Murthy, D., Keshari, S., Arora, S., Yang, Q., Loukas, A., Schwartz, S. J., Harrell, M. B., Hebert, E. T. & Wilkinson, A. V. 2024. Categorizing E-cigarette-related tweets using BERT topic modeling. *Emerging Trends in Drugs, Addictions, and Health,* 4**,** 100160.

Nian, Q., Cohen, J. E., Sinamo, J., Crespi, E., Zaman, R. & Hardesty, J. J. 2024. Heterogeneity in sociodemographic characteristics of people who use different ENDS devices. *Preventive Medicine Reports,* 37**,** 102561.

Nian, Q., Hardesty, J. J., Crespi, E. & Cohen, J. E. 2025. Use of multiple devices among U.S. adults frequently using ENDS in 2023. *Drug and Alcohol Dependence,* 268**,** 112580.

Notley, C., Varley, A., Pope, I., Dawkins, L. & Ward, E. 2025. Young people's use of disposable vapes: A qualitative study. *Addiction (Abingdon, England),* 120**,** 458-467.

Nottage, M. K., Taylor, E. V., East, K. A., McNeill, A., Thrasher, J. F., Reid, J. L., Hammond, D. & Simonavicius, E. 2025. Packaging of disposable vaping products and e-liquids in England, Canada and the United States: A content analysis. *Addiction (Abingdon, England),* 120**,** 483-495.

Omaiye, E. E., Luo, W., McWhirter, K. J., Pankow, J. F. & Talbot, P. 2022a. Disposable Puff Bar Electronic Cigarettes: Chemical Composition and Toxicity of E-liquids and a Synthetic Coolant. *Chem Res Toxicol,* 35**,** 1344-1358.

Omaiye, E. E., Luo, W., McWhirter, K. J., Pankow, J. F. & Talbot, P. 2022b. Flavour chemicals, synthetic coolants and pulegone in popular mint-flavoured and menthol-flavoured e-cigarettes. *Tob Control,* 31**,** e3-e9.

Ouellette, R. R., Morean, M. E., Lee, J. & Kong, G. 2024. Use of ENDS devices and modifications to vape cannabis among adolescents and young adults who also vape nicotine. *Addictive Behaviors,* 157**,** 108099.

Page, M. K., Zhang, L. Y., Leigh, N. J., Goniewicz, M. L. & O'Connor, R. J. 2025. Disposable nicotine vaping products now offer adjustable levels of flavour and coolness. *Tobacco control*.

Palmer, A. M., Rojewski, A. M., Carpenter, M. J., Klemperer, E. M., Baker, N. L., Sanford, B. T. & Toll, B. A. 2024. Interest in quitting e-cigarette use by device type and smoking history in US adults. *Tobacco control,* 33**,** 537-540.

Pennington, C. & Aldave, S. H. 2025. Disposable electronic cigarettes: Chemical composition in new and used devices. *Journal of Chromatography A,* 1758**,** 466178.

Pitts, S. R., Trigger, S. & Kelley, D. E. 2024. Detecting changes in tobacco product marketplace prominence using social media, advertising, sales, and web traffic data: The example of Puff Bar in the United States tobacco marketplace from 2019 to 2021. *PLoS ONE,* 19**,** e0311723.

Pulvers, K., Jamalian, N., Suh, E., Faltaoos, P., Stewart, S. L. & Aston, E. R. 2024. Nicotine and cannabis routes of administration and dual use among U.S. young adults who identify as Hispanic, non-Hispanic Black, and non-Hispanic White. *Preventive Medicine Reports,* 48**,** 102912.

R. Miranda, A., G. Patterson, J., LaPolt, D., P. Rumano, R., M. Reynoso, A., L. Berman, M., L. Keller-Hamilton, B., E. Roberts, M., K. Ferketich, A. & J. Zettler, P. 2022. A Call for Regulatory Clarity: “Switch” and “Quit Smoking” Marketing among Online Disposable E-Cigarette Sellers. *HPHR Journal*.

Ran, S., Yang, J. J., Piper, M. E., Lin, H. C. & Buu, A. 2024. Health Risks Associated with Adopting New-Generation Disposable Products Among Young Adults Who Use E-Cigarettes. *International Journal of Environmental Research and Public Health,* 21**,** 1375.

Ranpara, A., Stefaniak, A. B., Fernandez, E. & LeBouf, R. F. 2021. Effect of Puffing Behavior on Particle Size Distributions and Respiratory Depositions From Pod-Style Electronic Cigarette, or Vaping, Products. *Front Public Health,* 9**,** 750402.

Riggs, G. & Church, T. D. 2025. Beyond Juul: The New Face of Underage Nicotine Addiction - A Survey of College Students. *Therapeutic Innovation and Regulatory Science,* 59**,** 319-327.

Robertson, N. E., Hunsaker, H. C., Yamamoto, M., Cheung, K., Poulin, B. A. & Nguyen, T. B. 2025. E-Liquid and Aerosol Characterization of Popular Disposable E-Cigarettes. *ACS omega,* 10**,** 29615-29627.

Rossheim, M. E., McDonald, K. K., Soule, E. K., Gimm, G. W., Livingston, M. D., Barnett, T. E., Jernigan, D. H. & Thombs, D. L. 2020. Electronic cigarette explosion/burn and poisoning related emergency department visits, 2018-2019. *AMERICAN JOURNAL OF EMERGENCY MEDICINE,* 38**,** 2637-2640.

Rossheim, M. E., Tillett, K. K., Vasilev, V., LoParco, C. R., Berg, C. J., Trangenstein, P. J., Andrew Yockey, R., Sussman, S. Y., Siegel, M. & Jernigan, D. H. 2024. Types and Brands of Derived Psychoactive Cannabis Products: An Online Retail Assessment, 2023. *Cannabis and Cannabinoid Research,* 9**,** 1478-1481.

Ruggiero, J. L., Voller, L. M., Shaik, J. A. & Hylwa, S. 2022. Formaldehyde in Electronic Cigarette Liquid (Aerosolized Liquid). *Dermatitis,* 33**,** 332-336.

Ruggiero, J. L., Voller, L. M., Shaik, J. A., & Hylwa, S 2022. Formaldehyde in Electronic Cigarette Liquid (Aerosolized Liquid). *Dermatitis : contact, atopic, occupational, drug,* 33**,** 332–336.

Salazar, M. R., Saini, L., Nguyen, T. B., Pinkerton, K. E., Madl, A. K., Cole, A. M. & Poulin, B. A. 2025. Elevated Toxic Element Emissions from Popular Disposable E-Cigarettes: Sources, Life Cycle, and Health Risks. *ACS central science,* 11**,** 1345-1354.

Sargent, J. D., Pratt, S. I., Brunette, M. F., Ferron, J. C., Santos, M. M. & Stoolmiller, M. 2024. Level and timing of product substitution in a trial of e-cigarettes for smokers not interested in quitting. *Tobacco induced diseases,* 22.

Sargent, J. D., Stoolmiller, M., Dai, H., Barrington-Trimis, J. L., McConnell, R., Audrain-McGovern, J. & Leventhal, A. M. 2022. First E-Cigarette Flavor and Device Type Used: Associations With Vaping Persistence, Frequency, and Dependence in Young Adults. *Nicotine Tob Res,* 24**,** 380-387.

Schiek, H., Esch, T. & Hoetger, C. 2025. Initial assessment of a novel smoking cessation program integrating app-based behavioral therapy and an electronic cigarette: results of a pilot study. *Addiction science & clinical practice,* 20**,** 31.

Schiek, H., Esch, T., Michaelsen, M. M. & Hoetger, C. 2024. Combining app-based behavioral therapy with electronic cigarettes for smoking cessation: a study protocol for a single-arm mixed-methods pilot trial. *Addiction science & clinical practice,* 19**,** 52.

Schillo, B., Seaman, E. L., Cuccia, A., Ali, F. R. M., Cordova, J., Mills, S. & Kreslake, J. 2023. Early evidence of flavored tobacco product restrictions in Massachusetts and New York State. *Tob Induc Dis,* 21**,** 140.

Schillo, B. A., Bertrand, A., Briggs, J., Kierstead, E. C., Silver, N. A., Yoon, S. N. & Diaz, M. C. 2024. Analysis of e-cigarette warning letters issued by the Food and Drug Administration in 2020 and 2021. *Tobacco control,* 33**,** 247-251.

Sharma, E., Dubsky, J., Garcia-Rosales, K. V., Halenar, M., Benson, A. F., Maki, A., Ruybal, A. L., Morse, A. L., Sawdey, M. D., Alrefai-Kirkpatrick, R., Skara, S., Ntim, S. A., Chang, H. W., Aung, T. W., Kimmel, H. L., Pearson, J. L., Bansal-Travers, M., Taylor, K. A., Hyland, A. & Novotny, T. E. 2025. Disposal practices of cigarettes and electronic nicotine products among adults, findings from Wave 6 (2021) of the PATH Study. *PLOS ONE,* 20**,** e0338007.

Shroff, S. M. & Sreeramareddy, C. T. 2024. Marketing claims, promotional strategies, and product information on Malaysian e-cigarette retailer websites-a content analysis. *Substance abuse treatment, prevention, and policy,* 19**,** 11.

Smiley, S. L., Cho, J., Blackman, K. C. A., Cruz, T. B., Pentz, M. A., Samet, J. M. & Baezconde-Garbanati, L. 2021. Retail Marketing of Menthol Cigarettes in Los Angeles, California: a Challenge to Health Equity. *Preventing chronic disease,* 18**,** E11-E11.

Smith, M. J., Vaczy, C. & Hilton, S. 2025. Co-production of a youth advocacy video on the harms of e-cigarette advertising in Scotland. *Health promotion international,* 40.

Soule, E. K., Mayne, S., Snipes, W., Do, E. K., Theall, T., Hochsmann, C., Talih, S., Martin, C. K., Eissenberg, T. & Fuemmeler, B. F. 2023a. Electronic Cigarette Nicotine Flux, Nicotine Yield, and Particulate Matter Emissions: Impact of Device and Liquid Heterogeneity. *Nicotine Tob Res,* 25**,** 412-420.

Soule, E. K., Mayne, S., Snipes, W., Do, E. K., Theall, T., Höchsmann, C., Talih, S., Martin, C. K., Eissenberg, T. & Fuemmeler, B. F. 2023b. Electronic Cigarette Nicotine Flux, Nicotine Yield, and Particulate Matter Emissions: Impact of Device and Liquid Heterogeneity. *Nicotine Tob Res,* 25**,** 412-420.

Sousan, S., Pender, J., Streuber, D., Haley, M., Shingleton, W. & Soule, E. 2022. Laboratory Determination of Gravimetric Correction Factors for Real-time Area Measurements of Electronic Cigarette Aerosols. *Aerosol Sci Technol,* 56**,** 517-529.

Su, W. C., Lin, Y. H., Wong, S. W., Chen, J. Y., Lee, J. & Buu, A. 2021. Estimation of the dose of electronic cigarette chemicals deposited in human airways through passive vaping. *J Expo Sci Environ Epidemiol,* 31**,** 1008-1016.

Svarch-Perez, A., Paz-Gonzalez, M. V., Ruiz-Juarez, C., Olvera-Chacon, J. C., Larios-Solis, A., Castro-Gaytan, S., Aldeco-Perez, E. & Alcocer-Varela, J. C. 2024. Methods for a Non-Targeted Qualitative Analysis and Quantification of Benzene, Toluene, and Xylenes by Gas Chromatography-Mass Spectrometry of E-Liquids and Aerosols in Commercially Available Electronic Cigarettes in Mexico. *International Journal of Environmental Research and Public Health,* 21**,** 1308.

Syrjanen, R., Schumann, J. L., Castle, J. W., Sharp, L., Griffiths, A., Blakey, K., Dutch, M., Maplesden, J. & Greene, S. L. 2024. Protonitazene detection in two cases of opioid toxicity following the use of tetrahydrocannabinol vape products in Australia. *Clinical Toxicology,* 62**,** 539-541.

Tackett, A. P., Han, D. H., Peraza, N., Whaley, R. C., Mason, T., Cahn, R., Hong, K., Pang, R., Monterosso, J., Page, M. K., Goniewicz, M. L. & Leventhal, A. M. 2025. Effects of 'Ice' flavoured e-cigarettes with synthetic cooling agent WS-23 or menthol on user-reported appeal and sensory attributes. *Tobacco control,* 34**,** 175.

Taleb, Z. B., Dabroy, D., Akins, J., Nelson, M. D., Kalan, M. E., Rezk-Hanna, M. & Matthew Brothers, R. 2023. Pod-based e-cigarettes versus combustible cigarettes: The impact on peripheral and cerebral vascular function and subjective experiences. *Tobacco Induced Diseases,* 21**,** 71.

Talih, S., Karaoghlanian, N., Salman, R., Hilal, E., Patev, A., Bell, A., Fallah, S., El-Hage, R., Saliba, N. A., Cobb, C., Barnes, A. & Shihadeh, A. 2023. Effects of Aftermarket Electronic Cigarette Pods on Device Power Output and Nicotine, Carbonyl, and ROS Emissions. *Chem Res Toxicol,* 36**,** 1930-1937.

Talih, S., Salman, R., Soule, E., El-Hage, R., Karam, E., Karaoghlanian, N., El-Hellani, A., Saliba, N. & Shihadeh, A. 2022. Electrical features, liquid composition and toxicant emissions from 'pod-mod'-like disposable electronic cigarettes. *Tob Control,* 31**,** 667-670.

Tattan-Birch, H., Brown, J., Shahab, L., Beard, E. & Jackson, S. E. 2024. Trends in vaping and smoking following the rise of disposable e-cigarettes: a repeat cross-sectional study in England between 2016 and 2023. *The Lancet Regional Health - Europe,* 42**,** 100924.

Tattan-Birch, H., Brown, J., Shahab, L. & Jackson, S. E. 2021. Trends in use of e-cigarette device types and heated tobacco products from 2016 to 2020 in England. *SCIENTIFIC REPORTS,* 11.

Tattan-Birch, H., East, K., Cox, S., Jackson, S., Brown, J., Simonavicius, E., Reid, J. L., Hammond, D. & Taylor, E. V. 2025. Impact of standardising the colour and branding of vape devices on product appeal among young people: a randomised experiment in England, Canada and the United States. *Tobacco control*.

Taylor, E., Ebdon, M., Nottage, M., Simonavicius, E., Brose, L., McNeill, A., Arnott, D., Cheeseman, H., Bunce, L. & East, K. 2025a. The effect of standardised packaging and limited flavour descriptors of vape pods among adults and youth in Great Britain: a cross-sectional between-subjects experimental study. *The lancet regional health - europe,* 58.

Taylor, E., Robson, D., Brose, L. S., Hammond, D., Reid, J. L., Block, A. C., Goniewicz, M. L., Nicolaidu, M. & McNeill, A. 2025b. Nicotine and tobacco-specific nitrosamine exposure among youth in England who smoke cigarettes and/or vape. *Tobacco control*.

Taylor, E., Tattan-Birch, H. & East, K. 2025c. England's disposable vape ban: An inadequate solution to youth vaping with potential unintended consequences. *Addiction (Abingdon, England),* 120**,** 402-404.

Tehrani, M. W., Newmeyer, M. N., Rule, A. M. & Prasse, C. 2021. Characterizing the Chemical Landscape in Commercial E-Cigarette Liquids and Aerosols by Liquid Chromatography-High-Resolution Mass Spectrometry. *Chem Res Toxicol,* 34**,** 2216-2226.

Timmerman, A., Deventer, M., Andrews, R., Reid, R., Marland, V., Edwards, D., Pudney, C., NicDaeid, N., Stove, C. & Norman, C. 2025. Waxy- or Putty-Like Materials as a Novel Drug Preparation for Synthetic Cannabinoid Receptor Agonists: Detection in Prisons and In Vitro Cannabinoid Receptor Activity. *Drug Testing and Analysis,* 17**,** 1118-1130.

Tran, P., Jayawardene, W. & DeSalle, M. 2021. E-cigarettes users' quit methods and interventions that may facilitate quitting. *Journal of Addiction Medicine,* 15**,** E17.

Turner, A., Scott, J. W., Backshall-Kennedy, T. & Dabrowski, M. C. 2024. Deconstructing contemporary disposable vapes: A material and elemental analysis. *Science of the Total Environment,* 954**,** 176292.

Vassey, J., Kennedy, C. J., Chang, H. C. H., Smith, A. S. & Unger, J. B. 2024. Scalable Surveillance of E-Cigarette Products on Instagram and TikTok Using Computer Vision. *Nicotine and Tobacco Research,* 26**,** 552-560.

Vilcassim, M. J. R., Jacob, D., Stowe, S., Fifolt, M. & Zierold, K. M. 2023. Sex Differences in Electronic Cigarette Device Use Among College Students. *J Community Health,* 48**,** 585-592.

Vilcassim, M. J. R., Stowe, S. & Zierold, K. M. 2025. Perception of Health Risks of Electronic Cigarette Use Among College Students: Examining the Roles of Sex, Field of Study, Vaping Device Type, and Their Associations. *Journal of community health,* 50**,** 23-30.

Virgili, F., Del Parco, F., La Regina, D. P., Mancino, E., Petrarca, L., Conti, M. G., Bonci, E., Nenna, R. & Midulla, F. 2025. Prevalence, risk perception, and motivations behind E-cigarettes and heated tobacco use: a cross-sectional study in Italian adolescents. *Italian Journal of Pediatrics,* 51**,** 303.

Wang, P., Williams, R. J., Chen, W., Wang, F., Shamout, M., Tanz, L. J., Herzig, C. T. A., Oakley, L. P., Peak, C. M., Heinzerling, A., Al-Shawaf, M., Melstrom, P., Marynak, K., Tynan, M. A., Agaku, I. T. & Kumagai, K. 2024. Chemical Composition of Electronic Vaping Products From School Grounds in California. *Nicotine and Tobacco Research,* 26**,** 991-998.

Wang, S., Nighbor, T., Westmaas, J. L., Asare, S., Patel, M. & Nargis, N. 2026. Prevalence and Sources of E-Cigarette Use: Findings From a Rapid-Response Survey in High-Tobacco-Burden U.S. States, 2023-2024. *American Journal of Preventive Medicine,* 70**,** 108062.

Wang, T. W., Gentzke, A. S., Neff, L. J., Glidden, E. V., Jamal, A., Park-Lee, E., Ren, C., Cullen, K. A., King, B. A. & Hacker, K. A. 2021. Characteristics of e-Cigarette Use Behaviors Among US Youth, 2020. *JAMA Netw Open,* 4**,** e2111336.

Wang, X., Ghimire, R., Shrestha, S. S., Borowiecki, M., Emery, S. & Trivers, K. F. 2023. Trends in Nicotine Strength in Electronic Cigarettes Sold in the United States by Flavor, Product Type, and Manufacturer, 2017-2022. *Nicotine Tob Res,* 25**,** 1355-1360.

Watkins, S. L., Snodgrass, K., Fahrion, L. & Shaw, E. 2025. Contextualizing Changes in e-Cigarette Use During the Early COVID-19 Pandemic and Accompanying Infodemic ("So Much Contradictory Evidence"): Qualitative Document Analysis of Reddit Forums. *Journal of Medical Internet Research,* 27**,** e66010.

Yingst, J. M., Bordner, C. R., Hobkirk, A. L., Hoglen, B., Allen, S. I., Krebs, N. M., Houser, K. R., Livelsberger, C. & Foulds, J. 2021. Response to Flavored Cartridge/Pod-Based Product Ban among Adult JUUL Users: "You Get Nicotine However You Can Get It". *INTERNATIONAL JOURNAL OF ENVIRONMENTAL RESEARCH AND PUBLIC HEALTH,* 18.

Yogeswaran, S., Muthumalage, T. & Rahman, I. 2021. Comparative Reactive Oxygen Species (ROS) Content among Various Flavored Disposable Vape Bars, including Cool (Iced) Flavored Bars. *Toxics,* 9.

Yong, H. H., Hughes, L., Borland, R., Gravely, S., Cummings, K. M., Brose, L. S., Taylor, E., Bansal-Travers, M. & Hyland, A. 2025. Prevalence and correlates of negative side effects from vaping nicotine: Findings from the 2020 ITC four country smoking and vaping survey. *Drug and Alcohol Dependence,* 266**,** 112503.

Zare, S. & Zheng, Y. 2021. Consumer Preferences for E-cigarette Flavor, Nicotine Strength, and Type: Evidence from Nielsen Scanner Data. *Nicotine Tob Res,* 23**,** 823-828.

Zhang, Q., Jeon, J., Goldsmith, T., Black, M., Greenwald, R. & Wright, C. 2023. Characterization of an Electronic Nicotine Delivery System (ENDS) Aerosol Generation Platform to Determine Exposure Risks. *TOXICS,* 11.
